# Multiplicity of Steady States in Glycolysis and Shift of Metabolic State in Cultured Mammalian Cells

**DOI:** 10.1371/journal.pone.0121561

**Published:** 2015-03-25

**Authors:** Bhanu Chandra Mulukutla, Andrew Yongky, Simon Grimm, Prodromos Daoutidis, Wei-Shou Hu

**Affiliations:** Department of Chemical Engineering and Materials Science, University of Minnesota, Minneapolis, Minnesota, United States of America; Glasgow University, UNITED KINGDOM

## Abstract

Cultured mammalian cells exhibit elevated glycolysis flux and high lactate production. In the industrial bioprocesses for biotherapeutic protein production, glucose is supplemented to the culture medium to sustain continued cell growth resulting in the accumulation of lactate to high levels. In such fed-batch cultures, sometimes a metabolic shift from a state of high glycolysis flux and high lactate production to a state of low glycolysis flux and low lactate production or even lactate consumption is observed. While in other cases with very similar culture conditions, the same cell line and medium, cells continue to produce lactate. A metabolic shift to lactate consumption has been correlated to the productivity of the process. Cultures that exhibited the metabolic shift to lactate consumption had higher titers than those which didn’t. However, the cues that trigger the metabolic shift to lactate consumption state (or low lactate production state) are yet to be identified. Metabolic control of cells is tightly linked to growth control through signaling pathways such as the AKT pathway. We have previously shown that the glycolysis of proliferating cells can exhibit bistability with well-segregated high flux and low flux states. Low lactate production (or lactate consumption) is possible only at a low glycolysis flux state. In this study, we use mathematical modeling to demonstrate that lactate inhibition together with AKT regulation on glycolysis enzymes can profoundly influence the bistable behavior, resulting in a complex steady-state topology. The transition from the high flux state to the low flux state can only occur in certain regions of the steady state topology, and therefore the metabolic fate of the cells depends on their metabolic trajectory encountering the region that allows such a metabolic state switch. Insights from such switch behavior present us with new means to control the metabolism of mammalian cells in fed-batch cultures.

## Introduction

Glucose metabolism plays a central role in supplying carbon precursors for cellular energy and biosynthetic needs. Cancer cells have elevated glucose consumption and glycolytic flux in ways similar to the response of tissues to growth promoting signals [1]. Cellular glucose metabolism is subjected to vast interacting regulations exerted at various levels [2–4]. At the pathway level, many enzymatic steps are controlled through feedback and feed-forward allosteric regulation by metabolic intermediates [5]. The regulatory effectors and control action on the enzyme kinetics differ for different isozymes catalyzing the same reaction step. Cells in different tissues and even cells at different disease or development stages, may express different isozymes to meet their cellular demands [6, 7]. Additionally, through signaling pathways, glycolysis activity is tied to growth control [2, 3]. In the past decade there has been an increasing interest in controlling a cell’s disease state, for instance to minimize uncontrolled proliferation through modulation of cellular metabolism.

The high rates of glucose consumption and lactate production seen in cancer cells are also observed in other fast proliferating cells, such as mammalian cell lines in culture. The accumulation of lactate in culture has long been recognized as an inhibitory factor for cell growth and recombinant protein production [8, 9]. In the past two decades fed-batch cultures have become extensively used in cell culture bioprocessing. The total amount of glucose added to the medium over the culture period is far higher than the range commonly seen in typical culture media. Lactate accumulation seen in cultures also greatly exceeds the physiological level. Cells in the late stages of their growth in a fed-batch culture sometimes switch their metabolism from lactate production to low lactate production or lactate consumption [10–13]. However, such a shift in metabolism is not a consistent occurrence; under seemingly similar conditions, some cultures switch their metabolism and consume lactate while others continue to produce lactate at high rates. The metabolic shift to lactate consumption has been shown to positively correlate to higher productivity [14, 15]. Thus, a better understanding of the lactate consumption phenomena will help in contriving strategies for robust control of cell metabolism and higher protein yields.

Previously, we reported development of a mechanistic mathematical model of glycolysis and the pentose phosphate pathway to examine the dynamic behavior of glucose metabolism [5]. The model considers different isozymes of three key glycolysis enzymes (phosphofructokinase (PFK), pyruvate kinase (PK) and 6-phosphofructo-2-kinase/fructose-2,6-bisphophatase (PFKFB)) and the allosteric regulations they are subjected to by glycolytic intermediates. All three isozymes of PFK (PFKM, PFKL and PFKP) are activated by fructose-2,6-bisphosphate (F26BP) [16], but only PFKM and PFKL are activated by fructose-1,6-bisphosphate (F16BP) [17–19]. Three isozymes of PK (PKM2, PKL and PKR) are activated by F16BP to varying extents while PKM1 is not under such allosteric regulation [20]. PFKFB is a bifunctional enzyme whose kinase and bisphosphatase domains catalyze the formation and hydrolysis reaction of F26BP, respectively. The four isozymes of PFKFB (PFKFB1–4) differ in their kinase and phosphatase activities as well as in their sensitivity to feedback inhibition by phosphoenolpyruvate (PEP) [21–23]. In addition, several isozymes of PFKFB are subject to post-translational modification by hormonal and growth signaling pathways that modulate the balance between the kinase and phosphatase activities [24]. Thus, each isozyme of PFKFB has a profoundly distinct capacity in modulating PFK activity.

We demonstrated that the combination of isozymes of these three glycolytic enzymes, commonly seen in many rapidly growing cells, give rise to bistable behavior in glycolysis activity [5]. Under physiological glucose concentrations, the steady state glycolysis flux may be at a high state or a low state. Although the cells may switch their metabolism between the two flux states, the transition from a high flux state to a low flux state can only occur at glucose concentrations that are outside the physiological range. Our model prediction of bistability is consistent with the experimental observation that a shift from a high flux state to a low flux state was accomplished only by controlling glucose concentration at very low levels [5, 8, 9].

In the current study, we hypothesize that the switch of metabolism in fed-batch culture is a reflection of the bistable behavior described above. The glucose and lactate concentrations in contemporary fed-batch processes often reach levels beyond 30 mM and 100 mM, respectively [14, 15]. Such non-physiological conditions may elicit dynamic responses unseen *in vivo*. In particular the inhibitory effect of lactate on PFK that is relatively minor in most tissues *in vivo* may become prominent in fed-batch cultures due to its high level of accumulation [25].

In this work, we extend our previous modeling explorations to the previously unexplored space of glucose and lactate concentrations that spread beyond physiological levels and seek to address the important issue of the controllability of metabolic shift in biopharmaceutical manufacturing. Since lactate consumption occurs through its conversion to pyruvate and oxidation in the tricarboxylic acid (TCA) cycle, we extended our model to include the TCA cycle and the malate-aspartate shuttles. Metabolic shift in cultured cells largely occurs after the rapid growth period is over. The linkage between metabolism and growth control has been a subject of intense research in the past decade. The v-akt murine thymoma viral oncogene homolog (AKT), also known as protein kinase B (PKB), is a serine/threonine kinase that plays a key role in multiple cellular processes including cell proliferation and glucose metabolism (for reviews, see [2–4]). AKT exists in an active/phosphorylated (pAKT) form and an inactive/unphosphorylated form. The AKT signaling cascade has been shown to activate the transcription of GLUT1 [26] and mediates the association of hexokinase 1 and 2 (HK1 and HK2) with outer mitochondrial membrane [27, 28]. In addition, pAKT can increase the phosphorylation of PFKFB to shift its kinase/phosphatase ratio to increase the formation of fructose 2,6-bisphosphate levels [22, 29, 30], which in turn increases PFK activity and glycolysis flux. A decrease in the growth rate of the cells observed during the course of the culture is accompanied by the decrease in the pAKT levels [10]. Thus, in the extended model the effect of cell growth rate on glycolysis flux is dealt with the dependence of the kinase activity of the bifunctional enzyme PFKFB on pAKT levels. We report herein that our extended model for cell metabolism reveals multiplicity of steady states of glycolysis activity and the modulation of the flux topology by three culture parameters, namely glucose, lactate and growth rate (in the form of pAKT levels). Such an understanding of the metabolic flux topology can help control the metabolic state and enhance process robustness.

## Materials and Methods

### Mathematical Model of Central Metabolism Pathway

**Fig 1:**
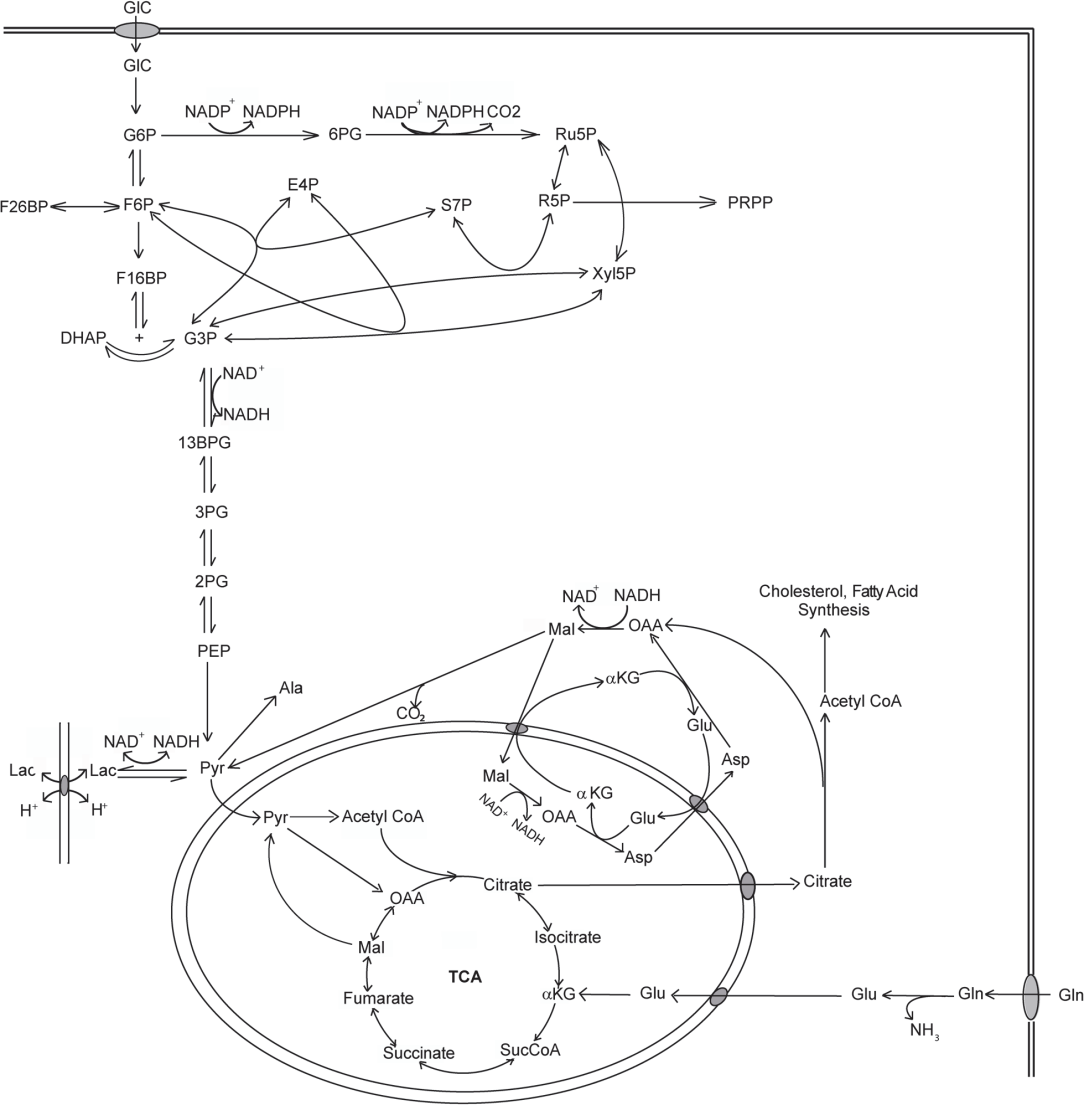
Kinetic model of the central metabolic pathways. The pathways include glycolysis, TCA cycle, pentose phosphate pathway (PPP) and the NAD/NADH shuttles between cytosol and mitochondria. S1 Table contains the list of the abbreviations.

Previously, a kinetic metabolic model of mammalian metabolism including glycolysis and pentose phosphate pathway was constructed [5]. In this study the model was extended to include the TCA cycle, malate-aspartate shuttle and citrate shuttle (between the cytosol and the mitochondria compartments) (Fig. 1). The extension is necessary for consideration of lactate uptake from extracellular milieu and its further oxidation in mitochondria. The ordinary differential equation (ODE) model consists of mass balance equations of the 40 reaction intermediates in glycolysis, the TCA cycle, the pentose phosphate pathway, components of the malate-aspartate shuttle and other inter-compartmental shuttles. The levels of enzymes are obtained from their corresponding transcript levels in cultured cells, assuming that the protein level is proportional to the transcript [10]. The rate expressions for all enzymatic reactions are based on a mechanistic derivation [31] and are listed in the Rate Equations section of the S1 File. The abbreviations for all enzymes and metabolites are listed in S1 Table.

**Fig 2:**
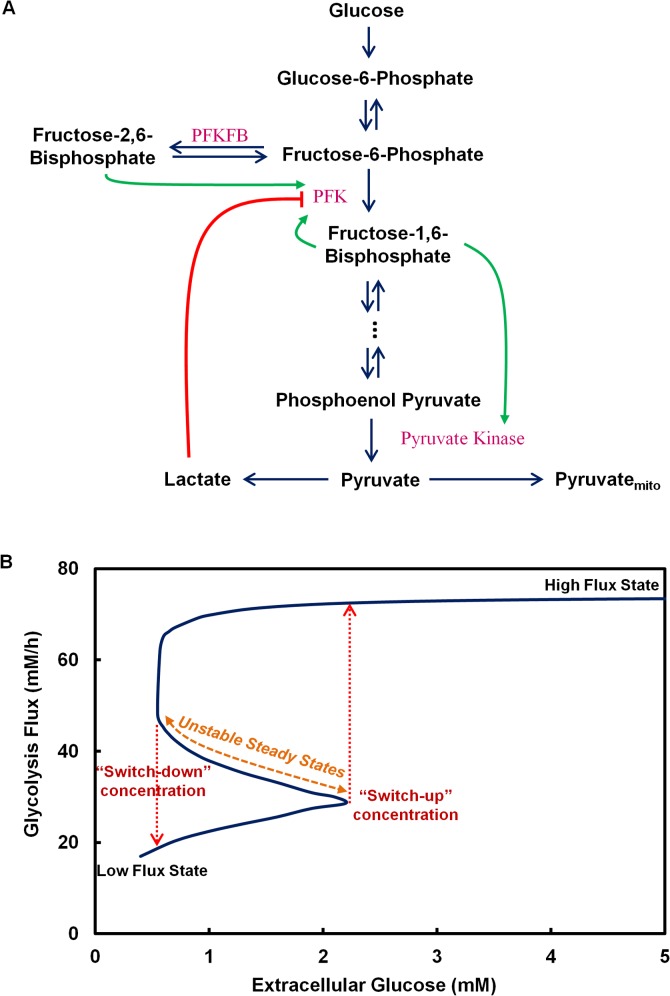
Multiplicity of steady states in glycolytic activity. (A) Isozymes of glycolysis are subject to multiple allosteric regulations (B) Bistability in glycolysis activity.

Many reactions are catalyzed by isozymes which differ not only in rate constants but also in the type of the regulatory mechanisms they are subjected to. The allosteric regulations of the isozymes of PFK, PFKFB and PK are considered in detail based on the Monod-Wyman-Changeaux method [32]. The transcript levels of glycolytic isozymes across different CHO cell lines from our archived microarray data were surveyed to identify the dominating isoform and to provide a range of physiological enzyme levels (S1 Fig. and S2 Table). When multiple isozymes are expressed in a cell line, the dominant form was used in the model. Specifically, the muscle isoform of PFK (PFKM) and the M2 isoform of pyruvate kinase (PKM2), both of which show high sensitivity to activation by F16BP, are considered. In addition, the HK1 isoform of hexokinase and the PFKFB3 isoform of PFKFB which shows the highest kinase/phosphatase (K/P) ratio were considered due to their abundance in CHO. All the kinetic parameters have been reported previously in literature and are described in detail in the S1 File. The allosteric regulations which are active in CHO cells are shown in Fig. 2A.

The model also incorporates the malate-aspartate shuttle pathway which transfers the reducing potential of cytosolic NADH into mitochondria. The malate-aspartate shuttle pathway plays an important role in maintaining the balance of NAD/NADH in the cytosolic compartment and is especially critical for the switch of metabolic state. Its incorporation is also critical because it removes the necessity to estimate the NAD/NADH levels in the two metabolic states. The transport of pyruvate and other metabolites including citrate and glutamate into mitochondria are modeled as simple mass action kinetics driven by concentration gradients. Glutamine supply into the TCA cycle is represented through two reactions catalyzed by the enzymes glutaminase (GLS) and glutamate dehydrogenase (GDH).

The regulation of glycolysis flux by growth control is modeled as pAKT activation of the kinase activity of the bifunctional enzyme PFKFB using an empirical formulation depicting an increasing kinase activity with increasing pAKT in a saturation type of kinetics (Eq. S4 in S1 File). A similar expression has been used previously to describe the effect of AKT on glycolysis [33, 34].

### Simulation

#### Steady state solution

An algebraic model consisting of steady state mass balance equations for the intermediates of all the reactions considered was derived from the ODE model. The algebraic model was used to evaluate all the possible steady states and their corresponding eigenvalues. The inputs for the model are the concentrations of glucose, lactate and pAKT. The extracellular glutamine level was fixed at 4 mM. The intracellular concentrations of energy nucleotides (ATP, ADP, AMP, NAD+, NADH) and a number of metabolites (Acetyl-Coenzyme A, Coenzyme A, 2,3-bisphosphoglycerate, glucose 1,6-bisphosphate) were set to be constant and are listed in S3 Table. The steady state solutions were obtained using Matlab (Mathworks, Inc.) with the numerical solver *fsolve*. For each combination of glucose, lactate and pAKT concentrations, positive and real-valued solutions were calculated using initial guesses, which are pseudorandom values drawn from the standard uniform distribution. Stability analysis was performed using eigenvalue analysis for each steady state solution obtained.

A system is said to be at steady state if none of the variables defining the system’s state change in number, amount, or concentration throughout time. A steady state can be classified as stable or unstable based on the response of the system to an external perturbation. A steady state is stable if the system returns to the same steady state upon an external perturbation. In contrast, if the system moves away from its original steady state upon a slight perturbation, then the steady state is unstable. Mathematically, a steady state is stable if the eigenvalues of the system’s Jacobian are all negative. A positive eigenvalue for the Jacobian indicates the steady state to be unstable.

The intracellular metabolite concentrations at steady state were examined to ensure all concentrations were within the same order of magnitude as the physiological range. In the model simulation, the extracellular lactate concentration was kept constant to allow a steady state to be reached.

#### Transient simulation

Transient simulation was performed using the ODE solver *ode15* in Matlab. Initial extracellular glucose and lactate concentrations were specified at 35 and 5 mM, respectively. Cell concentration was assumed to be constant at 2 x10^7^ cells/mL throughout the duration of the simulation. pAKT level was held constant at 0.17 in order to confine the simulations to the moment when the metabolic fate starts to diverge. The changes in extracellular glucose, extracellular lactate and glycolysis flux were followed.

#### Sensitivity analysis

Sensitivity analyses were performed by changing, one at a time, the concentration of each enzyme in the glycolysis, TCA cycle, PPP and inter-compartmental shuttles. Sensitivity of the bistable behavior to the concentration of each individual enzyme was assessed by the range of the enzyme levels in which the bistability exists.

#### Experimentation

Recombinant Chinese hamster ovary (CHO) cells producing an immunoglobulin were grown in shake flasks at 36.5°C and 5% CO_2_ environment. All experiments utilized a custom production medium and a feed medium developed at Pfizer. The production medium was a mixture of Dulbecco’s Modified Eagle’s medium (DMEM) and Ham’s F-12 (DMEM:F12), while the feed medium was based on a 3-fold concentrate of the production medium, except that the concentration of glucose in the production medium and feed medium was 2g/L and 100g/L, respectively. Lactate was not present in the original formulation of production or feed medium. Lactate was added to different conditions at the start of the experiments at the levels specified.

In fed-batch experiments, cells were inoculated at 6x10^5^ viable cells/mL. Starting on Day 3, the cultures were fed with feed medium equivalent to 1.8% of the initial culture volume. pH was measured daily using a blood gas analyzer and adjusted to 7.2 using 1.0 M sodium carbonate (Na_2_CO_3_). Samples were taken daily for measurements of cell density, viability, glucose and lactate concentrations using a Nova Bioprofile instrument. 500 g/L glucose solution was used to supplement the cultures with glucose as and when required to maintain the glucose levels above 1.5g/L. At the start of the experiment, cultures were supplemented with indicated levels of sodium lactate. Sodium chloride was used as a substitute for sodium lactate to adjust the initial osmolarity to 300 mOsm/kg across the experimental conditions.

## Results

### Bistability in Glucose Flux in Energy Metabolism

In our previous study, we have demonstrated that a combination of isozymes confers glycolysis flux with a multiplicity of steady states [5]. We showed that the presence of multiple steady states is the result of the regulatory action of two allosteric feedback loops. Survey of transcriptome data of various CHO cell lines revealed that CHO cells typically express the same set of isozymes that confers glycolysis flux with the hallmarks of bistability (S1 Fig.). The glycolysis flux behavior of CHO cells was simulated using the metabolic model that incorporates muscle isozyme of phosphofructokinase (PFKM), M2 isozyme of pyruvate kinase (PKM2) and the brain isozyme of 6-phosphofructo-2-kinase/2,6-bisphosphatase (PFKFB3), which are the respective dominating isozymes observed in CHO cells. The multiplicity of steady states in glycolysis flux was seen in CHO cells (Fig. 2B).

In the glucose concentration range of ∼0.5–2.2 mM, three types of steady states in glycolysis flux are seen: two are stable which represent the high and the low flux states, and the middle ones are unstable. The stability of each steady state was confirmed by eigenvalue analysis. Outside this region, only one steady state is observed for a given glucose concentration; below 0.5 mM, glycolysis has only the low flux state, whereas above 2.2 mM, only the high flux state exists. In the high glycolysis flux state, glucose is consumed rapidly and lactate is produced at a high rate (Fig. 2B). In the low glycolysis flux region, glucose consumption rate is low. The behavior of lactate in the low flux region varies somewhat; it is produced at a slow rate in the bistable region, but is consumed at a low rate as glucose concentration decreases further.

In the bistable region, glycolysis can either operate at a high or a low flux state depending on the previous state of the system. The middle steady states being unstable cannot be realized experimentally. When the glucose concentration changes, the glycolysis flux changes along the stable steady state lines. Starting from a high glucose concentration (thus the high flux state), as the glucose concentration decreases, the flux remains at the high state until the concentration decreases to 0.5 mM (“switch-down” concentration, Fig. 2B), where it decreases abruptly to a low state. Further decrease in the glucose level causes the system to travel further down along the low flux steady state line. Once the system reaches a low state, it does not switch back to the high flux state at the “switch-down” concentration with small perturbation (increase) in glucose level. In order to return to the high flux state, the system must now travel along a distinct trajectory. From a low flux state, glucose concentration must increase above 2.2 mM (“switch-up” concentration, Fig. 2B), before it abruptly changes to a high flux state. Further increase in glucose results in the system to travel further up along the high flux steady state line. The system is thus marked by well separated high flux and low flux states and very distinct “switch-up” and “switch-down” glucose concentrations.

Sensitivity analysis on the multiplicity of steady states was performed by varying the level of each enzyme over the range of two orders of magnitude while holding all the other kinetic parameter values constant. Exhaustive simulation for evaluation of steady state behavior on all possible enzyme level combinations is clearly not feasible. The results of the sensitivity analysis show that multiple steady states can be seen over a wide range of enzyme levels for many enzymes except for hexokinase (HK) and pyruvate dehydrogenase (PDHC) (S4 Table).

Altogether, these results indicate the potential for two systems to be at the same extracellular glucose concentration inside the bistable region, while showing different flux behaviors depending on their histories.

### Effect of Lactate Concentration on Bistability

**Fig 3:**
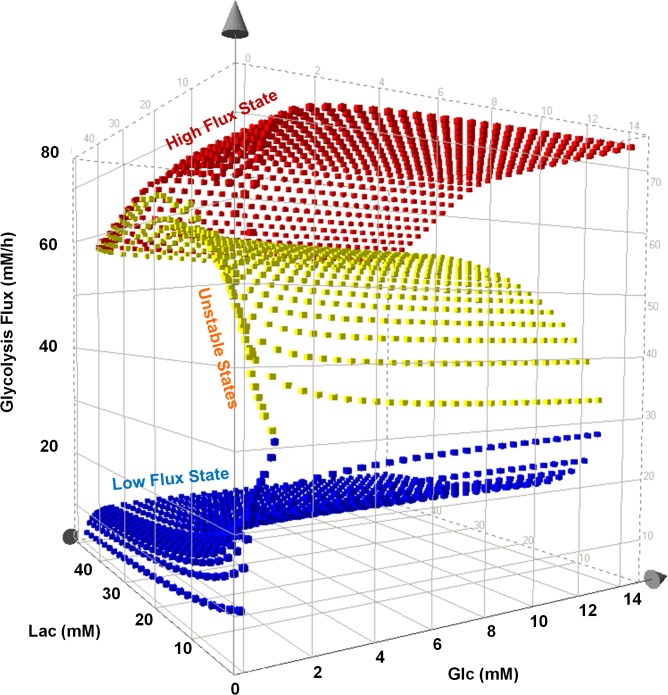
Effect of lactate on bistability. Effect of lactate on the multiplicity of states in glycolysis is examined by model simulation at various combinations of lactate (0–40 mM) and glucose (0–15 mM) concentrations typically seen in cell culture processes. The bistable behavior in glycolysis imparts more complex dynamics as lactate concentration is varied.

Lactate exerts an inhibitory effect on glycolysis flux through its feedback regulation on PFK (Fig. 2A) [25, 35]. The steady state behavior shown in Fig. 2B was obtained at a constant extracellular lactate concentration of 0.4 mM. In fed-batch cultures, lactate may accumulate to high levels that greatly exceed the physiological range. We thus examined the effect of a wide range of lactate concentration on the glycolysis flux. The results are presented in a three-dimensional plot with the flux plotted against glucose and lactate concentrations (Fig. 3). The resulting plot shows a high and a low surface representing high and low flux states, respectively. The surfaces are colored in red, blue and yellow. The top (red) surface represents the plane of high flux steady states. The bottom (blue) surface represents the plane of low flux steady states and the yellow region represents the plane of unstable steady states. A slice of the plot at a fixed lactate concentration of 0.4 mM along the glucose axis yields the curve shown in Fig. 2B. On this constant lactate plane the “switch-up” and “switch-down” points can be seen. With increasing levels of lactate, the “switch-up” glucose concentration shifts to higher glucose levels. At very high lactate concentrations (> 40 mM), the “switch-up” concentration will move to extremely high glucose concentrations that are not even seen in culture as they would represent near lethal high osmolality to cells. Even in this region of very high lactate, switching down from high state to low state is feasible if glucose concentration falls to very low levels.

### Effect of AKT on Bistability

**Fig 4:**
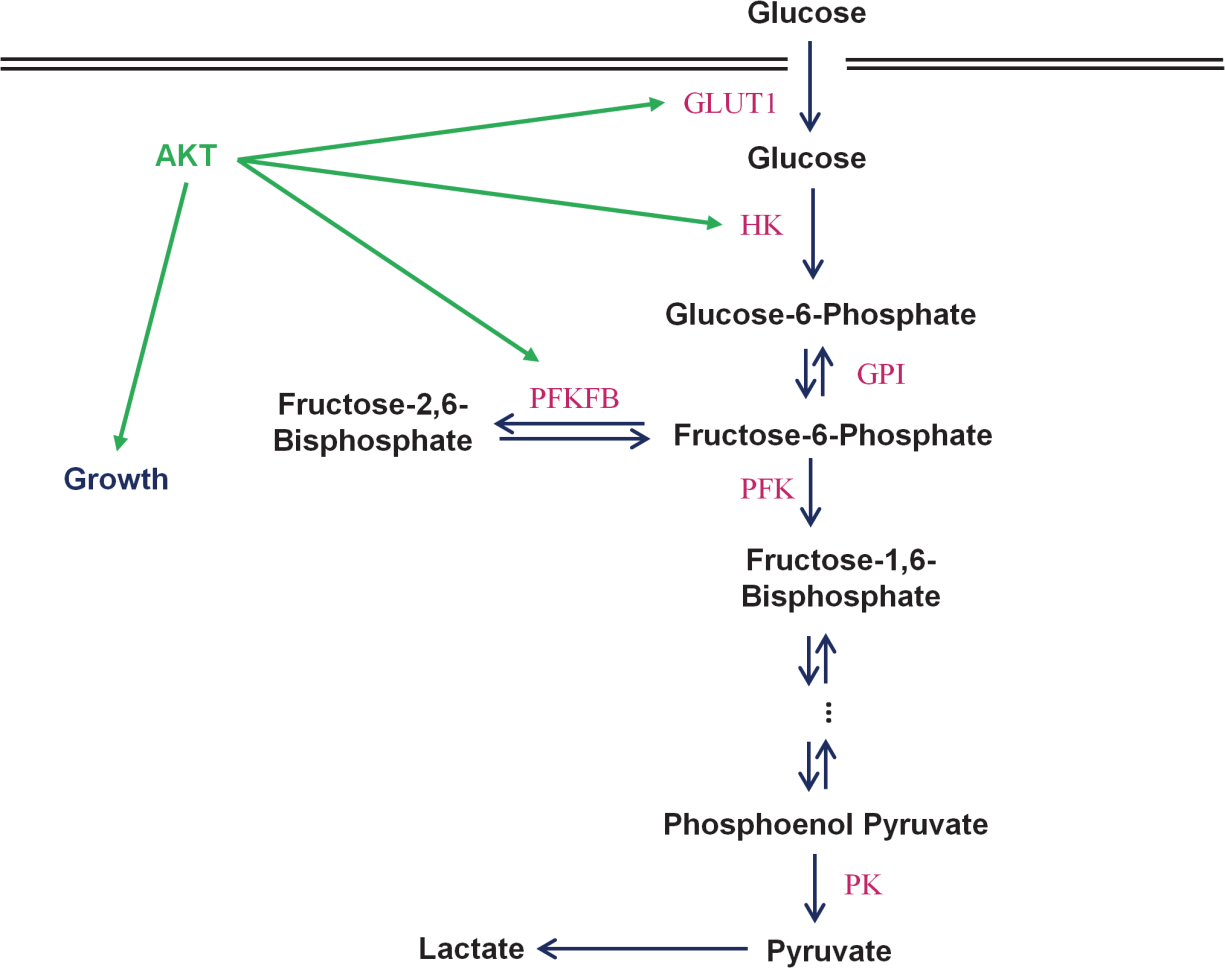
Regulations of AKT on glycolysis. The metabolic control of the cells is tightly linked to growth control through signaling pathways such as AKT signaling. The AKT signaling cascade has been shown to activate the transcription of GLUT1 and mediates the association of HK with outer mitochondrial membrane to provide direct access to ATP, as a driving force for high rate of glycolysis. In addition, AKT can phosphorylate PFKFB to shift its kinase/phosphatase ratio to increase the formation of fructose 2,6-bisphosphate levels which in turn increases PFK activity and glycolysis flux.

The AKT signaling pathway regulates the growth and proliferation of mammalian cells, and stimulates the activity of glycolysis [2, 3]. The AKT signaling cascade induces the transcription of the glucose transporter GLUT1 and mediates the association of HK1–2 with outer mitochondrial membrane (Fig. 4). In addition, the active form pAKT has the capability to phosphorylate the bifunctional enzyme PFKFB to enhance its kinase activity [22, 29, 30] resulting in an increase of F26BP production and a further increase of the glycolysis flux. Thus, as the growth rate of the cells slows down, the simultaneous decrease in the pAKT level causes glycolysis activity to decrease. This was observed in the late stages of fed-batch cultures [10]. Among these three targets regulated by pAKT, PFKFB3 is stimulated at its activity level whereas the other two (GLUT1 and HK1) are regulated at the transcriptional or localization level. For stability and steady state behavior, our focus is on the system in which enzyme levels are kept constant and uniformly distributed inside the cells. Our investigation on the effect of growth on glycolysis flux is thus focused on the regulation of pAKT on PFKFB3.

**Fig 5:**
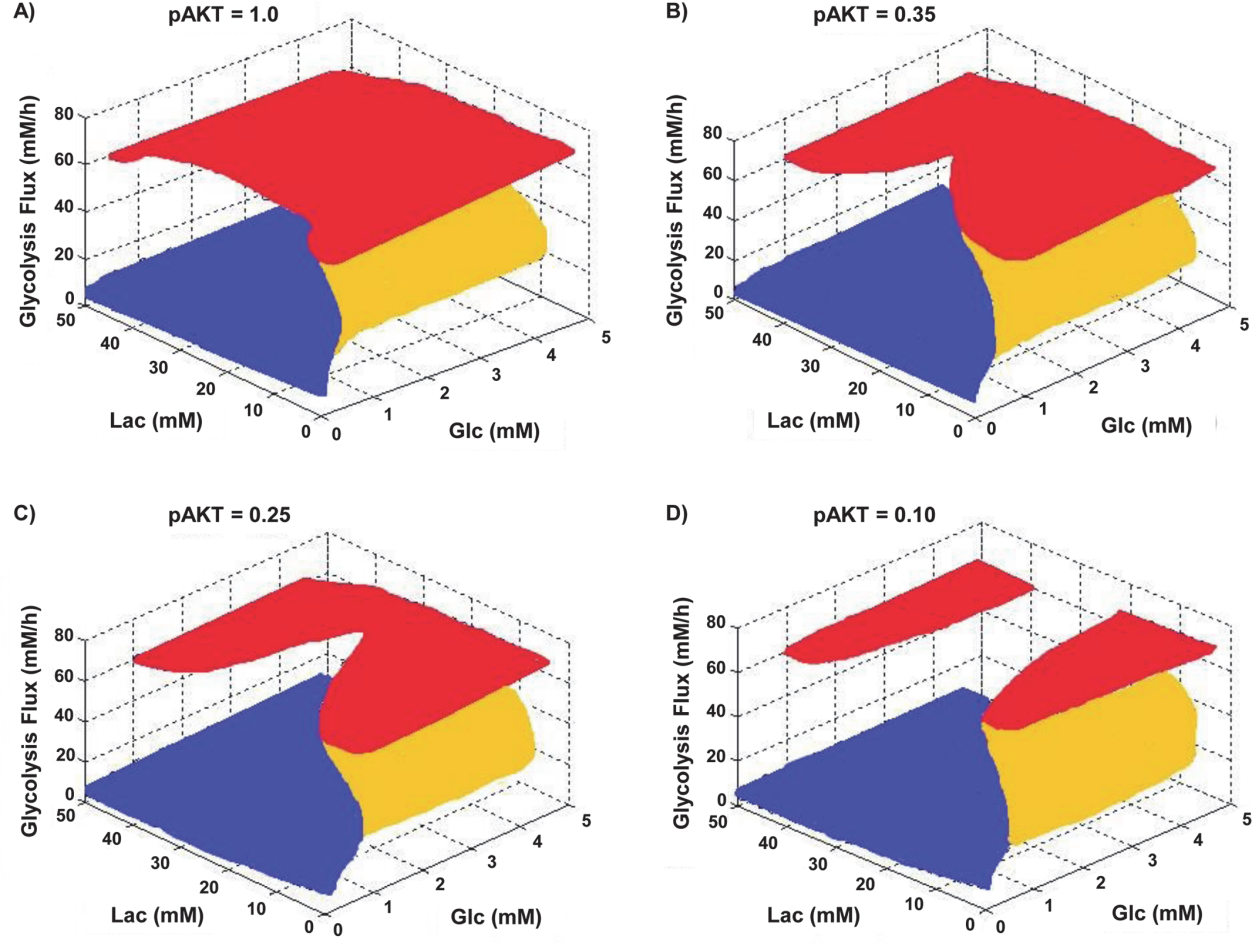
Effect of glucose and lactate on glycolytic flux at different AKT activity levels. The red and blue surfaces are stable steady states representing the high and low glycolytic flux states, respectively. Yellow surfaces are unstable steady states representing the intermediate glycolytic flux states. (A) pAKT = 1.00 (B) pAKT = 0.35 (C) pAKT = 0.25 (D) pAKT = 0.10.

Four discrete values of pAKT levels were examined and the steady-state behavior of glycolysis flux at various glucose and lactate concentrations are shown in Fig. 5A–5D. At a high pAKT level (pAKT = 1, Fig. 5A), a shift from the high flux plane to the low flux plane occurs only at very low glucose concentration (∼1 mM) for the entire lactate concentration range examined. However, with lower pAKT levels, a section of the top plane recedes towards higher glucose concentration such that a metabolic shift from the high flux plane to the low flux plane is possible at higher glucose concentrations (Fig. 5B–5D). The section of the top plane that regresses is confined to a small range of lactate concentration (∼15–40 mM).

### Trajectory of the Metabolic Shift

In a fed-batch culture, glucose is added intermittently to sustain its concentration within a given range. Lactate is produced at a high specific rate in the growth phase, but its production rate is subject to variation when the growth rate decreases in the late stage of fed-batch cell cultivation. In some cases, the culture continues to consume glucose and produce lactate at high rates during the period when cell growth has ceased; while in other cases glucose consumption rate on a per cell basis becomes small and lactate production diminishes, or lactate is even consumed [14]. The phenomenon thus suggests that the two types of cultures differ in their trajectories while progressing on the high flux plane.

**Fig 6:**
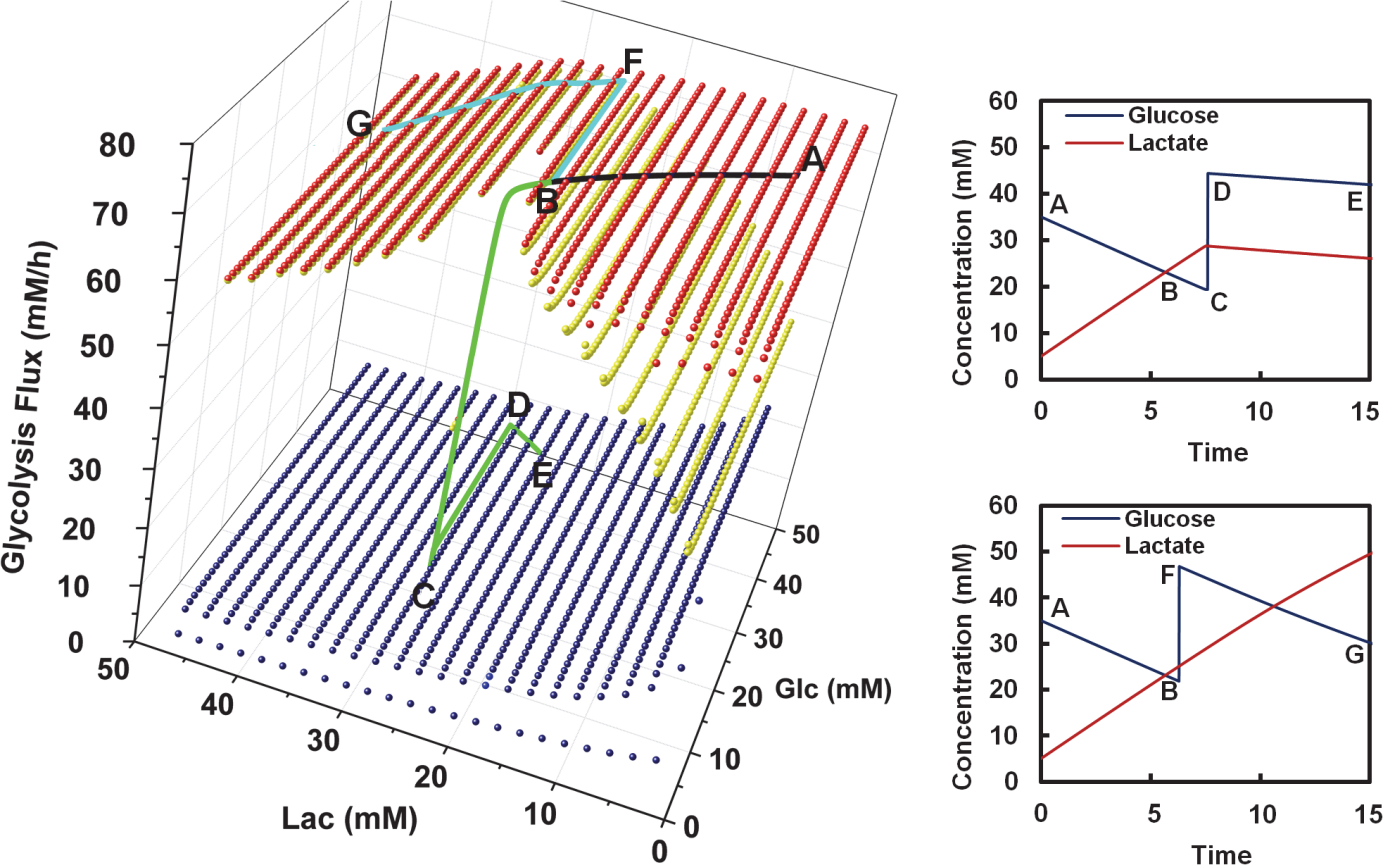
Transient simulations of cultures with and without metabolic shift. At t = 0, cells are stationed at a state of high growth rate (high pAKT level) with a high flux state, high glucose and low lactate concentrations (graph on the left hand side of the figure). (A-B) As the culture progresses, growth rate and AKT activity decreases (pAKT levels decrease from 1.00 to 0.17), glucose is consumed and lactate accumulates in the culture such that the trajectory follows the curve A-B in the top plane. (B-C) After a certain time, the culture encounters a section of the top plane that regresses (seen in slow growth phase). The cells have sufficient time to enter the regressed region and shift their metabolism from the high flux state to the low flux state. (C-D-E) Glucose is added to the culture after the cells switch to low flux state. After glucose addition, cells remain at the low flux state and consume lactate. (B-F) Glucose is added before cells encounter the regressed plane. (F-G) No metabolic shift observed. Shown on the right side of the figure are the two graphs, each depicting the metabolic profiles of the above mentioned two cultures as a function of time. Simulation shows that with early glucose feed the cells are able to avoid the regressed region of the top plane and continue their trajectory along the top plane, consuming large amounts of glucose and diverting most of it towards lactate production.

We performed a set of transient simulations to demonstrate different scenarios where cultures may have different metabolic fates (Fig. 6). Cells are initially at a position on the high flux plane of high pAKT (in growth stage) and move along a path with decreasing glucose concentration (due to glucose consumption) and increasing lactate concentration (due to lactate production). As the growth rate decreases, the culture progression is depicted by a line (line AB, Fig. 6) in a surface plot corresponding to pAKT activation of 0.17 (corresponding to later stages of the cell culture, which resembles Fig. 5C). In the first case, the path encounters the receded section of the high flux plane leading to a switch down from the high flux plane to the low flux plane (line BC, Fig. 6). In the other case, while moving along the path (line AB, Fig. 6) glucose concentration is increased due to glucose feeding in fed-batch culture (line BF, Fig. 6). The feeding causes the path to shift and eventually leads to a trajectory that does not encounter the receded plane thus confining the cells to the high flux plane. In the first case, upon switching to a low flux state, an addition of the same amount of glucose to the culture (line CD, Fig. 6) will not cause the culture to switch back to a high flux state. Such a difference in the timing of glucose feeding is not uncommon in industrial manufacturing, as observed in the archived manufacturing data [14, 15] and in laboratory practices. Even cultures under the same conditions are not exact replicas. Feeding of nutrient and glucose to a fed-batch culture typically is either prescribed as fixed time point or responding to a range of the controlled variables. A small difference in the timing of glucose feeding may cause cultures with very similar metabolic behavior to diverge to different outcomes.

### The Effect of Initial Lactate Level on Metabolic Trajectory of CHO Cells in Culture

A recombinant CHO cell line was grown in four fed-batch cultures with initial sodium lactate concentrations of 0, 10, 25 and 40 mM. Osmolarity at inoculation was adjusted to 300 mOsm/kg in all four conditions using appropriate amounts of sodium chloride. The concentration profiles of lactate and growth curves are shown along with the specific lactate production rates (S2 Fig.). Under all four different lactate concentrations, cells were in a high flux state during the growth phase. A shift in the metabolism was observed in the late stage (stationary phase) of cultures with 0 mM and 10 mM initial lactate supplementation. In contrast, the cultures initially at higher lactate levels (25 mM and 40 mM) remained at high flux state even in the late stages with continued lactate production (S2A and S2C Fig.).

Lactate concentration also exerted a significant effect on growth rate, thus the difference in the metabolism of the four cultures may not be directly related to lactate concentration. Nevertheless, the results are consistent with the model prediction shown in Fig. 5 that a switch from the high flux state to the low flux state, while glucose level is moderately high, occurs only within a certain range of extracellular lactate concentrations. At higher levels of lactate than the “switch-permissible” range, as in cases of 25 and 40 mM, cells cannot switch to a low flux state.

The effect of lactate concentration on the metabolic behavior of the culture is also evident from the data reported previously [14]. Glucose and lactate profiles of runs in the top 20% (blue) and bottom 20% (red) with respect to product titer are plotted during the time period of transitioning from high flux to low flux state (S3 Fig.). Data from the same run are connected by lines. All runs traverse from the right hand side of the figure (where glucose is high) towards the left hand side (where glucose is low). The runs in blue had lower lactate concentration in the early (transient) phase and shifted to low flux state in the later stage as indicated by the negative specific lactate production rates. In contrast, those colored in red had higher lactate concentration in the early phase and yet continued in their trajectories on high flux state as shown by the high specific lactate production rate and accumulate lactate to very high levels.

### The Effect of History on Metabolic Shift of CHO cells in Culture

**Fig 7:**
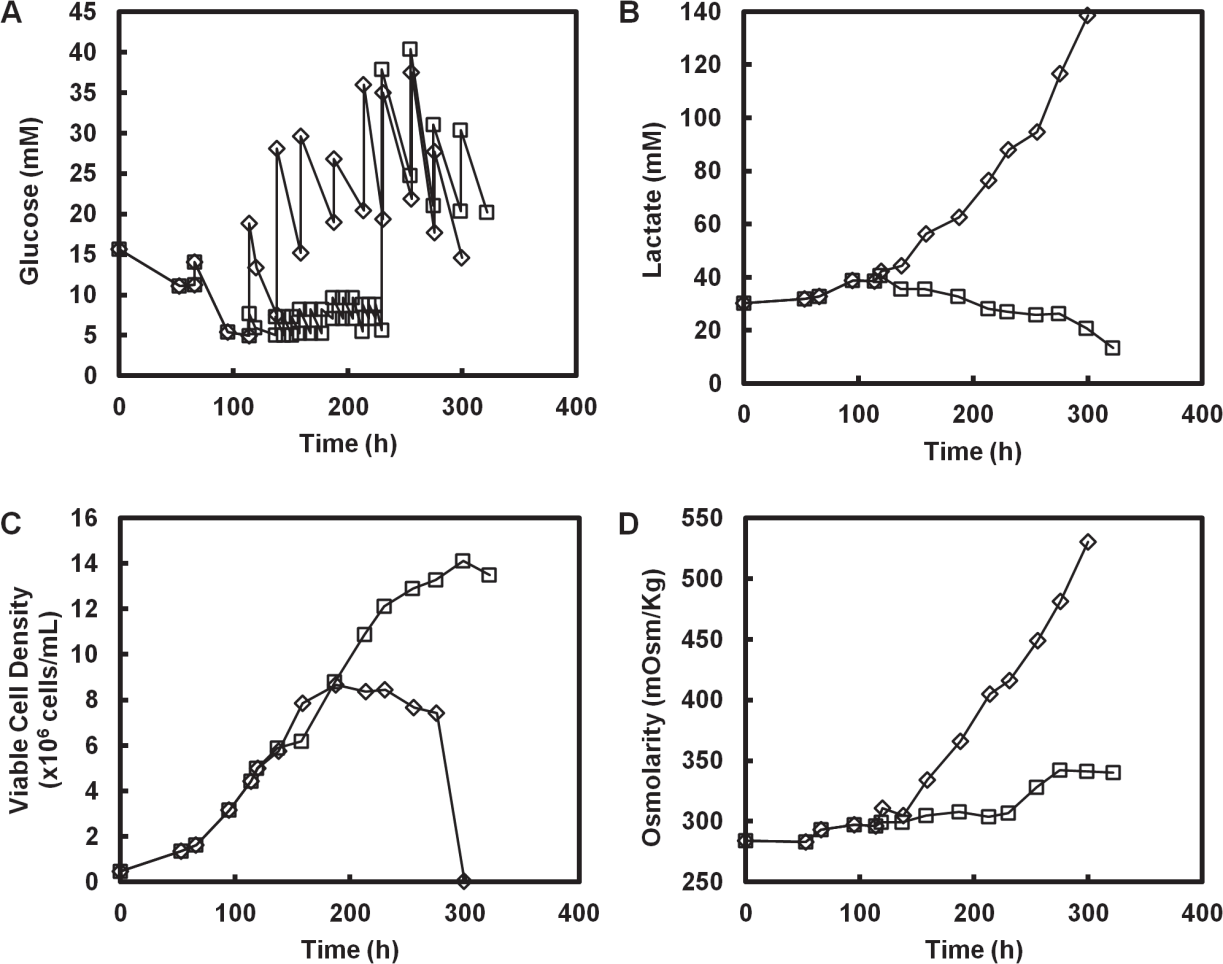
Memory of metabolic state. Two fed-batch cultures of a recombinant CHO cell line were run with different feeding strategies. (◊): Glucose was maintained at high levels throughout the culture and the culture continued to produce lactate. (□): Glucose was maintained at a low concentration for a period of time by intermittent glucose feeding. The cells shifted their metabolism to lactate consumption. Lactate continued to be consumed even after glucose was restored to a high concentration. (A) Glucose (B) Lactate (C) Viable Cell Density (D) Osmolarity.

Cells were next cultivated in two fed-batch conditions (Fig. 7; replicates are reported in S4 and S5 Figs.). Each condition was supplemented with 30 mM of sodium lactate as described in the previous section. The two conditions were treated identically until late exponential growth stage. One culture received glucose feed to maintain glucose at a higher concentration (Fig. 7A, open diamonds); glucose in the other culture was allowed to decrease to a low level and maintained at low levels for a period of time by intermittent glucose spiking (Fig. 7A, open squares). In the first culture, glucose continued to be consumed at a high rate and lactate continued to accumulate even after viable cell concentration decreased. The results for the first culture are the same as those shown in S2 Fig., where at high levels of lactate, a switch to a low glycolytic flux (or alternatively lactate consumption state) at moderately high level of glucose is not possible. This is in contrast to the results of the second culture. By allowing glucose concentration to decrease to a low level, metabolism switched to a low flux state as predicted in Figs. 5 and 6. Upon switching to a low flux state, which in this scenario is characterized by a switch to lactate consumption, the glucose level in the second culture was increased to high levels again at 230h. In this case glucose consumption continued to be low, a clear indication that once a low flux state is achieved cell metabolism can be maintained at a low flux state even with an increase in glucose concentration, unless the glucose concentration is increased to a level beyond the switch-up concentration.

## Discussion

Using a mechanistic model we have shown previously that with the combination of isozymes typically seen in proliferating cells, the glycolysis flux exhibits classical steady state multiplicity [5]. In the bistable region, both high flux and low flux states can exist; at the edges of bistable region, the metabolism may shift-up or shift-down to a different flux state when glucose concentration is changed from the bistable region into regions in which only one steady state is possible. The glucose concentration for shift-down from a high flux state to a low flux state is notably low and outside the physiological range. The model prediction of low shift-down glucose concentration is consistent with reported fed-batch culture studies [8, 9, 36, 37]. In those studies, the glucose concentration was controlled at a very low level in order for the switch to occur, akin to the results of the model prediction.

In many fed-batch cultures, cell metabolism switches from a high flux and lactate production state during the exponential growth to a low flux and low lactate production or lactate consumption state when the growth rate diminishes [10–13]. A prominent difference between the metabolic switch in the late stage of fed-batch culture and the earlier reports of metabolic shift through controlled glucose feeding is the glucose level at the point of metabolic switch. While in the early studies it was necessary to control glucose at very low levels, in later stage fed-batch cultures the glucose concentration can be high at the time of switching.

In recent times much progress has been made in advancing our understanding of the interplay between energy metabolism and growth control, and the oncological transformation. Of particular relevance to cell culture bioprocessing is the influence of lactate levels and growth control on glycolysis [3, 11]. To this end, we incorporated the inhibitory effects of lactate and regulatory role of growth (through AKT signaling) in our mechanistic model of energy metabolism.

Model simulation demonstrated that increasing lactate concentration (in 0–20mM range, Fig. 5) increases the “switch-down” glucose concentration, allowing the switch from high flux to low flux state at a higher level of glucose typically seen in the late stages of culture. However, at a low AKT activation level, the effect of lactate is not monotonic (Fig. 5). At low levels of lactate an increase of its concentration increases the “switch-down” glucose concentration (0–25mM Lac, Fig. 5C), but beyond a certain point the trend is reversed; further increase of its concentration actually decreases the “switch-down” glucose concentration (beyond 25mM Lac, Fig. 5C). There is thus a window of lactate concentration within which the “switch-down” glucose concentration is highest; at lactate concentrations higher or lower than that, the “switch-down” glucose concentration decreases again.

As discussed previously, the bistable behavior in the glycolysis arises from the feedback activation due to two regulatory loops [5]. Loop 1 consists of feedback activation of PFKM by F16BP while loop 2 consists of feed-forward activation of PKM2 by F16BP, activation of PFKM by F26BP and feedback inhibition of PFKFB3 by PEP. The non-monotonic response to lactate concentration is caused by the opposing effects of lactate inhibition on loop 1 and the flux enhancing effect of F26BP and F16BP on both loop 1 and loop 2. This can be seen by plotting the intracellular concentrations of lactate, F16BP and F26BP (S6 Fig.). The three regions corresponding to high flux, low flux and unstable steady states are colored in red, blue and yellow, respectively. The intracellular lactate concentration increases gradually with increasing external lactate concentration which results in increased inhibition of PFK. However, F16BP concentration remains relatively constant over a range of lactate concentrations on the right segment of the high flux plane but then increases sharply as the “switch-down” glucose concentration decreases with increasing lactate concentration. The same trend is seen with F26BP, indicating that the activation effects of F26BP and F16BP begin to overtake the inhibitory effect of lactate at high lactate concentrations. Such changes in the resultant intracellular metabolite concentrations with changing extracellular lactate (and glucose) affect the activity of the two loops (loop 1 and loop 2) either positively or negatively. This can result in one loop being active, both loops being active, or neither of the two loops being active. The region where the high flux state recedes is characterized by the lactate and the glucose concentration ranges (and the resultant intermediate concentrations) which prevent either of the two regulatory loops to be active. Hence, no multiplicity of the states is possible in this range of glucose and lactate, resulting in the possibility of only one state i.e. the low flux state. This allows for a switch in the metabolic state from high flux to the low flux state when the cell’s metabolic trajectory encounters this region.

Such multiple steady states in the glycolysis pathway as reported here have not been studied in detail, except for the oscillatory behavior in yeast cells, pancreatic beta glycolysis and few other tissues [38–45]. The dynamics in glycolysis activity is affected by the isozyme composition and their relative expression levels in the cell [5, 43]. Different cell lines express glycolysis enzymes at somewhat different levels that might affect their metabolic behavior. This may be part of the reason that the propensity to switch to a low flux or lactate consumption state differs among different cell lines. But even for the same cell line, under presumably “identical” culture conditions, the switch from a high flux state to a low flux state is not robust as shown in the compiled historical data [14, 15]. We demonstrated that one possible explanation for this is the subtle difference in the glucose addition time. The complex topology of flux response to glucose and lactate concentration makes the precise control of shift down to low flux state unpredictable.

Various studies have been undertaken to explore the means of facilitating metabolic shift to a low flux state and lactate consumption metabolic state. In this study, we demonstrated that one can direct cell metabolism to a low flux state through control of glucose level at lower concentration range (Fig. 6). Upon successful metabolic shift, even if the glucose level is reset to a higher level, the flux will remain at a low flux state. The experimental data presented in this study provide further evidence to support our model prediction. Others have reported that the switch in the metabolism only happens in cell lines which maintain higher oxidative capacity in the late stages of the cultures [46]. Transcriptome data of recombinant mouse myeloma indicate that the oxidative phosphorylation genes are up-regulated at transcript level under lactate consumption conditions [10]. Supplementation of copper has been reported to enhance the lactate consumption phenotype in late stages of fed-batch cultures [47, 48]. Through transcriptome analysis on copper-supplemented culture, the early growth response protein 1 (EGR1), a gene also related to negative regulation of AKT through PTEN, was shown to be up-regulated in high copper condition [49]. Alternatively, in a separate study, the expression of anti-apoptotic genes E1B-19K and Aven was shown to favor lactate consumption [50].

Recently, there has been increasing interest in employing continuous culture or continuous culture with cell recycle (perfusion culture) for cell cultivation. The rationale is that continuous processes minimize equipment down time and increase overall productivity. Since multiplicity of metabolic state occurs under some conditions, it is likely that those distinctive metabolic states will also lead to different steady states of the culture: at a high flux state, the vast majority of glucose is converted to lactate resulting in a low viable cell concentration at the steady state reached; conversely at a low flux state, little glucose is diverted to lactate, and a high cell concentration is achieved. Published experimental results of continuous culture did suggest that difference in metabolism gave rise to different cell concentrations [51–53].

The mechanistic metabolic model presented above has provided insights into the possible cause of the somewhat erratic behavior of metabolic shift to a low flux and lactate consumption state often seen in cell culture bioprocessing. The experimental evidence supports the notion that the steady state multiplicity is at play, and may possibly be the root cause of this behavior. With a better understanding of the mechanistic cause for the metabolic shift in culture, one can begin to explore the feasibility of controlling the metabolism in a robust manner and ensuring metabolic shift through external medium components such as enzyme inhibitors or through cell engineering methods.

## Supporting Information

S1 Fig Expression levels of glycolytic isozymes across different types of CHO cells. Transcript levels of isozymes of (A) PFKFB, (B) HK, (C) PFK and (D) PK were analyzed using CHO microarrays. CHO cell lines and the culture conditions used for the transcript analysis are listed in S2 Table.
(TIF)

S2 Fig Effect of initial lactate concentration on the metabolic trajectory of cells in fed-batch culture. Four fed-batch cultures of a recombinant CHO cell line were inoculated with different concentrations of initial lactate including 0mM (◊), 10mM (□), 25mM (Δ) and 45mM (×). The metabolic fate of a culture depends on the initial lactate concentration. The cultures that were exposed to higher lactate concentrations had higher specific lactate production rates in the later stages of the cultures. Specific lactate production rates for growth phase were calculated using data between 41–88h whereas those for stationary phase were calculated using data between 160–183h. (A) Lactate (B) Viable Cell Density (C) Specific Lactate Production Rate.
(TIF)

S3 Fig Trajectories of manufacturing runs. Time series data of a number of manufacturing runs of the same process with harvest titers in the top 20% (blue) and bottom 20% (red) are plotted. Data points from the same bioreactor run are connected by lines. All cultures proceed from the right hand side of the figure, where glucose is high, towards the left hand side where glucose is low. Cultures with low lactate concentration in the early stage exhibit metabolic shift to lactate consumption in their later stage, while those that continue to produce lactate at high rate in the late stage are those with higher lactate levels in the early stages of the culture.
(TIF)

S4 Fig Growth and metabolic kinetics of replicate culture for Fig. 7. The seed culture was same as that used for Fig. 7. The production medium and the feed medium used were the same. The feeding scheme used was same as that used for the corresponding conditions in Fig. 7. (◊): Glucose was maintained at high levels throughout the culture and the culture continued to produce lactate. (□): Glucose was maintained at a low concentration for a period of time by intermittent glucose feeding. Metabolic shift to lactate consumption was seen in the low glucose culture and continued after reverting to high glucose level. (A) Glucose (B) Lactate (C) Viable Cell Density (D) Osmolarity.
(TIF)

S5 Fig Growth and metabolic kinetics of replicate culture for Fig. 7. The seed cultures used were prepared using cells from a different frozen vial of the same cell bank at a different point in time, as compared to that used in Fig. 7. The production and feed medium used were same but were from a different lot. The feeding scheme was the same except minor differences in the feeding time of glucose because of the small differences in the glucose consumption rate, as compared to the experiments shown in Fig. 7. (◊): Glucose was maintained at high levels throughout the culture and the culture continued to produce lactate. (□): Glucose was maintained at low concentrations for a period of time by intermittent glucose feeding. Metabolic shift to lactate consumption was seen in the low glucose culture and continued after reverting to high glucose levels. (A) Glucose (B) Lactate (C) Viable Cell Density (D) Osmolarity.
(TIF)

S6 Fig Concentration profile of (A) F16BP (B) Intracellular lactate (C) F26BP. Intracellular lactate concentration increases monotonically with increasing extracellular lactate. F16BP and F26BP concentrations are relatively constant at lower extracellular lactate concentrations, but increase sharply at higher extracellular lactate levels. pAKT activity was set constant at 0.25.
(TIF)

S1 Table List of abbreviations. (DOCX)

S2 Table List of CHO cell lines used for microarray gene expression analysis. (DOCX)

S3 Table Fixed parameter values of the model. (DOCX)

S4 Table Bounds of enzyme activity within which bistability is observed. (DOCX)

S1 File Supplementary materials. The file contains description of mathematical model rate equations, differential equations and stability analysis.
(DOC)

## References

[1] Warburg O. On the origin of cancer cells. Science. 1956;123(3191):309–14. 13298683 10.1126/science.123.3191.309

[2] Cairns RA, Harris IS, Mak TW. Regulation of cancer cell metabolism. Nat Rev Cancer. 2011;11(2):85–95. 10.1038/nrc2981 21258394

[3] Mulukutla BC, Khan S, Lange A, Hu WS. Glucose metabolism in mammalian cell culture: new insights for tweaking vintage pathways. Trends Biotechnol. 2010;28(9):476–84. 10.1016/j.tibtech.2010.06.005 20691487

[4] Levine AJ, Puzio-Kuter AM. The control of the metabolic switch in cancers by oncogenes and tumor suppressor genes. Science. 2010;330(6009):1340–4. 10.1126/science.1193494 21127244

[5] Mulukutla BC, Yongky A, Daoutidis P, Hu WS. Bistability in glycolysis pathway as a physiological switch in energy metabolism. PloS one. 2014;9(6):e98756 10.1371/journal.pone.0098756 24911170 PMC4049617

[6] Folmes CD, Dzeja PP, Nelson TJ, Terzic A. Metabolic plasticity in stem cell homeostasis and differentiation. Cell Stem Cell. 2012;11(5):596–606. 10.1016/j.stem.2012.10.002 23122287 PMC3593051

[7] Folmes CD, Nelson TJ, Martinez-Fernandez A, Arrell DK, Lindor JZ, Dzeja PP, et al Somatic oxidative bioenergetics transitions into pluripotency-dependent glycolysis to facilitate nuclear reprogramming. Cell Metab. 2011;14(2):264–71. 10.1016/j.cmet.2011.06.011 21803296 PMC3156138

[8] Zhou W, Rehm J, Hu WS. High viable cell concentration fed-batch cultures of hybridoma cells through on-line nutrient feeding. Biotechnology and bioengineering. 1995;46(6):579–87. 18623353 10.1002/bit.260460611

[9] Zhou W, Rehm J, Europa A, Hu WS. Alteration of mammalian cell metabolism by dynamic nutrient feeding. Cytotechnology. 1997;24(2):99–108. 10.1023/A:1007945826228 22358650 PMC3449580

[10] Mulukutla BC, Gramer M, Hu WS. On metabolic shift to lactate consumption in fed-batch culture of mammalian cells. Metabolic engineering. 2012;14(2):138–49. 10.1016/j.ymben.2011.12.006 22244936

[11] Young JD. Metabolic flux rewiring in mammalian cell cultures. Current opinion in biotechnology. 2013;24(6):1108–15. 10.1016/j.copbio.2013.04.016 23726154 PMC3775942

[12] Templeton N, Dean J, Reddy P, Young JD. Peak antibody production is associated with increased oxidative metabolism in an industrially relevant fed-batch CHO cell culture. Biotechnology and bioengineering. 2013;110(7):2013–24. 10.1002/bit.24858 23381838

[13] Ma N, Ellet J, Okediadi C, Hermes P, McCormick E, Casnocha S. A single nutrient feed supports both chemically defined NS0 and CHO fed-batch processes: Improved productivity and lactate metabolism. Biotechnology progress. 2009;25(5):1353–63. 10.1002/btpr.238 19637321

[14] Le H, Kabbur S, Pollastrini L, Sun Z, Mills K, Johnson K, et al Multivariate analysis of cell culture bioprocess data—lactate consumption as process indicator. J Biotechnol. 2012;162(2–3):210–23. 10.1016/j.jbiotec.2012.09.018 22974585

[15] Charaniya S, Le H, Rangwala H, Mills K, Johnson K, Karypis G, et al Mining manufacturing data for discovery of high productivity process characteristics. J Biotechnol. 2010;147(3–4):186–97. 10.1016/j.jbiotec.2010.02.022 20416347

[16] Vora S, Oskam R, Staal GE. Isoenzymes of phosphofructokinase in the rat. Demonstration of the three non-identical subunits by biochemical, immunochemical and kinetic studies. The Biochemical journal. 1985;229(2):333–41. 2931076 10.1042/bj2290333 PMC1145065

[17] Reinhart GD, Lardy HA. Rat liver phosphofructokinase: kinetic activity under near-physiological conditions. Biochemistry. 1980;19(7):1477–84. 6446316 10.1021/bi00548a034

[18] Van Schaftingen E, Jett MF, Hue L, Hers HG. Control of liver 6-phosphofructokinase by fructose 2,6-bisphosphate and other effectors. Proc Natl Acad Sci U S A. 1981;78(6):3483–6. 6455662 10.1073/pnas.78.6.3483 PMC319593

[19] Yaney GC, Schultz V, Cunningham BA, Dunaway GA, Corkey BE, Tornheim K. Phosphofructokinase isozymes in pancreatic islets and clonal beta-cells (INS-1). Diabetes. 1995;44(11):1285–9. 7589825 10.2337/diab.44.11.1285

[20] Yamada K, Noguchi T. Nutrient and hormonal regulation of pyruvate kinase gene expression. The Biochemical journal. 1999;337 (Pt 1):1–11. 9854017 PMC1219928

[21] Kretschmer M, Schellenberger W, Hofmann E. Quasi-stationary concentrations of fructose-2,6-bisphosphate in the phosphofructokinase-2/fructose-2,6-bisphosphatase cycle. Biochem Biophys Res Commun. 1985;131(2):899–904. 2996530 10.1016/0006-291x(85)91324-5

[22] Manes NP, El-Maghrabi MR. The kinase activity of human brain 6-phosphofructo-2-kinase/fructose-2,6-bisphosphatase is regulated via inhibition by phosphoenolpyruvate. Arch Biochem Biophys. 2005;438(2):125–36. 15896703 10.1016/j.abb.2005.04.011

[23] Tominaga N, Tsujikawa T, Minami Y, Wu RF, Watanabe F, Sakakibara R, et al Effect of replacement of the amino and the carboxyl termini of rat testis fructose 6-phosphate, 2-kinase:fructose 2,6-bisphosphatase with those of the liver and heart isozymes. Arch Biochem Biophys. 1997;347(2):275–81. 9367536 10.1006/abbi.1997.0346

[24] Ros S, Schulze A. Balancing glycolytic flux: the role of 6-phosphofructo-2-kinase/fructose 2,6-bisphosphatases in cancer metabolism. Cancer Metab. 2013;1(1):8 10.1186/2049-3002-1-8 24280138 PMC4178209

[25] Costa Leite T, Da Silva D, Guimaraes Coelho R, Zancan P, Sola-Penna M. Lactate favours the dissociation of skeletal muscle 6-phosphofructo-1-kinase tetramers down-regulating the enzyme and muscle glycolysis. The Biochemical journal. 2007;408(1):123–30. 17666012 10.1042/BJ20070687 PMC2049071

[26] Barthel A, Okino ST, Liao J, Nakatani K, Li J, Whitlock JP Jr, et al Regulation of GLUT1 gene transcription by the serine/threonine kinase Akt1. The Journal of biological chemistry. 1999;274(29):20281–6. 10400647 10.1074/jbc.274.29.20281

[27] Robey RB, Hay N. Mitochondrial hexokinases, novel mediators of the antiapoptotic effects of growth factors and Akt. Oncogene. 2006;25(34):4683–96. 16892082 10.1038/sj.onc.1209595

[28] Majewski N, Nogueira V, Bhaskar P, Coy PE, Skeen JE, Gottlob K, et al Hexokinase-mitochondria interaction mediated by Akt is required to inhibit apoptosis in the presence or absence of Bax and Bak. Mol Cell. 2004;16(5):819–30. 15574336 10.1016/j.molcel.2004.11.014

[29] Novellasdemunt L, Tato I, Navarro-Sabate A, Ruiz-Meana M, Mendez-Lucas A, Perales JC, et al Akt-dependent activation of the heart 6-phosphofructo-2-kinase/fructose-2,6-bisphosphatase (PFKFB2) isoenzyme by amino acids. The Journal of biological chemistry. 2013;288(15):10640–51. 10.1074/jbc.M113.455998 23457334 PMC3624444

[30] Duran J, Obach M, Navarro-Sabate A, Manzano A, Gomez M, Rosa JL, et al Pfkfb3 is transcriptionally upregulated in diabetic mouse liver through proliferative signals. The FEBS journal. 2009;276(16):4555–68. 10.1111/j.1742-4658.2009.07161.x 19645723

[31] Segel IH. Enzyme kinetics: behavior and analysis of rapid equilibrium and steady state enzyme systems New York: Wiley; 1975.

[32] Monod J, Wyman J, Changeux JP. On the Nature of Allosteric Transitions: A Plausible Model. J Mol Biol. 1965;12:88–118. 14343300 10.1016/s0022-2836(65)80285-6

[33] Mosca E, Barcella M, Alfieri R, Bevilacqua A, Canti G, Milanesi L. Systems biology of the metabolic network regulated by the Akt pathway. Biotechnol Adv. 2012;30(1):131–41. 10.1016/j.biotechadv.2011.08.004 21856401

[34] Mosca E, Alfieri R, Maj C, Bevilacqua A, Canti G, Milanesi L. Computational modeling of the metabolic States regulated by the kinase akt. Front Physiol. 2012;3:418 10.3389/fphys.2012.00418 23181020 PMC3502886

[35] Leite TC, Coelho RG, Da Silva D, Coelho WS, Marinho-Carvalho MM, Sola-Penna M. Lactate downregulates the glycolytic enzymes hexokinase and phosphofructokinase in diverse tissues from mice. FEBS letters. 2011;585(1):92–8. 10.1016/j.febslet.2010.11.009 21074528

[36] Gambhir A, Europa AF, Hu WS. Alteration of cellular metabolism by consecutive fed-batch cultures of mammalian cells. J Biosci Bioeng. 1999;87(6):805–10. 16232558 10.1016/s1389-1723(99)80157-1

[37] Gagnon M, Hiller G, Luan YT, Kittredge A, DeFelice J, Drapeau D. High-end pH-controlled delivery of glucose effectively suppresses lactate accumulation in CHO fed-batch cultures. Biotechnology and bioengineering. 2011;108(6):1328–37. 10.1002/bit.23072 21328318

[38] Gustavsson AK, van Niekerk DD, Adiels CB, du Preez FB, Goksor M, Snoep JL. Sustained glycolytic oscillations in individual isolated yeast cells. The FEBS journal. 2012;279(16):2837–47. 10.1111/j.1742-4658.2012.08639.x 22607453

[39] du Preez FB, van Niekerk DD, Kooi B, Rohwer JM, Snoep JL. From steady-state to synchronized yeast glycolytic oscillations I: model construction. The FEBS journal. 2012;279(16):2810–22. 10.1111/j.1742-4658.2012.08665.x 22712534

[40] du Preez FB, van Niekerk DD, Snoep JL. From steady-state to synchronized yeast glycolytic oscillations II: model validation. The FEBS journal. 2012;279(16):2823–36. 10.1111/j.1742-4658.2012.08658.x 22686585

[41] Gustavsson AK, van Niekerk DD, Adiels CB, Kooi B, Goksor M, Snoep JL. Allosteric regulation of phosphofructokinase controls the emergence of glycolytic oscillations in isolated yeast cells. The FEBS journal. 2014;281(12):2784–93. 10.1111/febs.12820 24751218

[42] Sel'kov EE, Shevelev EL. [Oscillations and trigger phenomena in fructose-2,6-bis-phosphate metabolism. A mathematical model]. Biofizika. 1987;32(2):242–7. 3034335

[43] Westermark PO, Lansner A. A model of phosphofructokinase and glycolytic oscillations in the pancreatic beta-cell. Biophysical journal. 2003;85(1):126–39. 12829470 10.1016/S0006-3495(03)74460-9 PMC1303071

[44] Rizzi M, Baltes M, Theobald U, Reuss M. In vivo analysis of metabolic dynamics in Saccharomyces cerevisiae: II. Mathematical model. Biotechnology and bioengineering. 1997;55(4):592–608. 18636570 10.1002/(SICI)1097-0290(19970820)55:4<592::AID-BIT2>3.0.CO;2-C

[45] Smallbone K, Messiha HL, Carroll KM, Winder CL, Malys N, Dunn WB, et al A model of yeast glycolysis based on a consistent kinetic characterisation of all its enzymes. FEBS letters. 2013;587(17):2832–41. 10.1016/j.febslet.2013.06.043 23831062 PMC3764422

[46] Zagari F, Jordan M, Stettler M, Broly H, Wurm FM. Lactate metabolism shift in CHO cell culture: the role of mitochondrial oxidative activity. New biotechnology. 2013;30(2):238–45. 10.1016/j.nbt.2012.05.021 22683938

[47] Luo J, Vijayasankaran N, Autsen J, Santuray R, Hudson T, Amanullah A, et al Comparative metabolite analysis to understand lactate metabolism shift in Chinese hamster ovary cell culture process. Biotechnology and bioengineering. 2012;109(1):146–56. 10.1002/bit.23291 21964570

[48] Qian Y, Khattak SF, Xing Z, He A, Kayne PS, Qian NX, et al Cell culture and gene transcription effects of copper sulfate on Chinese hamster ovary cells. Biotechnology progress. 2011;27(4):1190–4. 10.1002/btpr.630 21595052

[49] Yuk IH, Zhang JD, Ebeling M, Berrera M, Gomez N, Werz S, et al Effects of copper on CHO cells: insights from gene expression analyses. Biotechnology progress. 2014;30(2):429–42. 10.1002/btpr.1868 24403277

[50] Dorai H, Kyung YS, Ellis D, Kinney C, Lin C, Jan D, et al Expression of anti-apoptosis genes alters lactate metabolism of Chinese Hamster Ovary cells in culture. Biotechnology and bioengineering. 2009;103(3):592–608. 10.1002/bit.22269 19241388

[51] Europa AF, Gambhir A, Fu PC, Hu WS. Multiple steady states with distinct cellular metabolism in continuous culture of mammalian cells. Biotechnology and bioengineering. 2000;67(1):25–34. 10581433 10.1002/(sici)1097-0290(20000105)67:1<25::aid-bit4>3.0.co;2-k

[52] Gambhir A, Korke R, Lee J, Fu PC, Europa A, Hu WS. Analysis of cellular metabolism of hybridoma cells at distinct physiological states. J Biosci Bioeng. 2003;95(4):317–27. 16233414 10.1016/s1389-1723(03)80062-2

[53] Gambhir A, Zhang C, Europa A, Hu WS. Analysis of the use of fortified medium in continuous culture of mammalian cells. Cytotechnology. 1999;31(3):243–54. 10.1023/A:1008026613975 19003148 PMC3449539

