# Supplementary figures and images for: Multiplicity of Steady States in Glycolysis and Shift of Metabolic State in Cultured Mammalian Cells
Source: PLoS One. 2015 Mar 25;10(3):e0121561. doi: 10.1371/journal.pone.0121561 (PMC4373774; doi:10.1371/journal.pone.0121561)

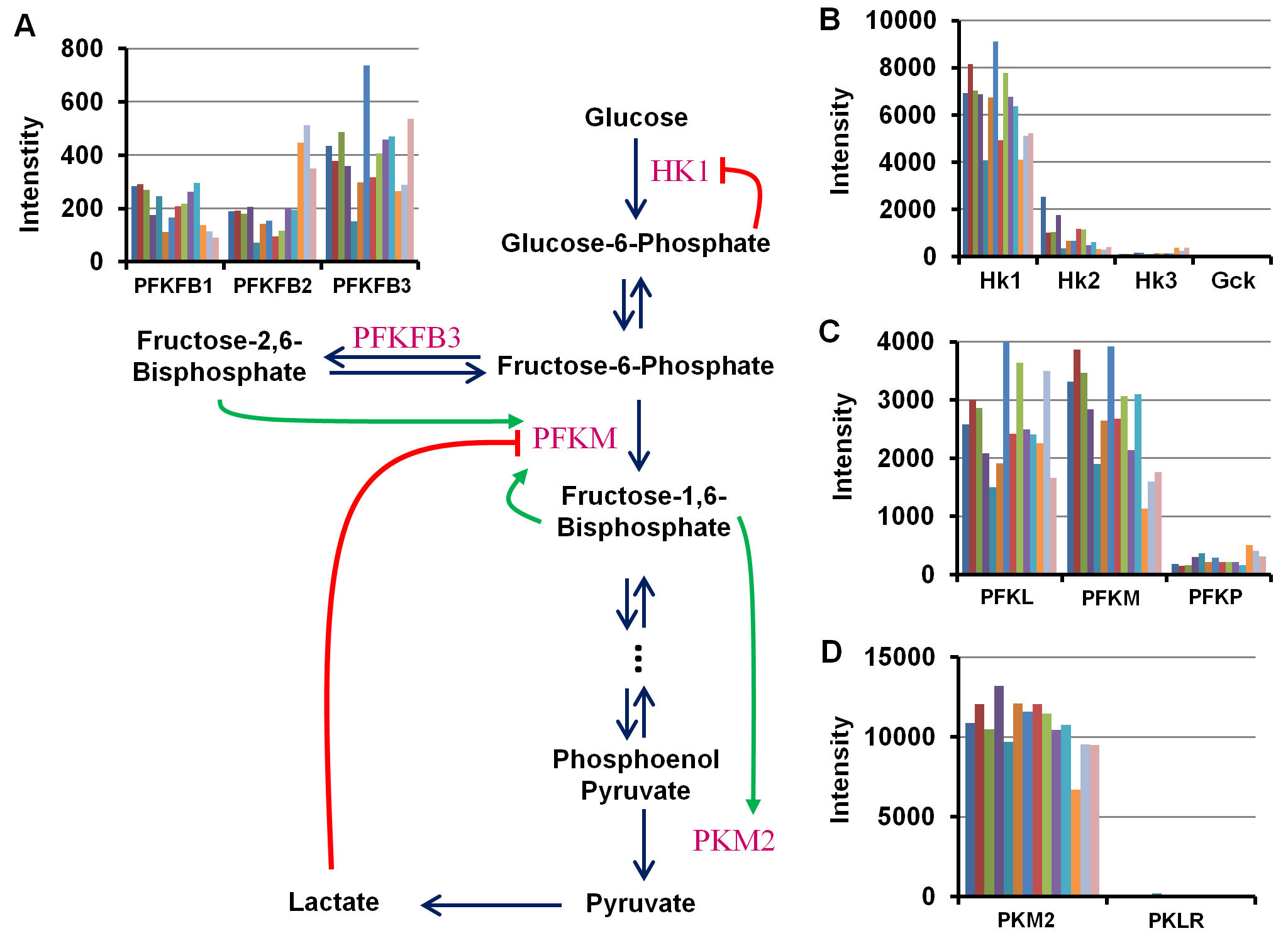

Supplement: S1 Fig — Transcript levels of isozymes of (A) PFKFB, (B) HK, (C) PFK and (D) PK were analyzed using CHO microarrays. CHO cell lines and the culture conditions used for the transcript analysis are listed in S2 Table. (TIF) [file pone.0121561.s001.tif]

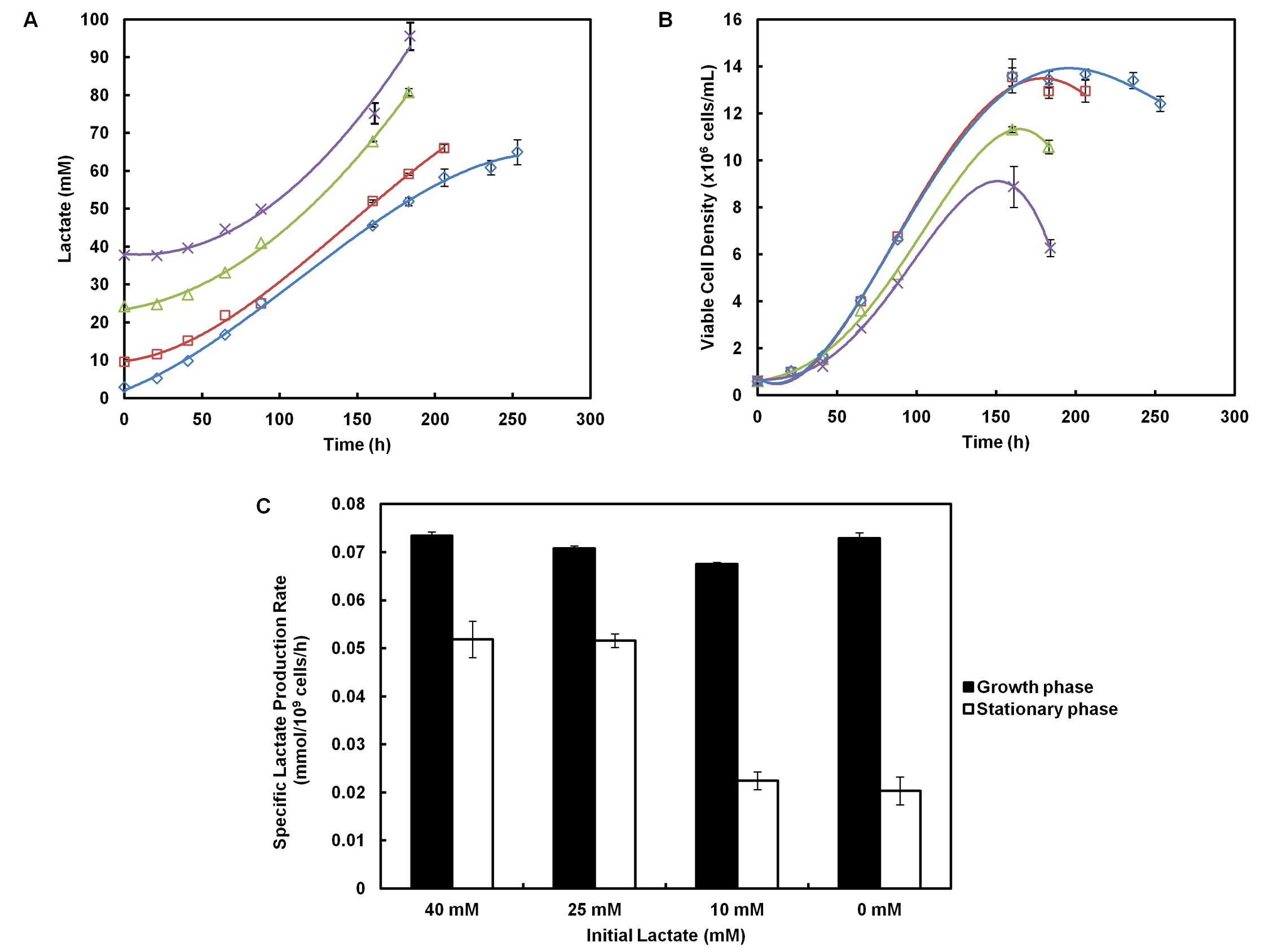

Supplement: S2 Fig — Four fed-batch cultures of a recombinant CHO cell line were inoculated with different concentrations of initial lactate including 0mM (◊), 10mM (□), 25mM (Δ) and 45mM (×). The metabolic fate of a culture depends on the initial lactate concentration. The cultures that were exposed to higher lactate concentrations had higher specific lactate production rates in the later stages of the cultures. Specific lactate production rates for growth phase were calculated using data between 41–88h whereas those for stationary phase were calculated using data between 160–183h. (A) Lactate (B) Viable Cell Density (C) Specific Lactate Production Rate. (TIF) [file pone.0121561.s002.tif]

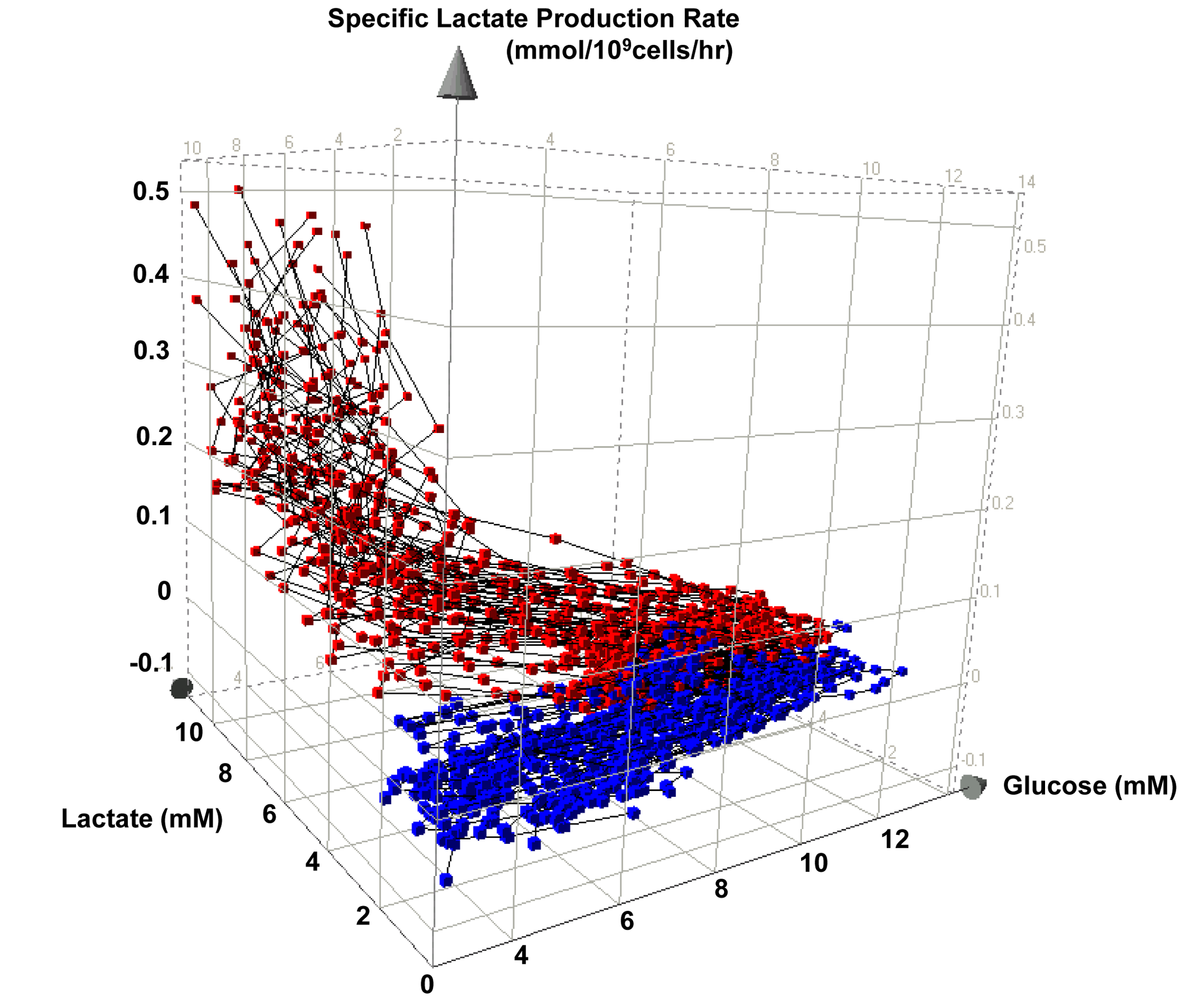

Supplement: S3 Fig — Time series data of a number of manufacturing runs of the same process with harvest titers in the top 20% (blue) and bottom 20% (red) are plotted. Data points from the same bioreactor run are connected by lines. All cultures proceed from the right hand side of the figure, where glucose is high, towards the left hand side where glucose is low. Cultures with low lactate concentration in the early stage exhibit metabolic shift to lactate consumption in their later stage, while those that continue to produce lactate at high rate in the late stage are those with higher lactate levels in the early stages of the culture. (TIF) [file pone.0121561.s003.tif]

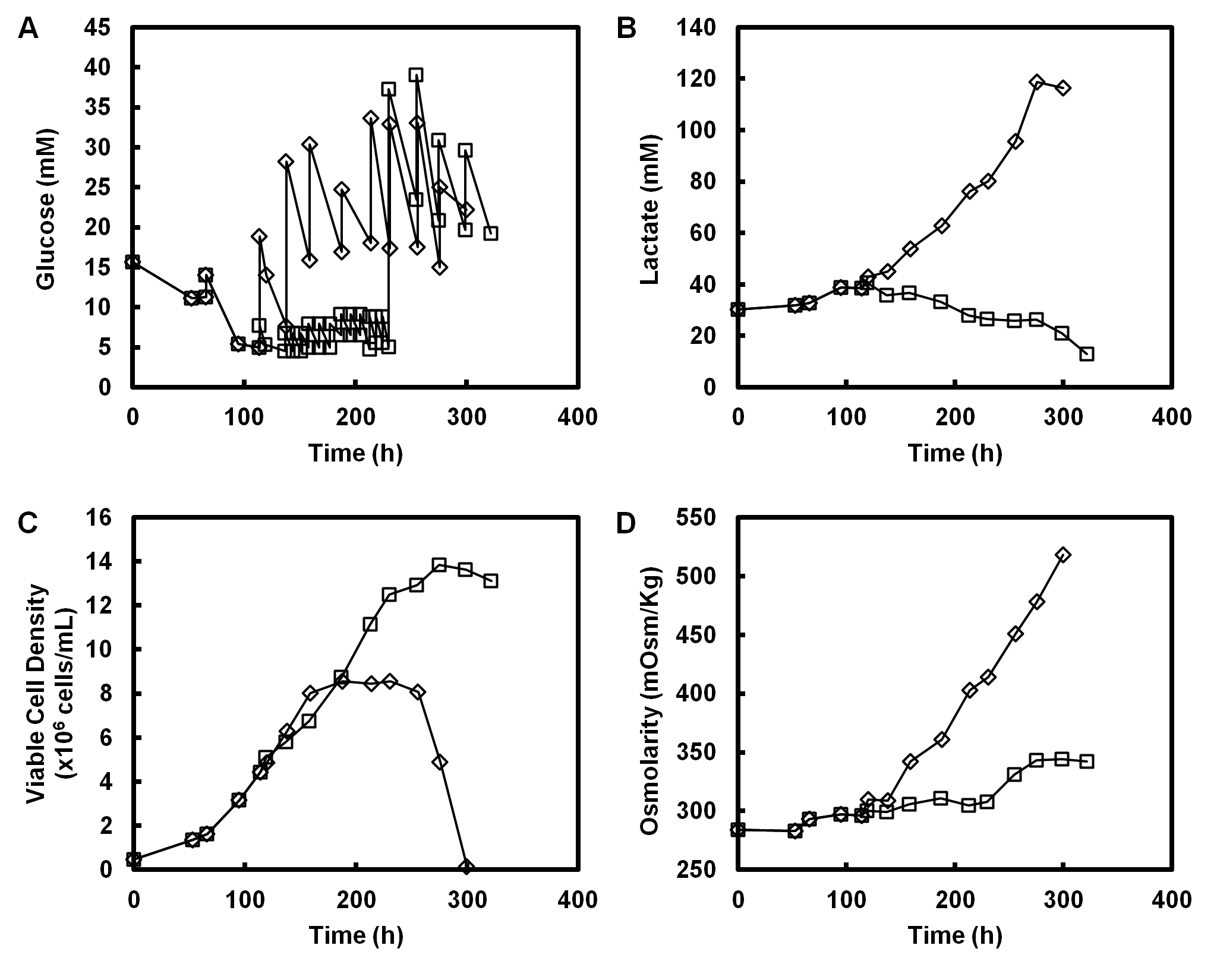

Supplement: S4 Fig — The seed culture was same as that used for Fig. 7. The production medium and the feed medium used were the same. The feeding scheme used was same as that used for the corresponding conditions in Fig. 7. (◊): Glucose was maintained at high levels throughout the culture and the culture continued to produce lactate. (□): Glucose was maintained at a low concentration for a period of time by intermittent glucose feeding. Metabolic shift to lactate consumption was seen in the low glucose culture and continued after reverting to high glucose level. (A) Glucose (B) Lactate (C) Viable Cell Density (D) Osmolarity. (TIF) [file pone.0121561.s004.tif]

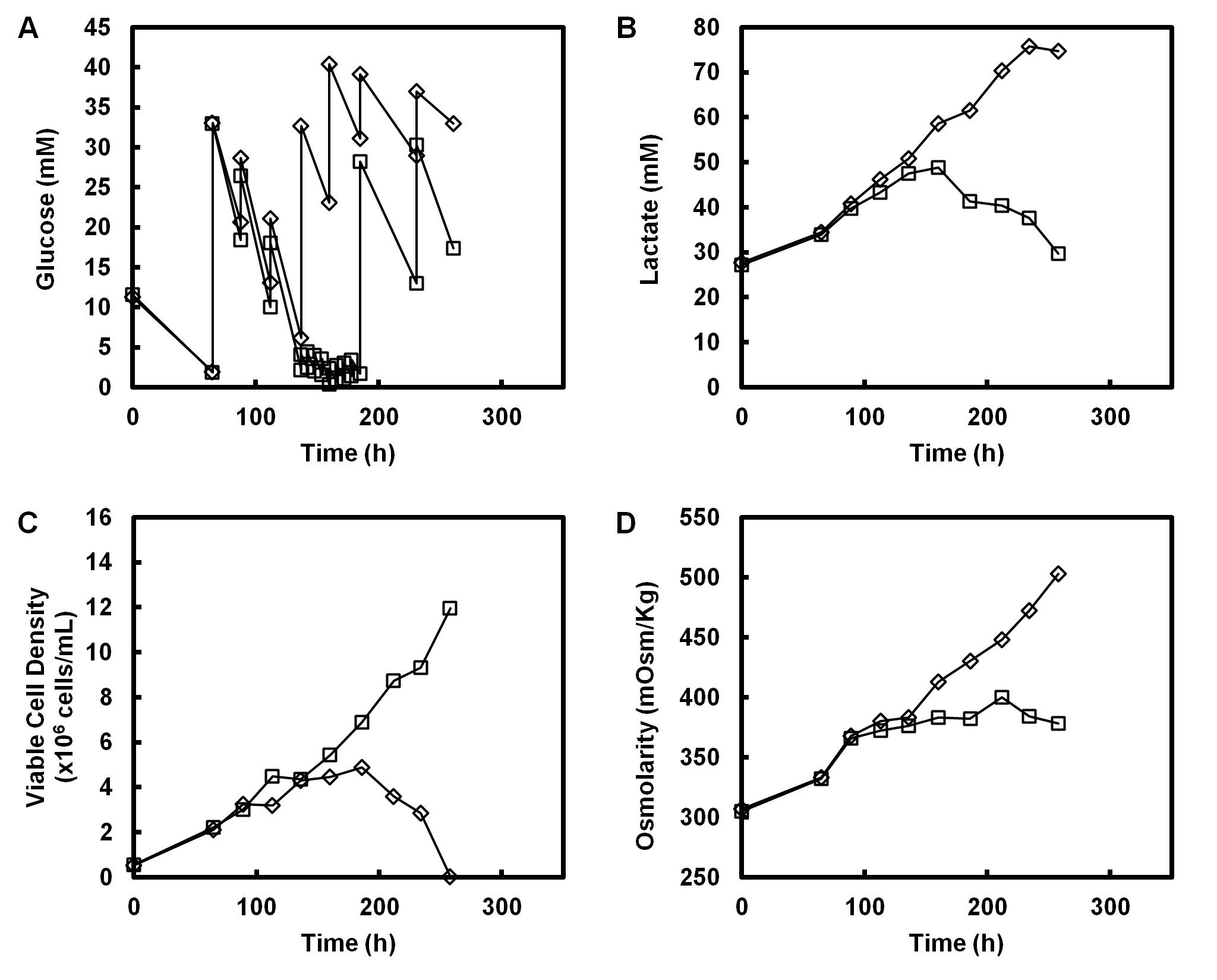

Supplement: S5 Fig — The seed cultures used were prepared using cells from a different frozen vial of the same cell bank at a different point in time, as compared to that used in Fig. 7. The production and feed medium used were same but were from a different lot. The feeding scheme was the same except minor differences in the feeding time of glucose because of the small differences in the glucose consumption rate, as compared to the experiments shown in Fig. 7. (◊): Glucose was maintained at high levels throughout the culture and the culture continued to produce lactate. (□): Glucose was maintained at low concentrations for a period of time by intermittent glucose feeding. Metabolic shift to lactate consumption was seen in the low glucose culture and continued after reverting to high glucose levels. (A) Glucose (B) Lactate (C) Viable Cell Density (D) Osmolarity. (TIF) [file pone.0121561.s005.tif]

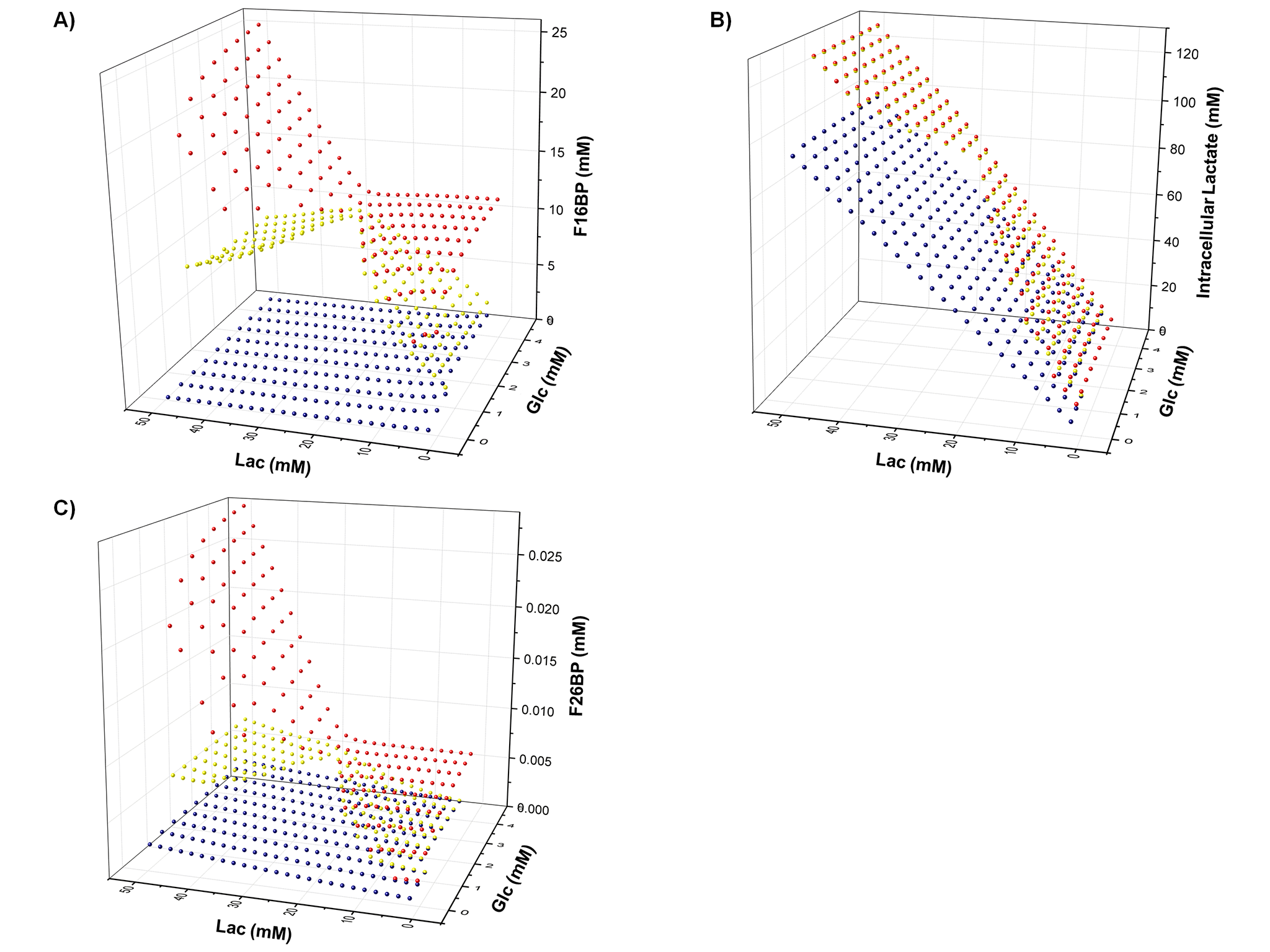

Supplement: S6 Fig — Intracellular lactate concentration increases monotonically with increasing extracellular lactate. F16BP and F26BP concentrations are relatively constant at lower extracellular lactate concentrations, but increase sharply at higher extracellular lactate levels. pAKT activity was set constant at 0.25. (TIF) [file pone.0121561.s006.tif]
