# Supplementary material for: Multiplicity of Steady States in Glycolysis and Shift of Metabolic State in Cultured Mammalian Cells
Source: PLoS One. 2015 Mar 25;10(3):e0121561. doi: 10.1371/journal.pone.0121561 (PMC4373774; doi:10.1371/journal.pone.0121561)
Supplement: S1 Table — (DOCX) [file pone.0121561.s007.docx]

**S1 Table.** List of abbreviations

| ***Abbreviation*** | ***Description*** |
| --- | --- |
| GLC | Glucose |
| G6P | Glucose 6-Phospate |
| F6P | Fructose 6-Phosphate |
| F16BP | Fructose 1,6-Bisphosphate |
| F26BP | Fructose 2,6-Bisphosphate |
| DHAP | Dihydroxyacetone Phosphate |
| G3P | Glyceraldehyde 3-Phosphate |
| 13BPG | 1,3-Bisphosphoglycerate |
| 3PG | 3-Phosphoglycerate |
| 2PG | 2-Phosphoglycerate |
| PEP | Phosphoenolpyruvate |
| Pyr | Pyruvate |
| Lac | Lactate |
| OAA | Oxaloacetate |
| Mal | Malate |
| αKG | alpha-ketoglutarate |
| Ala | Alanine |
| Glu | Glutamate |
| Asp | Aspartate |
| Gln | Glutamine |
| NH3 | Ammonia |
| CO2 | Carbon dioxide |
| NAD+ | Oxidized Nicotinamide Adenine Dinucleotide |
| NADH | Reduced Nicotinamide Adenine Dinucleotide |
| NADP+ | Oxidized Nicotinamide Adenine Dinucleotide Phosphate |
| NAPDH | Reduced Nicotinamide Adenine Dinucleotide Phosphate |
| 6PG | 6-Phosphogluconate |
| E4P | Erythrose 4-Phosphate |
| S7P | Sedulose 7-Phosphate |
| R5P | Ribose 5-Phosphate |
| Ru5P | Ribulose 5-Phosphate |
| PRPP | Phosphoribosyl Pyrophosphate |
| HK | Hexokinase |
| GPI | Glucose Phosphate Isomerase |
| PFK | Phosphofructokinase |
| PFKFB | 6-Phosphofructo-2-Kinase/Fructose 2,6-Bisphosphatase |
| ALDO | Aldolase |
| TPI | Triose Phosphate Isomerase |
| GAPDH | Glyceraldehyde 3-Phosphate Dehydrogenase |
| PGK | Phosphoglycerate Kinase |
| PGM | Phosphoglycerate Mutase |
| ENO | Enolase |
| PK | Pyruvate Kinase |
| LDH | Lactate Dehydrogenase |
| G6PD | Glucose 6-Phosphate Dehydrogenase |
| 6PGD | 6-Phosphogluconate Dehydrogenase |
| RPE | Ribulose Phosphate Epimerase |
| RPI | Ribose Phosphate Isomerase |
| TK | Transketolase |
| TA | Transaldolase |
| PDHC | Pyruvate Dehydrogenase Complex |
| CS | Citrate Synthase |
| ACON | Aconitase |
| IDH | Isocitrate Dehydrogenase |
| AKGD | alpha-ketoglutarate Dehydrogenase |
| SCOAS | Succinyl-CoA Synthetase |
| SDH | Succinate Dehydrogenase |
| FUM | Fumarase |
| MDH | Malate Dehydrogenase |
| GOT | Glutamate Oxaloacetate Transaminase |
| AKGMAL | alpha-ketoglutarate – Malate shuttle |
| ASPGLU | Aspartate – Glutamate shuttle |
| GLUT | Glucose Transporter |
| PYRH | Pyruvate shuttle |
| GLUH | Glutamate shuttle |
| CITMAL | Citrate – Malate shuttle |
| MALPi | Malate – Phosphate shuttle |
| PC | Pyruvate Carboxylase |
| CMALIC | Cytosolic Malic Enyzme |
| MMALIC | Mitochondrial Malic Enyzme |
| GPT1 | Glutamate Alanine Transaminase |
| GLS | Glutaminase |
| GDH | Glutamate Dehydrogenase |
| CLY | ATP-Citrate Lyase |
