# Supplementary material for: Multiplicity of Steady States in Glycolysis and Shift of Metabolic State in Cultured Mammalian Cells
Source: PLoS One. 2015 Mar 25;10(3):e0121561. doi: 10.1371/journal.pone.0121561 (PMC4373774; doi:10.1371/journal.pone.0121561)
Supplement: S2 Table — (DOCX) [file pone.0121561.s008.docx]

**S2 Table.** List of CHO cell lines used for microarray gene expression analysis

| ***Cell Line*** | ***Description (mRNA Source)*** |
| --- | --- |
| 1 | Parental CHO-K1 |
| 2 | Parental DG44   - Exponential growth phase, adherent culture - Serum containing medium |
| 3 | Parental DXB11 |
| 4 | Recombinant DG44 (IgG TNF-alpha fusion protein producer)   - Exponential growth phase, suspension culture - Serum free medium |
| 5 | Recombinant DG44 (IgG)   - Exponential growth phase, suspension culture - Serum free medium |
| 6 | Recombinant DG44 (IgG)   - Late phase, suspension culture - Serum free medium |
| 7 | Recombinant DG44 (IgG)   - Late phase, 2mM butyrate for 24 hr - Serum free medium |
| 8 | DXB11-derived recombinant IgG producer 1 |
| 9 | DXB11-derived recombinant IgG producer 1   - 20 nM MTX treatment |
| 10 | DXB11-derived recombinant DHFR |
| 11 | DXB11-derived recombinant DHFR   - 20 nM MTX treatment |
| 12 | DXB11-derived recombinant IgG producer 2 |
| 13 | DXB11-derived recombinant IgG producer 2   - 20 nM MTX treatment |
| 14 | DXB11-derived recombinant IgG TNF-alpha fusion protein producer |
