# Supplementary material for: Multiplicity of Steady States in Glycolysis and Shift of Metabolic State in Cultured Mammalian Cells
Source: PLoS One. 2015 Mar 25;10(3):e0121561. doi: 10.1371/journal.pone.0121561 (PMC4373774; doi:10.1371/journal.pone.0121561)
Supplement: S3 Table — (DOCX) [file pone.0121561.s009.docx]

**S3 Table.** Fixed parameter values of the model

| **Parameter Symbol** | **Parameter Description** | **Value** | **Units** |
| --- | --- | --- | --- |
| Ccadp | Cytosolic adenine diphosphate nucleotide concentration | 0.54 | mM |
| Cmadp | Mitochondrial adenine diphosphate nucleotide concentration | 0.1 | mM |
| Ccatp | Cytosolic adenine triphosphate nucleotide concentration | 0.31 | mM |
| Cmatp | Mitochondrial adenine triphosphate nucleotide concentration | 0.1 | mM |
| Ccamp | Cytosolic adenine monophosphate nucleotide concentration | 0.03 | mM |
| Cmamp | Mitochondrial adenine monophosphate nucleotide concentration | 0.1 | mM |
| Cmgtp | Mitochondrial guanine triphosphate nucleotide concentration | 0.1 | mM |
| Cmgdp | Mitochondrial guanine diphosphate nucleotide concentration | 0.1 | mM |
| pHm | Mitochondrial pH | 8 |  |
| pHi | Intracellular pH | 7.3 |  |
| Mg | Cytosolic magnesium concentration | 0.7 | mM |
| MgADP | Cytosolic MgADP concentration | 0.46 | mM |
| MgATP | Cytosolic MgATP concentration | 2.69 | mM |
| Ncd | Total cytosolic NAD concentration | 0.32 | mM |
| Ndp | Total cytosolic NADP concentration | 0.065 | mM |
| Ccpi | Cytosolic phosphate concentration | 2.5 | mM |
| Cmpi | Mitochondrial phosphate concentration | 2.5 | mM |
| Cc23p2g | Cytosolic 2,3-bisphosphoglycerate concentration | 3.1 | mM |
| Ccg16p | Cytosolic glucose 1,6-bisphosphate concentration | 0.1 | mM |
| Ccala | Cytosolic alanine concentration | 1 | mM |
| Cmcoq | Mitochondrial oxidized ubiquinol concentration | 1.08 | mM |
| Cmqh2 | Mitochondrial reduced ubiquinol concentration | 0.27 | mM |
| Ccco2 | Cytosolic CO_2_ concentration | 1.2 | mM |
| Cmco2 | Mitochondrial CO_2_ concentration | 21.4 | mM |
| Cccoash | Cytosolic Coenzyme A concentration | 0.02 | mM |
| Ccaccoa | Cytosolic Acetyl-Coenzyme A concentration | 0.001 | mM |
| Cmcoash | Mitochondrial Coenzyme A concentration | 0.04 | mM |
| Cmnad | Mitochondrial NAD concentration | 2.87 | mM |
| Cmnadh | Mitochondrial NADH concentration | 0.1 | mM |
