# Supplementary material for: Multiplicity of Steady States in Glycolysis and Shift of Metabolic State in Cultured Mammalian Cells
Source: PLoS One. 2015 Mar 25;10(3):e0121561. doi: 10.1371/journal.pone.0121561 (PMC4373774; doi:10.1371/journal.pone.0121561)
Supplement: S4 Table — (DOCX) [file pone.0121561.s010.docx]

**S4 Table.** Bounds of enzyme activity within which bistability is observed

| **Enzyme** | **Lower bound** | **Upper bound** |
| --- | --- | --- |
| ***Glycolysis*** |  |  |
| HK | 0.4 | 2 |
| GPI | < 0.1 | > 10 |
| PFK | 0.4 | > 10 |
| PFKFB | < 0.1 | > 10 |
| ALDO | 0.2 | > 10 |
| TPI | < 0.1 | > 10 |
| GAPDH | 0.2 | > 10 |
| PGK | < 0.1 | > 10 |
| PGM | < 0.1 | > 10 |
| ENO | < 0.1 | > 10 |
| PK | < 0.1 | > 10 |
| LDH | < 0.1 | > 10 |
| ***Pentose Phosphate Pathway*** |  |  |
| G6PD | < 0.1 | > 10 |
| 6PGD | < 0.1 | > 10 |
| RPE | < 0.1 | > 10 |
| RPI | < 0.1 | > 10 |
| TK1 | < 0.1 | > 10 |
| TK2 | < 0.1 | > 10 |
| TA | < 0.1 | > 10 |
| ***TCA Cycle*** |  |  |
| PDHC | 0.5 | 2 |
| CS | < 0.1 | > 10 |
| ACON | < 0.1 | > 10 |
| IDH | < 0.1 | > 10 |
| AKGD | < 0.1 | > 10 |
| SCOAS | 0.4 | > 10 |
| SDH | < 0.1 | > 10 |
| FUM | < 0.1 | > 10 |
| MDH2 | < 0.1 | > 10 |
| ***Malate-Aspartate Shuttle Cycle*** |  |  |
| MDH1 | < 0.1 | > 10 |
| GOT1 | < 0.1 | > 10 |
| GOT2 | < 0.1 | > 10 |
| AKGMAL | < 0.1 | > 10 |
| ASPGLU | < 0.1 | > 10 |
| ***Transporters*** |  |  |
| GLUT1 | < 0.1 | > 10 |
| PYRH | < 0.1 | > 10 |
| GLUH | < 0.1 | > 10 |
| CITMAL | < 0.1 | > 10 |
| MALPi | < 0.1 | > 10 |
| ***Other reactions*** |  |  |
| PC | < 0.1 | > 10 |
| CMALIC | < 0.1 | > 10 |
| MMALIC | < 0.1 | > 10 |
| GPT1 | < 0.1 | > 10 |
| GLS | < 0.1 | > 10 |
| GDH | < 0.1 | > 10 |
| CLY | < 0.1 | > 10 |
