# Supplementary material for: Multiplicity of Steady States in Glycolysis and Shift of Metabolic State in Cultured Mammalian Cells
Source: PLoS One. 2015 Mar 25;10(3):e0121561. doi: 10.1371/journal.pone.0121561 (PMC4373774; doi:10.1371/journal.pone.0121561)
Supplement: S1 File — The file contains description of mathematical model rate equations, differential equations and stability analysis. (DOC) [file pone.0121561.s011.doc]

Supplementary Materials

[**Rate Equations 1**](#__RefHeading___Toc401146135)

[Glycolysis 1](#__RefHeading___Toc401146136)

[Pentose Phosphate Pathway 9](#__RefHeading___Toc401146137)

[TCA Cycle 13](#__RefHeading___Toc401146138)

[NAD/NADH Shuttles 18](#__RefHeading___Toc401146139)

[Transporters 21](#__RefHeading___Toc401146140)

[Other Reactions 22](#__RefHeading___Toc401146141)

[Differential Equations 27](#__RefHeading___Toc401146142)

[Glycolysis 27](#__RefHeading___Toc401146143)

[Pentose Phosphate Pathway 28](#__RefHeading___Toc401146144)

[TCA Cycle 29](#__RefHeading___Toc401146145)

[NAD/NADH Shuttles 30](#__RefHeading___Toc401146146)

[Other Equations 30](#__RefHeading___Toc401146147)

[Stability Analysis 31](#__RefHeading___Toc401146148)

[References 31](#__RefHeading___Toc401146149)

# Rate Equations

### Glycolysis

**Hexokinase (HK)**: The rate equation for HK was taken from Mulquiney et al. . The kinetic constants which correspond to those of the isozyme HK1 were adopted from previous literature . The rate equation employs the partial rapid equilibrium random bi bi mechanism with the assumption that all the steps in the mechanism, except for the reactive-ternary complexes, are fast reactions. The inhibitions by *g6p*, glucose-1,6-phosphate (*g16bp*), 2,3-bisphosphoglycerate (*2,3bpg*) and glutathione (*gsh*) were modeled as mixed type of inhibition affecting both the activity (V_max_) as well as the affinity (K_M_) of the enzyme for glucose.

**Eq. S1:**

$$\begin{matrix} r_{HK}=\left(V_{mf}^{HK}\frac{C_{MgATP}^{c}C_{glc}^{c}}{K_{MgATP}^{HK}K_{glc}^{HK}}-V_{mr}^{HK}\frac{C_{MgADP}^{c}C_{g6p}^{c}}{K_{i,MgADP}^{HK}K_{g6p}^{HK}}\right)\frac{1}{N_{HK}} \\ N_{HK}=\left(1+\frac{C_{MgATP}^{c}}{K_{i,MgATP}^{HK}}+\frac{C_{g6p}^{c}}{K_{i,g6p}^{HK}}+\frac{C_{glc}^{c}}{K_{glc}^{HK}}+\frac{C_{MgATP}^{c}C_{glc}^{c}}{K_{MgATP}^{HK}K_{glc}^{HK}}+\frac{C_{MgADP}^{c}}{K_{i,MgADP}^{HK}}+\frac{C_{MgADP}^{c}C_{g6P}^{c}}{K_{i,MgADP}^{HK}K_{g6P}^{HK}}\right.\text{ } \\ \text{ }\left.+\frac{C_{glc}^{c}C_{g6P}^{c}}{K_{glc}^{HK}K_{g6P}^{HK}}+\frac{C_{glc}^{c}C_{g16bp}^{c}}{K_{glc}^{HK}K_{i,g16bp}^{HK}}+\frac{C_{glc}^{c}C_{2,3bpg}^{c}}{K_{glc}^{HK}K_{i,2,3bpg}^{HK}}+\frac{C_{glc}^{c}C_{gsh}^{c}}{K_{glc}^{HK}K_{i,gsh}^{HK}}\right) \end{matrix}$$

$$\begin{matrix} V_{mf}^{HK}=9.59*10^{2}mM\text{ }h^{-1} \\ V_{mr}^{HK}=6.18\text{ }mM\text{ }h^{-1} \\ K_{MgATP}^{HK}=1.0\text{ }mM \\ K_{glc}^{HK}=0.1\text{ }mM \\ K_{i,MgADP}^{HK}=1.0\text{ }mM \\ K_{g6p}^{HK}=0.47mM \\ K_{i,g6p}^{"HK}=2*10^{-2}mM \\ K_{i,g16bp}^{HK}=3*10^{-2}mM \\ K_{i,gsh}^{HK}=3.0\text{ }mM \end{matrix}$$

**Glucose Phosphate Isomerase (GPI)**: The rate equation for GPI was taken from Mulquiney et al. . The kinetic constants were adopted from previous literature . The rate equation employs the steady state uni uni reaction kinetics.

**Eq. S2:**

$$r_{GPI}=\frac{V_{mf}^{GPI}\frac{C_{g6p}^{c}}{K_{f}^{GPI}}-V_{mr}^{GPI}\frac{C_{f6p}^{c}}{K_{r}^{GPI}}}{1+\frac{C_{g6p}^{c}}{K_{f}^{GPI}}+\frac{C_{f6p}^{c}}{K_{r}^{GPI}}}\text{ }$$

$$\begin{matrix} V_{mf}^{GPI}=2.4*10^{3}mM\text{ }h^{-1} \\ V_{mr}^{GPI}=\text{ 2.0}*10^{3}mM\text{ }h^{-1} \\ K_{f}^{GPI}=9.6*10^{-1}mM \\ K_{r}^{GPI}=1.23*10^{-1}mM \end{matrix}$$

**Phosphofructokinase (PFK)**: The rate equation for PFK was taken from Mulquiney et al. . The kinetic constants were adopted from previous literature . The rate kinetics was based on the two state allosteric model using ordered bi bi mechanism. The two state model considers that the enzyme can exist in the active or the non-active state as determined by the levels of the activity modulators. These include activators (*f16bp, f26bp, g16bp,* AMP etc) and inhibitors (ATP, Mg etc). These activity modulators are isozyme specific. For example, *f16bp* only stimulates PFKM and PFKL. The fraction of enzyme in the active state is represented by the nonlinear term *N_PFK_* which is a function of the levels of the activity modulators. *L_PFK_* represents the equilibrium constant between the two states of the enzyme in the absence of any substrates. The initial velocity expression for the enzyme fraction in the active state was modeled as partial rapid equilibrium random bi bi steady state equation similar to the HK kinetics.

**Eq. S3:**

$$\begin{matrix} r_{PFK}=\frac{\frac{V_{f}^{PFK}C_{MgATP}^{c}C_{f6p}^{c}}{K_{f6p}^{PFK}K_{MgATP}^{PFK}}-\frac{V_{r}^{PFK}C_{MgADP}^{c}C_{f16bp}^{c}}{K_{f16bp}^{PFK}K_{MgADP}^{PFK}}}{\left(\left(1+\frac{C_{f6p}^{c}}{K_{f6p}^{PFK}}\right)\left(1+\frac{C_{MgATP}^{c}}{K_{MgATP}^{PFK}}\right)+\left(1+\frac{C_{f16bp}^{c}}{K_{f16bp}^{PFK}}\right)\left(1+\frac{C_{MgADP}^{c}}{K_{MgADP}^{PFK}}\right)-1\right)}\frac{1}{N_{PFK}} \\ N_{PFK}=1+\frac{L_{PFK}\left(1+\frac{C_{ATP}^{c}}{K_{ATP}^{PFK}}\right)^{4}\left(1+\frac{C_{Mg}^{c}}{K_{Mg}^{PFK}}\right)^{4}\left(1+\frac{C_{2,3bpg}^{c}}{K_{2,3bpg}^{PFK}}\right)^{4}\left(1+\frac{C_{Lac}^{c}}{K_{Lac}^{PFK}}\right)^{4}}{\left(1+\frac{C_{f6p}^{c}}{K_{f6p}^{PFK}}+\frac{C_{f16bp}^{c}}{K_{f16bp}^{PFK}}\right)^{4}\left(1+\frac{C_{AMP}^{c}}{K_{AMP}^{PFK}}\right)^{4}\left(1+\frac{C_{g16bp}^{c}}{K_{g16bp}^{PFK}}\right)^{4}\left(1+\frac{C_{Pi}^{c}}{K_{Pi}^{PFK}}\right)^{4}\left(1+\frac{C_{f26bp}^{c}}{K_{f26bp}^{PFK}}\right)^{4}} \\ \end{matrix}$$

$$\begin{matrix} V_{f}^{PFK}=2.63*10^{2}mM^{-1}h^{-1} \\ V_{r}^{PFK}=11.53\text{ }mM^{-1}h^{-1} \\ K_{f6p}^{PFK}=6*10^{-2}\text{ }mM \\ K_{MgATP}^{PFK}=6.8*10^{-2}\text{ }mM \\ K_{MgADP}^{PFK}=0.54\text{ }mM \\ K_{ATP}^{PFK}=0.1\text{ }mM \\ K_{AMP}^{PFK}=0.3\text{ }mM \\ K_{Mg}^{PFK}=0.2\text{ }mM \\ \end{matrix}$$

$$\begin{matrix} K_{Pi}^{PFK}=30\text{ }mM \\ K_{f16bp}^{PFK}=0.3\text{ }mM \\ K_{f26bp}^{PFK}=5.5*10^{-3}mM \\ K_{g16bp}^{PFK}=0.1\text{ }mM \\ K_{23bpg}^{PFK}=0.5\text{ }mM \\ L_{PFK}=2*10^{-3} \\ K_{Lac}^{PFK}=30\text{ }mM \end{matrix}$$

**6-Phosphofructo-2-Kinase/Fructose-2,6-Bisphosphatase (PFKFB)**: The rate equation for PFKFB and the kinetic constants were taken from previously reported studies . PFKFB is a bi-functional enzyme with kinase and bisphosphatase activities, each localized to either terminals of the enzyme and are independent of each other’s activity. The kinase domain catalyzes the synthesis of fructose-2,6-bisphosphate (*f26bp*) from fructose-6-phoshate (*f6p*) and the bisphosphatase domain mediates the hydrolysis of *f26bp* to *f6p*. The reaction kinetics for the kinase domain (r_PFK2_) follows the ordered bi bi steady state kinetics, with phosphoenolpyurvate (*pep*) inhibition of the kinase domain modeled as non-competitive inhibition. The bisphosphatase reaction kinetics (r_F2,6BPase_) was modeled as simple Michaelis-Menten kinetics with non-competitive product inhibition by *f6p*. Isozymes of PFKFB vary in their kinase to bisphosphatase activity (K/P) . The effect of isozyme (or K/P) was modeled by changing the Vmax of r_PFK2_ and holding r_F2,6BPase_ constant.

**Eq. S4:**

$\begin{matrix} r_{PFK2}=\frac{V_{f,PFK2}\left(C_{ATP}^{c}C_{f6p}^{c}-\frac{C_{ADP}^{c}C_{f26bp}^{c}}{K_{eq,PFK2}}\right)\left(0.2+\frac{0.8}{1+\frac{K_{AKT}}{pAKT}}\right)}{\left(K_{i,ATP}^{PFK2}K_{m,f6p}^{PFK2}+K_{m,f6p}^{PFK2}C_{ATP}^{c}+K_{m,ATP}^{PFK2}C_{f6p}^{c}+\frac{K_{m,ADP}^{PFK2}C_{f26bp}^{c}}{K_{eq,PFK2}}+C_{ATP}^{c}C_{f6p}^{c}\right.} \\ \begin{matrix} & & + \end{matrix}\frac{K_{m,f26bp}^{PFK2}C_{ADP}^{c}}{K_{eq,PFK2}}+\frac{K_{m,ADP}^{PFK2}C_{ATP}^{c}C_{f26bp}^{c}}{K_{eq,PFK2}K_{i,ATP}^{PFK2}}+\frac{C_{ADP}^{c}C_{f26bp}^{c}}{K_{eq,PFK2}}+\frac{K_{m,ATP}^{PFK2}C_{ADP}^{c}C_{f6p}^{c}}{K_{i,ADP}^{PFK2}} \\ \begin{matrix} & & + \end{matrix}\left.\frac{C_{ATP}^{c}C_{f6p}^{c}C_{f26bp}^{c}}{K_{i,f26bp}^{PFK2}}+\frac{C_{ADP}^{c}C_{f6p}^{c}C_{f26bp}^{c}}{K_{eq,PFK2}K_{i,f6p}^{PFK2}}\right)\left(1+\frac{C_{pep}^{c}}{K_{i,pep}^{PFK2}}\right) \end{matrix}$

$$\begin{matrix} V_{f,PFK2}=300\text{ }mM\text{ }h^{-1} \\ K_{m,ATP}^{PFK2}=0.15\text{ }mM \\ K_{m,f6p}^{PFK2}=0.032\text{ }mM \\ K_{m,f26bp}^{PFK2}=0.008\text{ }mM \\ K_{m,ADP}^{PFK2}=0.062\text{ }mM \\ K_{i,ATP}^{PFK2}=0.15\text{ }mM \\ K_{i,f6p}^{PFK2}=0.001\text{ }mM \\ K_{i,f26bp}^{PFK2}=0.02\text{ }mM \\ K_{i,ADP}^{PFK2}=0.23\text{ }mM \\ K_{i,pep}^{PFK2}=0.013\text{ }mM \\ K_{eq}^{PFK2}=16\text{ } \\ K_{AKT}=0.5 \\ \end{matrix}$$

$$r_{F2,6BPase}=\frac{V_{F2,6BPase}C_{f26bp}^{c}}{\left(1+\frac{C_{f6p}^{c}}{K_{i,f6p}^{F2,6BPase}}\right)\left(K_{m,f26bp}^{F2,6BPase}+C_{f26bp}^{c}\right)}$$

$$\begin{matrix} V_{F2,6BPase}=13.86\text{ }mM^{-1}h^{-1} \\ K_{m,f26bp}^{F2,6BPase}=10^{-3}\text{ }mM \\ K_{i,f6p}^{F2,6BPase}=25*10^{-3}\text{ }mM \end{matrix}$$

**Aldolase (ALDO):** The rate equation for ALDO was taken from Mulquiney et al. . The kinetic constants were adopted or estimated from previous literature . The reaction kinetics of ALDO follows the ordered uni bi steady state kinetics. Inhibition due to *2,3bpg* as described in the original expression was retained in this study. However, since *2,3bpg* is not a reaction intermediate considered in the model, its concentration was held constant for the purpose of this study.

**Eq. S5:**

$$\begin{matrix} r_{ALD}=\frac{\frac{V_{mf}^{ALD}C_{f16bp}^{c}}{K_{f16bp}^{ALD}}-\frac{V_{mr}^{ALD}C_{gap}^{c}C_{dhap}^{c}}{K_{gap}^{ALD}K_{i,dhap}^{ALD}}}{\left(1+\frac{C_{2,3bpg}^{c}}{K_{i,2,3bpg}^{ALD}}+\frac{C_{f16bp}^{c}}{K_{f16bp}^{ALD}}+\frac{K_{dhap}^{ALD}C_{gap}^{c}}{K_{gap}^{ALD}K_{i,dhap}^{ALD}}\left(1+\frac{C_{2,3bpg}^{c}}{K_{i,2,3bpg}^{ALD}}\right)+\frac{C_{dhap}^{c}}{K_{i,dhap}^{ALD}}\right.} \\ \begin{matrix} \begin{matrix} & \end{matrix} & \end{matrix}\left.+\frac{K_{dhap}^{ALD}C_{F1,6P}^{c}C_{gap}^{c}}{K_{i,f16bp}^{ALD}K_{gap}^{ALD}K_{i,dhap}^{ALD}}+\frac{C_{dhap}^{c}C_{gap}^{c}}{K_{gap}^{ALD}K_{i,dhap}^{ALD}}\right) \end{matrix}$$

$$\begin{matrix} V_{mf}^{ALD}=1.33*10^{2}mM\text{ }h^{-1} \\ V_{mr}^{ALD}=\text{ }4.57*10^{2}mM\text{ }h^{-1} \\ K_{f16bp}^{ALD}=5*10^{-2}mM \\ K_{i,f16bp}^{ALD}=1.98*10^{-2}mM \\ K_{dhap}^{ALD}=3.5*10^{-2}mM \\ K_{i,dhap}^{ALD}=1.1*10^{-2}mM \\ K_{gap}^{ALD}=0.189mM \\ K_{i,2,3bpg}^{ALD}=1.5mM \end{matrix}$$

**Triose Phosphate Isomerase (TPI)**: The rate equation for TPI was taken from Mulquiney et al. . The kinetic constants were adopted from previous literature . The rate kinetics of TPI follows a simple steady state uni uni reaction kinetics.

**Eq. S6:**

$$r_{TPI}=\frac{V_{mf}^{TPI}\frac{C_{dhap}^{c}}{K_{f}^{TPI}}-V_{mr}^{TPI}\frac{C_{gap}^{c}}{K_{r}^{TPI}}}{1+\frac{C_{dhap}^{c}}{K_{f}^{TPI}}+\frac{C_{gap}^{c}}{K_{r}^{TPI}}}$$

$$\begin{matrix} V_{mf}^{TPI}=5.10*10^{2}mM\text{ }h^{-1} \\ V_{mr}^{TPI}=2.76*10^{3}mM\text{ }h^{-1} \\ K_{f}^{TPI}=1.62*10^{-1}mM \\ K_{r}^{TPI}=4.30*10^{-1}mM \end{matrix}$$

**Glyceraldehyde 3-Phosphate Dehydrogenase (GAPDH)**: The rate equation for GAPDH was taken from Mulquiney et al. . The kinetic constants were adopted from previous literature . The rate kinetics of GAPDH follows the ter ter (bi uni uni bi ping pong) steady state kinetics.

**Eq. S7:**

$$\begin{matrix} r_{GAPD}=\frac{V_{mf}^{GAPD}\frac{C_{NAD^{+}}^{c}C_{Pi}^{c}C_{gap}^{c}}{K_{NAD^{+}}^{GAPD}K_{i,Pi}^{GAPD}K_{i,gap}^{GAPD}}-V_{mr}^{GAPD}\frac{C_{1,3bpg}^{c}C_{NADH}^{c}C_{H^{+}}^{c}}{K_{i,1,3bpg}^{GAPD}K_{NADH}^{GAPD}}}{\begin{matrix} \frac{C_{gap}^{c}}{K_{i,gap}^{GAPD}}\left(1+\frac{C_{gap}^{c}}{K_{i,gap}^{'GAPD}}\right)+\frac{C_{1,3bpg}^{c}}{K_{i,1,3bpg}^{GAPD}}\left(1+\frac{C_{gap}^{c}}{K_{i,GAP}^{'GAPD}}\right)+\frac{K_{1,3bpg}^{GAPD}C_{NADH}^{c}C_{H^{+}}^{c}}{K_{i,1,3bpg}^{GAPD}K_{NADH}^{GAPD}}+\frac{K_{gap}^{GAPD}C_{NAD^{+}}^{c}C_{Pi}^{c}}{K_{NAD^{+}}^{GAPD}K_{i,Pi}^{GAPD}K_{i,gap}^{GAPD}} \\ +\frac{C_{NAD^{+}}^{c}C_{gap}^{c}}{K_{i,NAD^{+}}^{GAPD}K_{i,gap}^{GAPD}}+\frac{C_{Pi}^{c}C_{gap}^{c}}{K_{i,Pi}^{GAPD}K_{i,gap}^{GAPD}}\left(1+\frac{C_{gap}^{c}}{K_{i,gap}^{'GAPD}}\right)+\frac{C_{NAD^{+}}^{c}C_{1,3bpg}^{c}}{K_{i,NAD^{+}}^{GAPD}K_{i,1,3bpg}^{GAPD}}+\frac{K_{1,3bpg}^{GAPD}C_{Pi}^{c}C_{NADH}^{c}C_{H^{+}}^{c}}{K_{i,Pi}^{GAPD}K_{i,1,3bpg}^{GAPD}K_{NADH}^{GAPD}} \\ +\frac{C_{gap}^{c}C_{NADH}^{c}C_{H^{+}}^{c}}{K_{i,gap}^{GAPD}K_{i,NADH}^{GAPD}}+\frac{C_{1,3bpg}^{c}C_{NADH}^{c}C_{H^{+}}^{c}}{K_{i,1,3bpg}^{GAPD}K_{NADH}^{GAPD}}+\frac{C_{NAD^{+}}^{c}C_{Pi}^{c}C_{gap}^{c}}{K_{NAD^{+}}^{GAPD}K_{i,Pi}^{GAPD}K_{i,gap}^{GAPD}}+\frac{K_{gap}^{GAPD}C_{NAD^{+}}^{c}C_{Pi}^{c}C_{1,3bpg}^{c}}{K_{i,gap}^{GAPD}K_{NAD^{+}}^{GAPD}K_{i,Pi}^{GAPD}K_{i,1,3bpg}^{'GAPD}} \\ +\frac{C_{Pi}^{c}C_{gap}^{c}C_{NADH}^{c}C_{H^{+}}^{c}}{K_{i,Pi}^{GAPD}K_{i,gap}^{GAPD}K_{i,NADH}^{GAPD}}+\frac{C_{Pi}^{c}C_{1,3bpg}^{c}C_{NADH}^{c}C_{H^{+}}^{c}}{K_{i,1,3bpg}^{GAPD}K_{NADH}^{GAPD}K_{i,Pi}^{GAPD}K_{i,1,3bpg}^{'GAPD}} \end{matrix}} \\ \\ \end{matrix}$$

$$\begin{matrix} V_{mf}^{GAPD}=5.317*10^{3}mMh^{-1} \\ V_{mr}^{GAPD}=3.919*10^{3}mMh^{-1} \\ K_{NAD^{+}}^{GAPD}=0.045mM \\ K_{i,NAD^{+}}^{GAPD}=0.045mM \\ K_{Pi}^{GAPD}=2.5mM \\ K_{i,Pi}^{GAPD}=2.5mM \\ K_{gap}^{GAPD}=0.095mM \\ K_{i,gap}^{GAPD}=1.59*10^{-16}mM \\ K_{i,gap}^{'GAPD}=0.031mM \\ K_{NADH}^{GAPD}=0.0033mM \\ K_{i,NADH}^{GAPD}=0.01mM \\ K_{1,3bpg}^{GAPD}=0.000671mM \\ K_{i,1,3bpg}^{GAPD}=1.52*10^{-18}mM \\ K_{i,1,3bpg}^{'GAPD}=0.001mM \\ K_{eq}^{GAPD}=1.9*10^{-8} \end{matrix}$$

**Phosphoglycerate Kinase (PGK)**: The rate equation for PGK was taken from Mulquiney et al. . The kinetic constants were adopted from previous literature . The rate kinetics of PGK follows the partial rapid equilibrium random bi bi steady state kinetics.

**Eq. S8:**

$$r_{PGK}=\frac{V_{mf}^{PGK}\frac{C_{1,3bpg}^{c}C_{MgADP}^{c}}{K_{i,MgADP}^{PGK}K_{1,3bpg}^{PGK}}-V_{mr}^{PGK}\frac{C_{3pg}^{c}C_{MgATP}^{c}}{K_{i,MgATP}^{PGK}K_{3pg}^{PGK}}}{1+\frac{C_{1,3bpg}^{c}}{K_{i,1,3bpg}^{PGK}}+\frac{C_{MgADP}^{c}}{K_{i,MgADP}^{PGK}}+\frac{C_{1,3bpg}^{c}C_{MgADP}^{c}}{K_{i,MgADP}^{PGK}K_{1,3bpg}^{PGK}}+\frac{C_{3pg}^{c}}{K_{i,3pg}^{PGK}}+\frac{C_{MgATP}^{c}}{K_{i,MgATP}^{PGK}}+\frac{C_{3pg}^{c}C_{MgATP}^{c}}{K_{i,MgATP}^{PGK}K_{3pg}^{PGK}}}$$

$$\begin{matrix} V_{mf}^{PGK}=\text{5.96*10}^{4}mMh^{-1} \\ V_{mr}^{PGK}=2.39\text{*10}^{4}mMh^{-1} \\ K_{MgADP}^{PGK}=0.1mM \\ K_{i,MgADP}^{PGK}=0.08mM \\ K_{1,3bpg}^{PGK}=0.002mM \\ K_{i,1,3bpg}^{PGK}=1.6mM \\ K_{MgATP}^{PGK}=1mM \\ K_{i,MgATP}^{PGK}=0.186mM \\ K_{3pg}^{PGK}=1.1mM \\ K_{i,3pg}^{PGK}=0.205mM \\ K_{eq}^{PGK}=3.2*10^{3} \end{matrix}$$

**Phosphoglycerate Mutase (PGM)**: The rate equation for PGM was taken from Mulquiney et al. . The kinetic constants were adopted from previous literature . The rate kinetics of PGM follows the uni uni steady state kinetics.

**Eq. S9:**

$$r_{PGAM}=\frac{V_{mf}^{PGAM}\frac{C_{3pg}^{c}}{K_{3pg}^{PGAM}}-V_{mr}^{PGAM}\frac{C_{2pg}^{c}}{K_{2pg}^{PGAM}}}{1+\frac{C_{3pg}^{c}}{K_{3pg}^{PGAM}}+\frac{C_{2pg}^{c}}{K_{2pg}^{PGAM}}}$$

$$\begin{matrix} V_{mf}^{PGAM}=\text{4.894*10}^{5}mMh^{-1} \\ V_{mr}^{PGAM}=\text{4.395*10}^{5}mMh^{-1} \\ K_{3pg}^{PGAM}=0.168mM \\ K_{2pg}^{PGAM}=0.0256mM \\ K_{eq}^{PGAM}=0.17 \end{matrix}$$

**Enolase (ENO)**: The rate equation for ENO was taken from Mulquiney et al. . The kinetic constants were adopted from previous literature . The rate kinetics of ENO follows the partial rapid equilibrium random bi bi steady state kinetics.

**Eq. S10:**

$$r_{ENO}=\frac{V_{mf}^{ENO}\frac{C_{2pg}^{c}C_{Mg^{2+}}^{c}}{K_{i,Mg^{2+}}^{ENO}K_{2pg}^{ENO}}-V_{mr}^{ENO}\frac{C_{pep}^{c}C_{Mg^{2+}}^{c}}{K_{i,Mg^{2+}}^{ENO}K_{pep}^{ENO}}}{1+\frac{C_{2pg}^{c}}{K_{i,2pg}^{ENO}}+\frac{C_{Mg^{2+}}^{c}}{K_{i,Mg^{2+}}^{ENO}}+\frac{C_{2pg}^{c}C_{Mg^{2+}}^{c}}{K_{i,Mg^{2+}}^{ENO}K_{2pg}^{ENO}}+\frac{C_{pep}^{c}}{K_{i,pep}^{ENO}}+\frac{C_{Mg^{2+}}^{c}}{K_{i,Mg^{2+}}^{ENO}}+\frac{C_{pep}^{c}C_{Mg^{2+}}^{c}}{K_{i,Mg^{2+}}^{ENO}K_{pep}^{ENO}}}$$

$$\begin{matrix} V_{mf}^{ENO}=\text{ 2.106*10}^{4}mMh^{-1} \\ V_{mr}^{ENO}=\text{5.542*10}^{3}mMh^{-1} \\ K_{i,Mg^{2+}}^{ENO}=K_{Mg^{2+}}^{ENO}=0.14mM \\ K_{pep}^{ENO}=K_{PEP}^{ENO}=0.11mM \\ K_{2pg}^{ENO}=K_{2PG}^{ENO}=.046mM \\ K_{eq}^{ENO}=3.0 \end{matrix}$$

**Pyruvate Kinase (PK)**: The rate equation for PK was taken from Mulquiney et al. . The kinetic constants were adopted from previous literature . Like PFK, the rate kinetics of PK was based on the two state allosteric model using the ordered bi bi mechanism. The two state model considers that the enzyme can exist in active or non-active state determined by the levels of the activity modulators. These include activators (*f16bp, pep, pyr* etc) and inhibitors (ATP*, ala* etc). The fraction of the enzyme in the active state is represented by the nonlinear term *N_PK_* which is a function of levels of activity modulators. *L_PK_* represents the equilibrium constant between enzymes at the two states in the absence of any substrates. The initial velocity expression for the enzyme fraction in the active state is modeled as partial rapid equilibrium random bi bi steady state equation.

**Eq. S11:**

$$\begin{matrix} r_{PK}=\left(\frac{V_{mf}^{PK}\frac{C_{pep}^{c}}{K_{pep}^{PK}}\frac{C_{MgADP}^{c}}{K_{MgADP}^{PK}}-V_{mr}^{PK}\frac{C_{pyr}^{c}}{K_{pyr}^{PK}}\frac{C_{MgATP}^{c}}{K_{MgATP}^{PK}}}{\left(1+\frac{C_{pep}^{c}}{K_{pep}^{PK}}\right)\left(1+\frac{C_{MgADP}^{c}}{K_{MgADP}^{PK}}\right)+\left(1+\frac{C_{pyr}^{c}}{K_{pyr}^{PK}}\right)\left(1+\frac{C_{MgATP}^{c}}{K_{MgATP}^{PK}}\right)-1}\right)\frac{1}{N_{PK}}\text{ } \\ \text{ } \\ N_{PK}=1+L_{PK}\frac{\left(1+\frac{C_{ATP}^{c}}{K_{ATP}^{PK}}\right)^{4}\left(1+\frac{C_{ala}^{c}}{K_{ala}^{PK}}\right)^{4}}{\left(1+\frac{C_{pep}^{c}}{K_{pep}^{PK}}+\frac{C_{pyr}^{c}}{K_{pyr}^{PK}}\right)^{4}\left(1+\frac{C_{f16bp}^{c}}{K_{f16bp}^{PK}}+\frac{C_{g16bp}^{c}}{K_{g16bp}^{PK}}\right)^{4}} \end{matrix}$$

$$\begin{matrix} V_{mf}^{PK}=2.02*10^{3}mM\text{ }h^{-1}\text{ } \\ V_{mf}^{PK}=4.75mM\text{ }h^{-1} \\ K_{pep}^{PK}=2.25*10^{-1}mM \\ K_{MgADP}^{PK}=4.74*10^{-1}mM \\ K_{MgATP}^{PK}=3\text{ }mM \\ K_{ATP}^{PK}=3.39\text{ }mM \\ K_{pyr}^{PK}=4\text{ }mM \\ K_{f16bp}^{PK}=5.0*10^{-3}mM \\ K_{g16bp}^{PK}=0.1\text{ }mM \\ L_{PK}=0.398 \\ K_{ala}^{PK}=0.02\text{ }mM \end{matrix}$$

**Lactate Dehydrogenase (LDH)**: The rate equation for LDH and the kinetic constants were adopted from previous literature . The kinetics of LDH was modeled as ordered bi bi steady state kinetics, with substrate inhibition by pyruvate.

**Eq. S12:**

$$\begin{matrix} r_{LDH}=\frac{V_{mf}^{LDH}\frac{C_{NADH}^{c}C_{pyr}^{c}}{K_{i,NADH}^{LDH}K_{pyr}^{LDH}}-V_{mr}^{LDH}\frac{C_{NAD}^{c}C_{lac}^{c}}{K_{i,NAD}^{LDH}K_{lac}^{LDH}}}{\begin{matrix} \left(1+\frac{K_{NADH}^{LDH}C_{pyr}^{c}}{K_{i,NADH}^{LDH}K_{pyr}^{LDH}}+\frac{K_{NAD}^{LDH}C_{lac}^{c}}{K_{i,NAD}^{LDH}K_{lac}^{LDH}}\right)\left(1+\frac{C_{pyr}^{c}}{K_{i,pyr}^{"LDH}}\right)+\frac{C_{NADH}^{c}}{K_{i,NADH}^{LDH}}+\frac{C_{NAD}^{c}}{K_{i,NAD}^{LDH}} \\ +\frac{C_{NADH}^{c}C_{pyr}^{c}}{K_{i,NADH}^{LDH}K_{pyr}^{LDH}}+\frac{K_{NAD}^{LDH}C_{NADH}^{c}C_{lac}^{c}}{K_{i,NAD}^{LDH}K_{i,NADH}^{LDH}K_{lac}^{LDH}}+\frac{K_{NADH}^{LDH}C_{NAD}^{c}C_{pyr}^{c}}{K_{i,NAD}^{LDH}K_{i,NADH}^{LDH}K_{pyr}^{LDH}}+\frac{C_{NAD}^{c}C_{lac}^{c}}{K_{i,NAD}^{LDH}K_{lac}^{LDH}} \\ +\frac{C_{NADH}^{c}C_{pyr}^{c}C_{lac}^{c}}{K_{i,NADH}^{LDH}K_{pyr}^{LDH}K_{i,lac}^{LDH}}+\frac{C_{NAD}^{c}C_{pyr}^{c}C_{lac}^{c}}{K_{i,NAD}^{LDH}K_{i,pyr}^{LDH}K_{lac}^{LDH}} \end{matrix}} \\ \end{matrix}$$

$$\begin{matrix} V_{mf}^{LDH}=8.66*10^{2}mMh^{-1} \\ V_{mr}^{LDH}=2.17*10^{2}mMh^{-1} \\ K_{pyr}^{LDH}=0.2\text{ }mM \\ K_{i,pyr}^{LDH}=0.228\text{ }mM \\ K_{NAD}^{LDH}=0.107\text{ }mM \\ K_{i,NAD}^{LDH}=0.503\text{ }mM \\ K_{lac}^{LDH}=10.1\text{ }mM \\ K_{i,lac}^{LDH}=30\text{ }mM \\ K_{i,NADH}^{LDH}=2.45*10^{-3}\text{ }mM \\ K_{NADH}^{LDH}=8.44*10^{-3}\text{ }mM \\ K_{i,pyr}^{"LDH}=0.101\text{ }mM \end{matrix}$$

### Pentose Phosphate Pathway

**Glucose 6-phosphate Dehydrogenase (G6PD)**

G6PD is part of oxidative pentose phosphate pathway. Kinetics of G6PD was modeled as ordered bi bi steady state kinetics . The rate equation for G6PD was taken from . The kinetic constants were adopted from previous literature .

**Eq. S13:**

$$\begin{matrix} V_{mf}^{G6PD}=4.39\text{*}10^{20}mM^{-1}h^{-1} \\ V_{mr}^{G6PD}=\text{7.48*10}^{19}mM^{-1}h^{-1} \\ \\ \\ \end{matrix}$$

**6-Phosphogluconate Dehydrogenase (6PGD)**:

6PGD is part of oxidative pentose phosphate pathway. Kinetics of 6PGD was modeled as ordered sequential bi ter steady state kinetics . The rate equation for 6PGD was taken from . The kinetic constants were adopted from previous literature .

**Eq. S14:**

$$\begin{matrix} r_{6PGD}=\frac{V_{mf}^{6PGD}C_{NADP}^{c}C_{6pg}^{c}-V_{mr}^{6PGD}C_{ru5p}^{c}C_{NADPH}^{c}CO_{2}}{D_{6PGD}} \\ D_{6PGD}=5.38*10^{15}+7.86*10^{19}C_{NADPH}^{c}+6.97*10^{18}C_{NADP}^{c}+ \\ C_{ru5p}^{c}\left(1.04*10^{18}+3.99*10^{23}C_{NADPH}^{c}+3.03*10^{21}C_{NADP}^{c}\right)+ \\ C_{6pg}^{c}\left(7.26*10^{18}+2.40*10^{23}C_{NADPH}^{c}+4.46*10^{23}C_{NADP}^{c}+C_{ru5p}^{c}\left(2.63*10^{27}C_{NADPH}^{c}+3.19*10^{27}C_{NADP}^{c}\right)\right) \\ \text{ } \end{matrix}$$

$$\begin{matrix} V_{mf}^{6PGD}=1.83*10^{19}mM^{-1}h^{-1} \\ V_{mr}^{6PGD}=9.0*10^{19}mM^{-2}h^{-1} \\ \\ \\ \end{matrix}$$

**Ribulose Phosphate Epimerase (RPE)**

RPE is part of non-oxidative pentose phosphate pathway. Kinetics of RPE was modeled as steady state uni uni mechanism. The kinetic constants were adopted from previous literature .

**Eq. S15:**

$$r_{RPE}=\frac{V_{mf}^{RPE}\frac{C_{ru5p}^{c}}{K_{f}^{RPE}}-V_{mr}^{RPE}\frac{C_{xyl5p}^{c}}{K_{r}^{RPE}}}{1+\frac{C_{ru5p}^{c}}{K_{f}^{RPE}}+\frac{C_{xyl5p}^{c}}{K_{r}^{RPE}}}$$

$$\begin{matrix} V_{mf}^{RPE}=4.642*10^{4}mM\text{ }h^{-1} \\ V_{mr}^{RPE}=6.667*10^{4}mM\text{ }h^{-1} \\ K_{f}^{RPE}=1.90*10^{-1}mM \\ K_{r}^{RPE}=5.00*10^{-1}mM \end{matrix}$$

**Ribose Phosphate Isomerase (RPI)**

RPI is part of non-oxidative pentose phosphate pathway. Kinetics of RPI was modeled as steady state uni uni mechanism. The kinetic constants were adopted from previous literature .

**Eq. S16:**

$$r_{RPI}=\frac{V_{mf}^{RPI}\frac{C_{ru5p}^{c}}{K_{f}^{RPI}}-V_{mr}^{RPI}\frac{C_{r5p}^{c}}{K_{r}^{RPI}}}{1+\frac{C_{ru5p}^{c}}{K_{f}^{RPI}}+\frac{C_{r5p}^{c}}{K_{r}^{RPI}}}$$

$$\begin{matrix} V_{mf}^{RPI}=2.56*10^{4}mM\text{ }h^{-1} \\ V_{mr}^{RPI}=1.09*10^{4}mM\text{ }h^{-1} \\ K_{f}^{RPI}=7.80*10^{-1}mM \\ K_{r}^{RPI}=2.20\text{ }mM \end{matrix}$$

**Phosphoribosylpyrophosphate Synthetase (PRPPS)**

PRPPS channels ribose-5-phosphate (*r5p*) towards nucleotide synthesis. The rate is relatively small portion of total PPP flux. Kinetics of PRPPS was modeled as rapid equilibrium random bi reactant system. The kinetic constants were adopted from previous literature .

**Eq. S17:**

$$r_{PRPPS}=V_{m}^{PRPPS}\frac{C_{MgATP}^{c}C_{r5p}^{c}}{\left(K_{MgATP}^{PRPPS}+C_{MgATP}^{c}\right)\left(K_{r5p}^{PRPPS}+C_{r5p}^{c}\right)}$$

$$\begin{matrix} V_{m}^{PRPPS}=25.3\text{ }mM\text{ }h^{-1} \\ K_{MgATP}^{PRPPS}=0.01\text{ }mM \\ K_{r5p}^{PRPPS}=0.57\text{ }mM \end{matrix}$$

**Transketolase1 (TK1)**

TK1 is part of non-oxidative pentose phosphate pathway. Kinetics of TK1 was modeled as steady state bi bi ping pong mechanism. The kinetic constants were adopted from previous literature .

**Eq. S18:**

$$\begin{matrix} r_{TK1}=\frac{V_{mf}^{TK1}C_{xyl5p}^{c}C_{r5p}^{c}-V_{mr}^{TK1}C_{s7p}^{c}C_{gap}^{c}}{D_{TK1}} \\ D_{TK1}=2.63*10^{16}C_{s7p}^{c}+C_{r5p}^{c}\left(4.40*10^{16}+4.92*10^{16}C_{s7p}^{c}\right)+\text{ }5.96*10^{16}C_{gap}^{c}+\text{ } \\ 6.94*10^{16}C_{s7p}^{c}C_{gap}^{c}+C_{xyl5P}^{c}\left(\text{ }7.35*10^{16}+2.44*10^{17}C_{r5p}^{c}+3.38*10^{17}C_{gap}^{c}\right)\text{ } \end{matrix}$$

$$\begin{matrix} V_{mf}^{TK1}=5.3210^{18}mM^{-1}h^{-1} \\ V_{mr}^{TK1}=2.58*10^{18}mM^{-1}h^{-1} \\ \\ \end{matrix}$$

**Transaldolase (TA)**

TA is part of non-oxidative pentose phosphate pathway. Kinetics of TA was modeled as steady state bi bi ping pong mechanism. The rate equation for TA was taken from . The kinetic constants were adopted from previous literature .

**Eq. S19:**

$$\begin{matrix} r_{TA}=\frac{V_{mf}^{TA}C_{s7p}^{c}C_{gap}^{c}-V_{mr}^{TA}C_{f6p}^{c}C_{e4p}^{c}}{D_{TA}} \\ D_{TA}=3.4*10^{16}C_{s7p}^{c}+2.38*10^{16}C_{gap}^{c}+1.64*10^{17}C_{e4p}^{c}+2.72*10^{15}C_{f6p}^{c}+4.41*10^{17}C_{s7p}^{c}C_{gap}^{c} \\ \begin{matrix} & & \end{matrix}+7.83*10^{16}C_{e4p}^{c}C_{F6P}^{c}+2.21*10^{16}C_{gap}^{c}C_{f6p}^{c}+2.1*10^{18}C_{s7p}^{c}C_{e4p}^{c} \end{matrix}$$

$$\begin{matrix} V_{mf}^{TA}=1.36*10^{20}mM^{-1}h^{-1} \\ V_{mr}^{TA}=7.53*10^{19}mM^{-1}h^{-1} \\ \\ \end{matrix}$$

**Transketolase2 (TK2)**

TK2 is part of non-oxidative pentose phosphate pathway. Kinetics of TK2 was modeled as steady state bi bi ping pong mechanism. The kinetic constants were adopted from previous literature .

**Eq. S20:**

$$\begin{matrix} r_{TK2}=\frac{V_{mf}^{TK2}C_{xyl5p}^{c}C_{e4p}^{c}-V_{mr}^{TK2}C_{f6p}^{c}C_{gap}^{c}}{D_{TK2}} \\ D_{TK2}=\left(3.01*10^{17}C_{e4p}^{c}+5.96*10^{16}C_{gap}^{c}+C_{f6p}^{c}\left(1.25*10^{16}+1.60*10^{17}C_{e4p}^{c}+3.31*10^{16}C_{gap}^{c}\right)\right)+ \\ C_{xyl5p}^{c}\left(7.35*10^{16}+1.67*10^{18}C_{e4p}^{c}+3.38*10^{17}C_{gap}^{c}\right)\text{ } \end{matrix}$$

$$\begin{matrix} V_{mf}^{TK2}=3.64*10^{19}mM^{-1}h^{-1} \\ V_{mr}^{TK2}=1.23*10^{18}mM^{-1}h^{-1} \\ \\ \end{matrix}$$

**Glutathione Peroxidase (GPX)**

GPX oxidizes the reduced form of glutathione (*gsh*) into glutathione disulfide (*gssg*) while hydrogen peroxide (H_2_O_2_) is reduced into water. Thus this reaction plays an important role in preventing cells from oxidative stress. The kinetics of GPX was modeled as first order reaction kinetics.

**Eq. S21:**

$$r_{GPX}=V_{f}^{GPX}C_{gsh}^{c}$$

$$V_{f}^{GPX}=1.56*10^{4}mM^{-1}h^{-1}$$

**Glutathione Reductase (GSSGR)**

The enzyme GSSGR converts *gssg* back into *gsh* while NADPH produced in the pentose phosphate pathway is oxidized into NADP. The kinetics of GSSGR was modeled as ordered sequential steady state kinetics . The rate equation for GSSGR was taken from . The kinetic constants were adopted from previous literature .

**Eq. S22:**

$$\begin{matrix} r_{GSSGR}=\frac{V_{mf}^{GSSGR}C_{NADPH}^{c}C_{gssg}^{c}-V_{mr}^{GSSGR}C_{gsh}^{c}C_{NADP}^{c}}{D_{GSSGR}} \\ D_{GSSGR}=1.73*10^{40}+2.88*10^{42}C_{NADPH}^{c}+3.43*10^{41}C_{gssg}^{c}+7.77*10^{35}C_{gsh}^{c}+2.47*10^{41}C_{NADP}^{c} \\ +4.02*10^{43}C_{NADPH}^{c}C_{gssg}^{c}+1.3*10^{38}C_{NADPH}^{c}C_{gsh}^{c}+4.90*10^{42}C_{gssg}^{c}C_{NADP}^{c}+5.55*10^{36}C_{gsh}^{c}C_{gsh}^{c} \\ +1.11*10^{37}C_{gsh}^{c}C_{NADP}^{c}+1.24*10^{40}C_{gsh}^{c}C_{NADP}^{c}+3.26*10^{39}C_{NADPH}^{c}C_{gssg}^{c}C_{gsh}^{c} \\ +2.08*10^{41}C_{NADPH}^{c}C_{gssg}^{c}C_{gsh}^{c}+9.25*10^{38}C_{NADPH}^{c}C_{gsh}^{c}C_{gsh}^{c}+2.45*10^{41}C_{gssg}^{c}C_{gsh}^{c}C_{NADP}^{c} \\ +1.78*10^{39}C_{gsh}^{c}C_{gsh}^{c}C_{NADP}^{c}+2.32*10^{40}C_{NADPH}^{c}C_{gssg}^{c}C_{gsh}^{c}C_{gsh}^{c}+2.74*10^{40}C_{gssg}^{c}C_{gsh}^{c}C_{NADP}^{c}C_{gsh}^{c} \end{matrix}$$

$$\begin{matrix} V_{mf}^{GSSGR}=5.5*10^{44}mM^{-1}h^{-1} \\ V_{mr}^{GSSGR}=1.05*10^{40}mM^{-1}h^{-1} \\ \\ \end{matrix}$$

### TCA Cycle

**Pyruvate Dehydrogenase (PDH)**

The rate equation for PDH was taken from . The kinetics of PDH was modeled as hexa uni ping pong mechanism.The kinetic constants were adopted from previous literature .

**Eq. S23:**

$\begin{matrix} V_{f,PDH}=189.7mMh^{-1} \\ K_{eq,PDH}=1.2*10^{4} \\ K_{pyr}^{PDH}=38.8*10^{-3}mM \\ K_{CoASH}^{PDH}=9.9*10^{-3}mM \\ K_{NAD^{+}}^{PDH}=60.7*10^{-3}mM \\ K_{i,AcCoA}^{PDH}=40.0*10^{-3}mM \\ K_{i,NADH}^{PDH}=40.2*10^{-3}mM \\ \\ \end{matrix}$

$$\begin{matrix} r_{PDH}=\frac{V_{f,PDH}\left(C_{pyr}^{m}C_{CoASH}^{m}C_{NAD^{+}}^{m}-\frac{C_{AcCoA}^{m}C_{NADH}^{m}C_{CO_{2}}^{m}}{K_{eq,PDH}}\right)}{K_{NAD^{+}}^{PDH}\alpha_{PDH,i2}C_{pyr}^{m}C_{CoASH}^{m}+K_{CoASH}^{PHD}\alpha_{PDH,i1}C_{pyr}^{m}C_{NAD^{+}}^{m}+K_{pyr}^{PDH}C_{CoASH}^{m}C_{NAD^{+}}^{m}+C_{pyr}^{m}C_{CoASH}^{m}C_{NAD^{+}}^{m}} \\ \\ \alpha_{PDH,i1}=\left(1+\frac{C_{AcCoA}^{m}}{K_{i,AcCoA}^{c}}\right) \\ \alpha_{PDH,i2}=\left(1+\frac{C_{NADH}^{m}}{K_{i,NADH}^{PDH}}\right) \\ \end{matrix}$$

**Citrate Synthase (CS)**

The rate equation for CS was taken from . The kinetic constants were adopted from previous literature . The kinetics of CS was modeled as ordered bi bi mechanism.

**Eq. S24:**

$\begin{matrix} V_{f,CS}=1804\text{ }mMh^{-1} \\ K_{eq,CS}^{0}=6.22*10^{12} \\ K_{OAA}^{CS}=4*10^{-3}mM \\ K_{AcCoA}^{CS}=1.4*10^{-3}mM \\ K_{i,OAA}^{CS}=3.33*10^{-3}mM \\ K_{i,cit}^{CS}=1600*10^{-3}mM \\ K_{i,ATP}^{CS}=900*10^{-3}mM \\ K_{i,ADP}^{CS}=1800*10^{-3}mM \\ K_{i,AMP}^{CS}=6000*10^{-3}mM \\ K_{i,CoASH}^{CS}=67*10^{-3}mM \\ K_{i,scoa}^{CS}=140*10^{-3}mM \end{matrix}$

$$\begin{matrix} r_{CS}=\frac{V_{f,CS}\left(C_{OAA}^{m}C_{AcCoA}^{m}-\frac{C_{CoASH}^{m}C_{cit}^{m}}{K_{eq,CS}}\right)}{K_{i,OAA}^{CS}K_{i,AcCoA}^{CS}\alpha_{CS,i1}+K_{OAA}^{CS}\alpha_{CS,i1}C_{AcCoA}^{m}+K_{AcCoA}^{CS}\alpha_{CS,i2}C_{OAA}^{m}+C_{OAA}^{m}C_{AcCoA}^{m}} \\ \\ \alpha_{CS,i1}=\left(1+\frac{\frac{C_{cit}^{m}}{P_{cit}}}{K_{i,cit}^{CS}}\right) \\ \alpha_{CS,i2}=\left(1+\frac{\frac{C_{ATP}^{m}}{P_{ATP}}}{K_{i,ATP}^{CS}}+\frac{\frac{C_{ADP}^{m}}{P_{ADP}}}{K_{i,ADP}^{CS}}+\frac{\frac{C_{AMP}^{m}}{P_{AMP}}}{K_{i,AMP}^{CS}}+\frac{C_{CoASH}^{m}}{K_{i,CoASH}^{CS}}+\frac{C_{scoa}^{m}}{K_{i,scoA}^{CS}}\right) \\ \end{matrix}$$

**Aconitase (ACON)**

The rate equation for ACON was taken from . The kinetics of ACON employs the steady state uni uni reaction kinetics. The kinetic constants were adopted from previous literature .

**Eq. S25:**

$\begin{matrix} V_{f,ACON}=1*10^{5}mMh^{-1} \\ K_{eq,ACON}=2.7*10^{-2} \\ K_{cit}^{ACON}=1161*10^{-3}mM \\ K_{icit}^{ACON}=434*10^{-3}mM \end{matrix}$

$$r_{ACON}=\frac{V_{f,ACON}\left(C_{cit}^{m}-\frac{C_{icit}^{m}}{K_{eq,ACON}}\right)}{K_{cit}^{ACON}+C_{cit}^{m}+\frac{K_{cit}^{ACON}C_{icit}^{m}}{K_{icit}^{ACON}}}$$

**Isocitrate Dehydrogenase (IDH)**

The rate equation for IDH was taken from . The kinetics of IDH was modeled as ordered bi ter mechanism. The kinetic constants were adopted from previous literature .

**Eq. S26:**

$\begin{matrix} V_{f,AKG}=2.75*10^{4}mMh^{-1} \\ K_{eq,IDH}=30.5 \\ K_{m,NAD^{+}}^{IDH}=74*10^{-3}mM \\ K_{m,icit}^{IDH}=183*10^{-3}mM \\ K_{a,ADP}^{IDH}=50*10^{-3}mM \\ K_{i,ATP}^{IDH}=91*10^{-3}mM \\ K_{i,icit}^{IDH}=23.8*10^{-3}mM \\ K_{i,NADH}^{IDH}=29*10^{-3}mM \\ n_{H}=3 \\ \end{matrix}$

$$\begin{matrix} r_{IDH}=\frac{V_{f,IDH}\left(1-\frac{1}{K_{eq,IDH}}\frac{C_{akg}^{m}C_{NADH}^{m}C_{CO_{2}}^{m}}{C_{NAD^{+}}^{m}C_{icit}^{m}}\right)}{1+\left(\frac{K_{m,icit}^{IDH}}{C_{icit}^{m}}\right)^{n_{H}}\alpha_{IDH,i1}+\frac{K_{m,NAD^{+}}^{IDH}}{C_{NAD^{+}}^{m}}\left(1+\left(\frac{K_{i,icit}^{IDH}}{C_{icit}^{m}}\right)^{n_{H}}\alpha_{IDH,i1}+\frac{C_{NADH}^{m}}{K_{i,NADH}^{IDH}}\alpha_{IDH,i1}\right)} \\ \\ \alpha_{IDH,i1}=\left(1+\frac{K_{a,ADP}^{IDH}P_{ADP}}{C_{ADP}^{m}P_{fADP}}\left(1+\frac{C_{ATP}^{m}P_{fATP}}{K_{i,ATP}^{IDH}P_{ATP}}\right)\right) \end{matrix}$$

**α-Ketoglutarate Dehydrogenase (AKGD)**

The rate equation for AKGD was taken from . The kinetics of AKGD was modeled as hexa uni ping pong ter ter mechanism. The kinetic constants were adopted from previous literature .

**Eq. S27:**

$\begin{matrix} V_{f,AKGD}=2495\text{ }mMh^{-1} \\ K_{eq,AKGD}=1.66*10^{8} \\ K_{m,akg}^{AKGD}=120*10^{-3}mM \\ K_{m,CoaSH}^{AKGD}=55*10^{-3}mM \\ K_{m,NAD^{+}}^{AKGD}=21*10^{-3}mM \\ K_{a,ADP}^{AKGD}=100*10^{-3}mM \\ K_{i,ATP}^{AKGD}=50*10^{-3}mM \\ K_{i,SCoA}^{AKGD}=6.9*10^{-3}mM \\ K_{i,NADH}^{AKGD}=0.60*10^{-3}mM \end{matrix}$

$$\begin{matrix} r_{AKGD}=\frac{V_{f,AKGD}\left(1-\frac{1}{K_{eq,AKGD}}\frac{C_{SCoA}^{m}C_{NADH}^{m}C_{CO_{2}}^{m}}{C_{akg}^{m}C_{CoASH}^{m}C_{NAD^{+}}^{m}}\right)}{1+\left(\frac{K_{m,akg}^{AKGD}}{C_{akg}^{m}}\right)\alpha_{AKGD,i1}+\frac{K_{m,CoASH}^{AKGD}}{C_{CoASH}^{m}}\left(1+\frac{C_{SCoA}^{m}}{K_{i,SCoA}^{AKGD}}\right)+\frac{K_{m,NAD^{+}}^{AKGD}}{C_{NAD^{+}}^{m}}\left(1+\frac{C_{NADH}^{m}}{K_{i,NADH}^{AKGD}}\right)} \\ \\ \alpha_{AKGD,i1}=\left(1+\frac{K_{a,ADP}^{AKGD}P_{ADP}}{C_{ADP}^{m}P_{fADP}}\left(1+\frac{C_{ATP}^{m}P_{fATP}}{K_{i,ATP}^{AKGD}P_{ATP}}\right)\right) \end{matrix}$$

**Succinyl-CoA Synthetase (SCOAS)**

The rate equation for SCOAS was taken from . The kinetics of SCOAS follows the ordered ter ter mechanism. The kinetic constants were adopted from previous literature .

**Eq. S28:**

$\begin{matrix} V_{f,SCoAS}=362 mMh^{-1} \\ K_{eq,SCOAS}=7.43 \\ K_{m,GDP}^{SCoAS}=16*10^{-3}mM \\ K_{m,SCoA}^{SCoAS}=55*10^{-3}mM \\ K_{m,Pi}^{SCoAS}=660.0*10^{-3}mM \\ K_{m,CoaSH}^{SCoAS}=20*10^{-3}mM \\ K_{m,suc}^{SCoAS}=880*10^{-3}mM \\ K_{m,GTP}^{SCoAS}=11.1*10^{-3}mM \\ K_{i,GDP}^{SCoAS}=5.5*10^{-3}mM \\ K_{i,SCoA}^{SCoAS}=100*10^{-3}mM \\ K_{i,Pi}^{SCoAS}=2000*10^{-3}mM \\ K_{i,CoaSH}^{SCoAS}=20*10^{-3}mM \\ K_{i,suc}^{SCoAS}=3000*10^{-3}mM \\ K_{i,GTP}^{SCoAS}=11.1*10^{-3}mM \\ \\ \end{matrix}$

$$\begin{matrix} r_{SCoAS}=\frac{V_{f,SCoAS}\left(C_{GDP}^{m}C_{SCoA}^{m}C_{Pi}^{m}-\frac{C_{CoASH}^{m}C_{suc}^{m}C_{GTP}^{m}}{K_{eq,SCoAS}}\right)}{K_{i,GDP}^{SCoAS}K_{i,SCoA}^{SCoAS}K_{m,Pi}^{SCoAS}+K_{i,SCoA}^{SCoAS}K_{m,Pi}^{SCoAS}C_{GDP}^{m}+K_{i,GDP}^{SCoAS}K_{m,SCoA}^{SCoAS}C_{Pi}^{m}+K_{m,Pi}^{SCoAS}C_{GDP}^{m}C_{SCoA}^{m}+K_{m,SCoA}^{SCoAS}C_{GDP}^{m}C_{Pi}^{m}} \\ K_{m,GDP}^{SCoAS}C_{SCoA}^{m}C_{Pi}^{m}+C_{GDP}^{m}C_{SCoA}^{m}C_{Pi}^{m}+\left(\frac{K_{i,GDP}^{SCoAS}K_{i,SCoA}^{SCoAS}K_{m,Pi}^{SCoAS}}{K_{i,suc}^{SCoAS}K_{i,GTP}^{SCoAS}K_{m,CoASH}^{SCoAS}}\right)\left(K_{i,GTP}^{SCoAS}K_{m,suc}^{SCoAS}C_{CoASH}^{m}+K_{i,suc}^{SCoAS}K_{m,CoASH}^{SCoAS}C_{GTP}^{m}+\right. \\ K_{m,GTP}^{SCoAS}C_{CoASH}^{m}C_{suc}^{m}+K_{m,suc}^{SCoAS}C_{CoASH}^{m}C_{GTP}^{m}+K_{m,CoASH}^{SCoAS}C_{suc}^{m}C_{GTP}^{m}+C_{CoASH}^{m}C_{suc}^{m}C_{GTP}^{m}+\frac{K_{m,suc}^{SCoAS}K_{i,GTP}^{SCoAS}C_{GDP}^{m}C_{SCoA}^{m}C_{suc}^{m}}{K_{i,GDP}^{SCoAS}K_{i,SCoA}^{SCoAS}} \\ +\frac{K_{m,GTP}^{SCoAS}C_{GDP}^{m}C_{CoASH}^{m}C_{suc}^{m}}{K_{i,GDP}^{SCoAS}}+\frac{K_{i,GTP}^{SCoAS}K_{m,suc}^{SCoAS}C_{GDP}^{m}C_{SCoA}^{m}C_{Pi}^{m}C_{CoASH}^{m}}{K_{i,GDP}^{SCoAS}K_{i,SCoA}^{SCoAS}K_{i,Pi}^{SCoAS}}+\frac{K_{m,suc}^{SCoAS}K_{i,GTP}^{SCoAS}C_{GDP}^{m}C_{CoASH}^{m}}{K_{i,GDP}^{SCoAS}} \\ \left.+\frac{K_{i,CoASH}^{SCoAS}K_{m,GTP}^{SCoAS}C_{GDP}^{m}C_{SCoA}^{m}C_{Pi}^{m}C_{suc}^{m}}{K_{i,GDP}^{SCoAS}K_{i,SCoA}^{SCoAS}K_{i,Pi}^{SCoAS}}+\frac{K_{m,GTP}^{SCoAS}C_{GDP}^{m}C_{SCoA}^{m}C_{CoASH}^{m}C_{suc}^{m}}{K_{i,GDP}^{SCoAS}K_{i,SCoA}^{SCoAS}}+\frac{K_{m,GTP}^{SCoAS}C_{GDP}^{m}C_{SCoA}^{m}C_{Pi}^{m}C_{CoASH}^{m}C_{suc}^{m}}{K_{i,GDP}^{SCoAS}K_{i,SCoA}^{SCoAS}K_{i,Pi}^{SCoAS}}\right) \\ +\frac{K_{i,GDP}^{SCoAS}K_{m,SCoA}^{SCoAS}C_{Pi}^{m}C_{suc}^{m}C_{GTP}^{m}}{K_{i,suc}^{SCoAS}K_{i,GTP}^{SCoAS}}+\frac{K_{m,GDP}^{SCoAS}K_{i,Pi}^{SCoAS}C_{SCoA}^{m}C_{CoASH}^{m}C_{suc}^{m}C_{GTP}^{m}}{K_{i,CoASH}^{SCoAS}K_{i,suc}^{SCoAS}K_{i,GTP}^{SCoAS}}+\frac{K_{i,GDP}^{SCoAS}K_{m,SCoA}^{SCoAS}C_{Pi}^{m}C_{CoASH}^{m}C_{suc}^{m}C_{GTP}^{m}}{K_{i,CoASH}^{SCoAS}K_{i,suc}^{SCoAS}K_{i,GTP}^{SCoAS}}+ \\ +\frac{K_{m,GDP}^{SCoAS}C_{SCoA}^{m}C_{Pi}^{m}C_{GTP}^{m}}{K_{i,GTP}^{SCoAS}}+\frac{K_{i,GDP}^{SCoAS}K_{m,SCoA}^{SCoAS}C_{Pi}^{m}C_{GTP}^{m}}{K_{i,GTP}^{SCoAS}}+\frac{K_{m,GDP}^{SCoAS}C_{SCoA}^{m}C_{Pi}^{m}C_{suc}^{m}C_{GTP}^{m}}{K_{i,suc}^{SCoAS}K_{i,GTP}^{SCoAS}}+\frac{K_{m,GDP}^{SCoAS}C_{SCoA}^{m}C_{Pi}^{m}C_{CoASH}^{m}C_{suc}^{m}C_{GTP}^{m}}{K_{i,GDP}^{SCoAS}K_{i,SCoA}^{SCoAS}K_{i,Pi}^{SCoAS}} \end{matrix}$$

**Succinate Dehydrogenase (SDH)**

The rate equation for SDH was taken from . The kinetics of SDH was modeled as Theorell-Chance bi bi mechanism. The kinetic constants were adopted from previous literature .

**Eq. S29:**

$\begin{matrix} V_{f,SDH}=5.81*10^{4} mMh^{-1} \\ K_{eq,SDH}=1.21 \\ K_{m,suc}^{SDH}=467*10^{-3}mM \\ K_{m,COQ}^{SDH}=480*10^{-3}mM \\ K_{m,QH_{2}}^{SDH}=2.45*10^{-3}mM \\ K_{m,fum}^{SDH}=1200*10^{-3}mM \\ K_{i,suc}^{SDH}=120*10^{-3}mM \\ K_{i,fum}^{SDH}=1275*10^{-3}mM \\ K_{i,OAA}^{SDH}=1.5*10^{-3}mM \\ K_{a,suc}^{SCoAS}=450*10^{-3}mM \\ K_{a,fum}^{SCoAS}=375*10^{-3}mM \end{matrix}$

$$\begin{matrix} r_{SDH}=\frac{V_{f,SDH}\left(C_{suc}^{m}C_{COQ}^{m}-\frac{C_{QH_{2}}^{m}C_{fum}^{m}}{K_{eq,SDH}}\right)}{K_{i,suc}^{SDH}K_{m,COQ}^{SDH}\alpha_{SDH,i1}+K_{m,COQ}^{SDH}C_{suc}^{m}+K_{m,suc}^{SDH}\alpha_{SDH,i1}C_{COQ}^{m}+C_{suc}^{m}C_{COQ}^{m}+\frac{K_{m,suc}^{SDH}}{K_{i,fum}^{SDH}}C_{COQ}^{m}C_{fum}^{m}} \\ +\left(\frac{K_{i,suc}^{SDH}K_{m,COQ}^{SDH}}{K_{i,fum}^{SDH}K_{m,QH_{2}}^{SDH}}\right)\left(K_{m,fum}^{SDH}\alpha_{SDH,i1}C_{QH_{2}}^{m}+K_{m,QH_{2}}^{SDH}C_{fum}^{m}+\frac{K_{m,fum}^{SDH}}{K_{i,suc}^{SDH}}C_{suc}^{m}C_{QH_{2}}^{m}+C_{QH_{2}}^{m}C_{fum}^{m}\right) \\ \\ \\ \alpha_{SDH,i1}=\frac{\left(1+\frac{C_{OAA}^{m}}{K_{i,OAA}^{SDH}}+\frac{C_{suc}^{m}}{K_{a,suc}^{SDH}}+\frac{C_{fum}^{m}}{K_{a,fum}^{SDH}}\right)}{\left(1+\frac{C_{suc}^{m}}{K_{a,suc}^{SDH}}+\frac{C_{fum}^{m}}{K_{a,fum}^{SDH}}\right)} \end{matrix}$$

**Fumarase (FUM)**

The rate equation for FUM was taken from . The kinetics of FUM was modeled as ordered uni uni mechanism. The kinetic constants were adopted from previous literature .

**Eq. S30:**

$\begin{matrix} V_{f,FUM}=2.21*10^{4} mMh^{-1} \\ K_{eq,FUM}=4.56 \\ K_{m,fum}^{FUM}=44.7*10^{-3}mM \\ K_{m,mal}^{FUM}=197.7*10^{-3}mM \\ K_{i,cit}^{FUM}=3500*10^{-3}mM \\ K_{i,ATP}^{FUM}=40*10^{-3}mM \\ K_{i,ADP}^{SDH}=400*10^{-3}mM \\ K_{i,GTP}^{SDH}=80*10^{-3}mM \\ K_{i,GDP}^{SDH}=330*10^{-3}mM \end{matrix}$

$$\begin{matrix} r_{FUM}=\frac{V_{f,FUM}\left(C_{fum}^{m}-\frac{C_{mal}^{m}}{K_{eq,FUM}}\right)}{K_{m,fum}^{FUM}\alpha_{FUM,i1}+C_{fum}^{m}+\frac{K_{m,fum}^{FUM}C_{mal}^{m}}{K_{m,mal}^{FUM}}} \\ \alpha_{FUM,i1}=1+\frac{C_{cit}^{m}}{K_{i,cit}^{FUM}}+\frac{C_{ATP}^{m}P_{fATP}}{K_{i,ATP}^{FUM}P_{ATP}}+\frac{C_{ADP}^{m}P_{fADP}}{K_{i,ADP}^{FUM}P_{ADP}}+\frac{C_{GTP}^{m}P_{fGTP}}{K_{i,GTP}^{FUM}P_{GTP}}+\frac{C_{GDP}^{m}P_{fGDP}}{K_{i,GDP}^{FUM}P_{GDP}} \end{matrix}$$

**Malate Dehydrogenase 2 (MDH2)**

MDH2 is the isoform that is present in the mitochondria. The rate equation for MDH2 was taken from . The kinetics of MDH2 employs the ordered bi bi mechanism. The kinetic constants were adopted from previous literature .

**Eq. S31:**

$\begin{matrix} V_{f,MDH2}=3.53*10^{8} mMh^{-1} \\ K_{eq,MDH2}=4.02*10^{-4} \\ K_{m,NAD^{+}}^{MDH2}=90.55*10^{-3}mM \\ K_{m,mal}^{MDH2}=250*10^{-3}mM \\ K_{m,OAA}^{MDH2}=6.128*10^{-3}mM \\ K_{m,NADH}^{MDH2}=2.58*10^{-3}mM \\ K_{i,NAD^{+}}^{MDH2}=279*10^{-3}mM \\ K_{i,mal}^{MDH2}=360*10^{-3}mM \\ K_{i,OAA}^{MDH2}=5.5*10^{-3}mM \\ K_{i,NADH}^{MDH2}=3.18*10^{-3}mM \\ K_{i,ATP}^{MDH2}=183.2*10^{-3}mM \\ K_{i,ADP}^{MDH2}=394.4*10^{-3}mM \\ K_{i,AMP}^{MDH2}=420.0*10^{-3}mM \end{matrix}$

$$\begin{matrix} r_{MDH2}=\frac{V_{f,MDH2}\left(C_{NAD^{+}}^{m}C_{mal}^{m}-\frac{C_{OAA}^{m}C_{NADH}^{m}}{K_{eq,MDH2}}\right)}{\begin{matrix} K_{i,NAD^{+}}^{MDH2}K_{m,mal}^{MDH2}\alpha_{MDH2,i}+K_{m,mal}^{MDH2}C_{NAD^{+}}^{m}+K_{m,NAD^{+}}^{MDH2}\alpha_{MDH2,i}C_{mal}^{m}+C_{NAD^{+}}^{m}C_{mal}^{m}+\frac{K_{m,NAD^{+}}^{MDH2}C_{mal}^{m}C_{NADH}^{m}}{K_{i,NADH}^{MDH2}}+\frac{C_{NAD^{+}}^{m}C_{mal}^{m}C_{OAA}^{m}}{K_{i,OAA}^{MDH2}}+ \\ \left(\frac{K_{i,NAD^{+}}^{MDH2}K_{m,mal}^{MDH2}}{K_{i,NADH}^{MDH2}K_{m,OAA}^{MDH2}}\right)\left(K_{m,NADH}^{MDH2}\alpha_{MDH2,i}C_{OAA}^{m}+K_{m,OAA}^{MDH2}C_{NADH}^{m}+\frac{K_{mNADH}^{MDH2}}{K_{i,NAD^{+}}^{MDH2}}C_{NAD^{+}}^{m}C_{OAA}^{m}+C_{OAA}^{m}C_{NADH}^{m}+\frac{C_{mal}^{m}C_{OAA}^{m}C_{NADH}^{m}}{K_{i,mal}^{MDH2}}\right) \\ \end{matrix}} \\ \alpha_{MDH2,i}=\left(1+\frac{C_{ATP}^{m}P_{fATP}}{K_{i,ATP}^{MDH2}P_{ATP}}+\frac{C_{ADP}^{m}P_{fADP}}{K_{i,ADP}^{MDH2}P_{ADP}}+\frac{C_{AMP}^{m}P_{fAMP}}{K_{i,AMP}^{MDH2}P_{AMP}}\right) \\ \\ \\ \\ \\ \\ \end{matrix}$$

### NAD/NADH Shuttles

**Glutamate Oxaloacetate Transaminase 2 (GOT2)**

GOT2 is the isoform that is present in the mitochondria. The rate equation for GOT2 was taken from . The kinetics of GOT2 is modeled as ping pong bi bi mechanism. The kinetic constants were adopted from previous literature .

**Eq. S32:**

$\begin{matrix} V_{f,GOT}=3.87*10^{6} mMh^{-1} \\ K_{eq,GOT}=1.56 \\ K_{m,asp}^{GOT}=0.89 mM \\ K_{m,akg}^{GOT}=3.22 mM \\ K_{m,OAA}^{GOT}=88*10^{-3}mM \\ K_{m,glu}^{GOT}=32.5 mM \\ K_{i,asp}^{GOT}=3.9 mM \\ K_{i,akg}^{GOT}=0.73 mM \\ K_{i,OAA}^{GOT}=48*10^{-3}mM \\ K_{i,glu}^{GOT}=10.7 mM \\ K_{i,AKG}^{GOT}=26.5 mM \\ \\ \end{matrix}$

$$\begin{matrix} r_{GOT2}=\frac{V_{f,GOT2}\left(C_{asp}^{m}C_{akg}^{m}-\frac{C_{OAA}^{m}C_{glu}^{m}}{K_{eq,GOT2}}\right)}{\begin{matrix} K_{m,akg}^{GOT2}C_{asp}^{m}+K_{m,asp}^{GOT2}\alpha_{GOT2,i}C_{akg}^{m}+C_{asp}^{m}C_{akg}^{m}+\frac{K_{m,asp}^{GOT2}C_{akg}^{m}C_{glu}^{m}}{K_{i,glu}^{GOT2}}+ \\ \left(\frac{K_{i,asp}^{GOT2}K_{m,akg}^{GOT}}{K_{m,OAA}^{GOT2}K_{i,glu}^{GOT2}}\right)\left(\frac{K_{m,glu}^{GOT2}C_{asp}^{m}C_{OAA}^{m}}{K_{i,asp}^{GOT2}}+C_{OAA}^{m}C_{glu}^{m}+K_{m,glu}^{GOT2}\alpha_{GOT2,i}C_{OAA}^{m}+K_{m,OAA}^{GOT2}C_{glu}^{m}\right) \end{matrix}} \\ \\ \alpha_{GOT2,i}=\left(1+\frac{C_{akg}^{m}}{K_{i,akg}^{GOT2}}\right) \\ \end{matrix}$$

**Malate Dehydrogenase 1 (MDH1)**

MDH1 is the isoform that is present in the mitochondria. The rate equation and kinetic constants for MDH1 were taken from . The kinetics of MDH1 was modeled as ordered bi bi mechanism.

**Eq. S33:**

$$\begin{matrix} r_{MDH1}=\frac{V_{mf,MDH1}\left(C_{OAA}^{c}C_{NADH}^{c}-\frac{C_{NAD^{+}}^{c}C_{mal}^{c}}{K_{eq,MDH1}}\right)}{K_{i,NADH}^{MDH1}K_{m,OAA}^{MDH1}+K_{m,OAA}^{MDH1}C_{NADH}^{c}+K_{m,NADH}^{MDH1}C_{OAA}^{c}+C_{OAA}^{c}C_{NADH}^{c}+\frac{K_{m,NADH}^{MDH1}C_{OAA}^{c}C_{NAD^{+}}^{c}}{K_{i,NAD^{+}}^{MDH1}}+\frac{C_{OAA}^{c}C_{NADH}^{c}C_{mal}^{c}}{K_{i,mal}^{MDH1}}} \\ \begin{matrix} & & + \end{matrix}\frac{K_{i,NADH}^{MDH1}K_{m,OAA}^{MDH1}}{K_{i,NAD^{+}}^{MDH1}K_{m,mal}^{MDH1}}\left(K_{m,NAD^{+}}^{MDH1}C_{mal}^{c}+K_{m,mal}^{MDH1}C_{NAD^{+}}^{c}+\frac{K_{m,NAD^{+}}^{MDH1}C_{Mal}^{c}C_{NADH}^{c}}{K_{i,NADH}^{MDH1}}+C_{NAD^{+}}^{c}C_{Mal}^{c}+\frac{C_{Mal}^{c}C_{OAA}^{c}C_{NAD^{+}}^{c}}{K_{i,OAA}^{MDH1}}\right) \end{matrix}$$

$$\begin{matrix} V_{mf,MDH1}=3.59*10^{7}mM\text{ }h^{-1} \\ K_{eq,MDH1}=2.67*10^{4} \\ K_{m,NAD^{+}}^{MDH1}=0.114\text{ }mM \\ K_{m,mal}^{MDH1}=1.1\text{ }mM \\ K_{m,OAA}^{MDH1}=0.088\text{ }mM \\ K_{m,NADH}^{MDH1}=0.026\text{ }mM \\ K_{i,NADH}^{MDH1}=4.9*10^{-3}\text{ }mM \\ K_{i,OAA}^{MDH1}=63*10^{-3}\text{ }mM \\ K_{i,mal}^{MDH1}=7.1\text{ }mM \\ K_{i,NAD^{+}}^{MDH1}=0.94\text{ }mM \end{matrix}$$

**Glutamate Oxaloacetate Transaminase 1 (GOT1)**

GOT1 is the isoform that is present in the cytosol. The rate equation for GOT1 was taken from . The kinetics of GOT1 was modeled as ping pong bi bi mechanism. The kinetic constants were adopted from previous literature .

**Eq. S34:**

$\begin{matrix} V_{mf,GOT1}=2.36*10^{3} mMh^{-1} \\ K_{eq,GOT}=1.56 \\ K_{m,asp}^{GOT}=4.4mM \\ K_{m,akg}^{GOT}=0.38mM \\ K_{m,OAA}^{GOT}=95*10^{-3}mM \\ K_{m,glu}^{GOT}=9.6mM \\ K_{i,asp}^{GOT}=3.9mM \\ K_{i,akg}^{GOT}=0.73mM \\ K_{i,OAA}^{GOT}=48*10^{-3}mM \\ K_{i,glu}^{GOT}=8.4mM \\ K_{i,akg}^{GOT}=26.5mM \\ \\ \end{matrix}$

$$\begin{matrix} \\ r_{GOT1}=\frac{V_{mf,GOT1}\left(C_{asp}^{c}C_{akg}^{c}-\frac{C_{OAA}^{c}C_{glu}^{c}}{K_{eq,GOT1}}\right)}{\begin{matrix} K_{m,akg}^{GOT1}C_{asp}^{c}+K_{m,asp}^{GOT1}\alpha_{GOT1,i}C_{akg}^{c}+C_{asp}^{c}C_{akg}^{c}+\frac{K_{m,asp}^{GOT1}C_{akg}^{c}C_{glu}^{c}}{K_{i,glu}^{GOT1}}+ \\ \left(\frac{K_{i,asp}^{GOT1}K_{m,akg}^{GO1}}{K_{m,OAA}^{GOT1}K_{i,glu}^{GOT1}}\right)\left(\frac{K_{m,glu}^{GOT1}C_{asp}^{c}C_{OAA}^{c}}{K_{i,asp}^{GOT1}}+C_{OAA}^{c}C_{glu}^{c}+K_{m,glu}^{GOT1}\alpha_{GOT1,i}C_{OAA}^{c}+K_{m,OAA}^{GOT1}C_{glu}^{c}\right) \end{matrix}} \\ \\ \alpha_{GOT1,i}=\left(1+\frac{C_{akg}^{c}}{K_{i,akg}^{GOT1}}\right) \end{matrix}$$

**α-Ketoglutarate –Malate shuttle (AKGMAL)**

The rate equation for AKGMAL was taken from . The kinetics of AKGMAL was modeled as rapid equilibrium random bi bi mechanism. The kinetic constants were adopted from previous literature .

**Eq. S35:**

$$r_{AKGMAL}=\frac{V_{mf,AKGMAL}\left(C_{akg}^{c}C_{mal}^{m}-C_{akg}^{m}C_{mal}^{c}\right)}{K_{m,akgi}^{AKGMAL}K_{m,malx}^{AKGMAL}\left(2+\frac{C_{mal}^{c}}{K_{m,mali}^{AKGMAL}}+\frac{C_{mal}^{m}}{K_{m,malx}^{AKGMAL}}+\frac{C_{akg}^{c}}{K_{m,akgi}^{AKGMAL}}+\frac{C_{akg}^{m}}{K_{m,akgx}^{AKGMAL}}+\frac{C_{mal}^{c}C_{akg}^{m}}{K_{m,mali}^{AKGMAL}K_{m,akgx}^{AKGMAL}}+\frac{C_{mal}^{m}C_{akgi}^{c}}{K_{m,malx}^{AKGMAL}K_{m,akgi}^{AKGMAL}}\right)}$$

$$\begin{matrix} V_{mf,AKGMAL}=3.19*10^{6}mM\text{ }h^{-1} \\ K_{m,mali}^{AKGMAL}=0.4\text{ }mM \\ K_{m,malx}^{AKGMAL}=10\text{ }mM \\ K_{m,akgi}^{AKGMAL}=1.3\text{ }mM \\ K_{m,akgx}^{AKGMAL}=0.17\text{ }mM \end{matrix}$$

**Aspartate –Glutamate shuttle (ASPGLU)**

The rate equation for ASPGLU was taken from . The kinetics of ASPGLU was modeled as rapid equilibrium random bi bi with charge translocation mechanism. The kinetic constants were adopted from previous literature .

**Eq. S36:**

$$\begin{matrix} r_{ASPGLU}=\frac{V_{mf,ASPGLU}\left(K_{eq,ASPGLU}C_{asp}^{c}C_{glu}^{m}C_{H^{+}}^{m}-C_{asp}^{m}C_{glu}^{c}C_{H^{+}}^{c}\right)}{\begin{matrix} K_{eq,ASPGLU}K_{i,aspi}^{ASPGLU}K_{i,glux}^{ASPGLU}K_{H^{+}}^{ASPGLU}(2m+m\frac{C_{asp}^{c}}{K_{i,aspi}^{ASPGLU}}+\frac{C_{asp}^{c}C_{glu}^{m}C_{H^{+}}^{m}}{K_{i,aspi}^{ASPGLU}K_{i,glux}^{ASPGLU}K_{H^{+}}^{ASPGLU}}+m\frac{C_{asp}^{m}C_{H^{+}}^{c}}{K_{i,aspx}^{ASPGLU}K_{H^{+}}^{ASPGLU}}+m\frac{C_{asp}^{m}}{K_{i,aspx}^{ASPGLU}}+ \\ \frac{C_{asp}^{m}C_{glu}^{c}C_{H^{+}}^{c}}{K_{i,aspx}^{ASPGLU}K_{i,glui}^{ASPGLU}K_{H^{+}}^{ASPGLU}}+m\frac{C_{asp}^{c}C_{H^{+}}^{m}}{K_{i,aspi}^{ASP GLU}K_{H^{+}}^{ASPGLU}}+m\frac{C_{H^{+}}^{m}}{K_{H^{+}}^{ASPGLU}}+m\frac{C_{glu}^{c}C_{H^{+}}^{c}}{K_{i,glui}^{ASP GLU}K_{H^{+}}^{ASPGLU}}+m\frac{C_{H^{+}}^{c}}{K_{H^{+}}^{ASPGLU}}+m\frac{C_{glu}^{m}C_{H^{+}}^{m}}{K_{i,glux}^{ASP GLU}K_{H^{+}}^{ASPGLU}}) \end{matrix}} \\ \end{matrix}$$

$$\begin{matrix} V_{mf,ASPGLU}=2.49*10^{4}mM\text{ }h^{-1} \\ K_{eq,ASPGLU}=0.6 \\ K_{i,aspi}^{ASPGLU}=0.028\text{ }mM \\ K_{i,aspx}^{ASPGLU}=2.8\text{ }mM \\ K_{i,glui}^{ASPGLU}=0.18\text{ }mM \\ K_{i,glux}^{ASPGLU}=1.6\text{ }mM \\ K_{H^{+}}^{ASPGLU}=10^{-6.5}mM \\ m = 1.8 \end{matrix}$$

### Transporters

**Glucose Transporter (GLUT)**: Glucose transporters mediate transport of glucose across plasma membranes. Till date, fourteen glucose transporters (isozymes) have been identified which perform the same function but have very different kinetic properties . Kinetics of the GLUT1 isozyme was considered in the model and was modeled as uni uni steady state kinetics.

**Eq. S37:**

$$r_{GlcTr}=\frac{V_{mf}^{GlcTr}\frac{C_{glc}^{e}}{K_{glc}^{GlcTr}}-V_{mr}^{GlcTr}\frac{C_{glc}^{c}}{K_{glc}^{GlcTr}}}{1+\frac{C_{glc}^{e}}{K_{glc}^{GlcTr}}+\frac{C_{glc}^{c}}{K_{glc}^{GlcTr}}}$$

$$\begin{matrix} V_{mf}^{GlcTr}=7.67\text{ mM}h^{-1} \\ V_{mr}^{GlcTr}=0.767\text{ mM}h^{-1} \\ K_{glc}^{GlcTr}=1.50\text{ }mM \end{matrix}$$

**Pyruvate –Hydrogen shuttle (PYRH)**

PYRH was modeled as reversible mass action kinetics. The rate equation was taken from [75].

**Eq. S38:**

$$r_{PYRH}=V_{m,PYRH}\left(C_{pyr}^{c}C_{H^{+}}^{c}-C_{pyr}^{m}C_{H^{+}}^{m}\right)$$

$$V_{m,PYRH}\text{=1*10}^{13}\text{ }mM\text{ }h^{-1}$$

**Citrate –Malate shuttle (CITMAL)**

CITMAL was modeled as reversible mass action kinetics. The rate equation was taken from [75].

**Eq. S39:**

$$r_{CITMAL}=V_{m,CITMAL}\left(C_{cit}^{c}C_{mal}^{m}-C_{cit}^{m}C_{mal}^{c}\right)$$

$$V_{m,CITMAL}=296.6 mM\text{ }h^{-1}$$

**Malate-Phosphate shuttle (MALPi)**

MALPi was modeled as reversible mass action kinetics. The rate equation was taken from [75].

**Eq. S40:**

$$r_{MALPi}=V_{m,MALPi}\left(C_{mal}^{c}C_{Pi}^{m}-C_{mal}^{m}C_{Pi}^{c}\right)$$

$$V_{m,MALPi}=17.3 mM\text{ }h^{-1}$$

**Glutamate-Hydrogen shuttle (GLUH)**

GLUH was modeled as reversible mass action kinetics. The rate equation was taken from [75].

**Eq. S41:**

$$r_{GLUH}=V_{m,GLUH}\left(C_{glu}^{c}C_{H^{+}}^{c}-C_{glu}^{m}C_{H^{+}}^{m}\right)$$

$$V_{m,GLUH}=3.87*10^{8} mM\text{ }h^{-1}$$

### Other Reactions

**Glutaminase (GLS)**

The kinetics of GLS was modeled as simple Michaelis-Menten kinetics with non-competitive inhibition by glutamate. The kinetic constants for GLS were taken from .

**Eq. S42:**

$$r_{GLS}=\frac{V_{m,GLS}\left(C_{gln}^{c}-\frac{C_{glu}^{m}}{K_{eq,GLS}}\right)}{K_{m,gln}^{GLS}\left(1+\frac{C_{glu}^{m}}{K_{i,glu}^{GLS}}\right)+C_{gln}^{c}}$$

$$\begin{matrix} V_{m,GLS}=38.8 mM\text{ }h^{-1} \\ K_{eq,GLS}=1 \\ K_{m,gln}^{GLS}=12 mM \\ K_{i,glu}^{GLS}=55 mM \end{matrix}$$

**Glutamate Dehydrogenase (GDH)**

The kinetic constants for GDH were taken from . The kinetics of GDH was modeled as random bi bi mechanism.

**Eq. S43:**

$$\begin{matrix} r_{GDH}=\frac{V_{f,GDH}\left(C_{NAD}^{m}C_{Glu}^{m}-\frac{C_{AKG}^{m}C_{NADH}^{m}C_{NH3}^{m}}{K_{eq,GDH}}\right)}{K_{i,NAD}^{GDH}K_{Glu}^{GDH}+K_{m,Glu}^{GDH}C_{NAD}^{m}+K_{m,NAD}^{GDH}C_{Glu}^{m}+C_{Glu}^{m}C_{NAD}^{m}+\frac{C_{Glu}^{m}C_{NAD}^{m}C_{NH3}^{m}}{K_{i,AKG}^{GDH}}+\frac{K_{i,NAD}^{GDH}K_{m,Glu}^{GDH}C_{NADH}^{m}}{K_{i,NADH}^{GDH}}} \\ \begin{matrix} & & +\frac{K_{m,Glu}^{GDH}C_{NAD}^{m}C_{NH3}^{m}}{K_{i,NH3}^{GDH}}+ \end{matrix}\frac{K_{i,NAD}^{GDH}K_{m,Glu}^{GDH}K_{m,NADH}^{GDH}C_{AKG}^{m}C_{NH3}^{m}}{K_{m,NH3}^{GDH}K_{i,AKG}^{GDH}K_{i,NADH}^{GDH}}+\frac{K_{m,NAD}^{GDH}C_{Glu}^{m}C_{NADH}^{m}}{K_{i,NADH}^{GDH}}+\frac{C_{Glu}^{m}C_{AKG}^{m}C_{NAD}^{m}C_{NH3}^{m}}{K_{i,NH3}^{GDH}K_{i,AKG}^{GDH}} \\ \begin{matrix} & & + \end{matrix}\frac{K_{i,NAD}^{GDH}K_{m,Glu}^{GDH}K_{m,AKG}^{GDH}C_{NADH}^{m}C_{NH3}^{m}}{K_{m,NH3}^{GDH}K_{i,AKG}^{GDH}K_{i,NADH}^{GDH}}+\frac{K_{i,NAD}^{GDH}K_{m,Glu}^{GDH}K_{m,AKG}^{GDH}C_{NH3}^{m}C_{AKG}^{m}C_{NADH}^{m}}{K_{m,NH3}^{GDH}K_{i,AKG}^{GDH}K_{i,NADH}^{GDH}}+\frac{C_{Glu}^{m}C_{NAD}^{m}C_{AKG}^{m}}{K_{i,AKG}^{GDH}} \\ \begin{matrix} & & +\frac{K_{i,NAD}^{GDH}K_{m,Glu}^{GDH}K_{m,AKG}^{GDH}C_{NH3}^{m}}{K_{m,NH3}^{GDH}K_{i,AKG}^{GDH}} \end{matrix}+\frac{K_{i,NAD}^{GDH}K_{m,Glu}^{GDH}C_{Glu}^{m}C_{NADH}^{m}C_{AKG}^{m}}{K_{i,Glu}^{GDH}K_{i,AKG}^{GDH}K_{i,NADH}^{GDH}}+\frac{K_{i,NAD}^{GDH}K_{m,Glu}^{GDH}C_{AKG}^{m}C_{NADH}^{m}}{K_{i,AKG}^{GDH}K_{i,NADH}^{GDH}} \\ \begin{matrix} & & +\frac{K_{m,NADH}^{GDH}K_{m,Glu}^{GDH}C_{NH3}^{m}C_{AKG}^{m}C_{NAD}^{m}}{K_{m,NH3}^{GDH}K_{i,AKG}^{GDH}K_{i,NADH}^{GDH}}+ \end{matrix}\frac{K_{i,NAD}^{GDH}K_{m,Glu}^{GDH}C_{Glu}^{m}C_{AKG}^{m}C_{NADH}^{m}C_{NH3}^{m}}{K_{m,NH3}^{GDH}K_{i,Glu}^{GDH}K_{i,AKG}^{GDH}K_{i,NADH}^{GDH}} \end{matrix}$$

$$\begin{matrix} V_{f,GDH}=5.55*10^{3}mM^{-1}h^{-1} \\ K_{m,Glu}^{GDH}=3.5\text{ }mM \\ K_{m,NADH}^{GDH}=0.04\text{ }mM \\ K_{m,AKG}^{GDH}=1.1\text{ }mM \\ K_{m,NH3}^{GDH}=6\text{ }mM \\ K_{i,AKG}^{GDH}=0.25\text{ }mM \\ K_{i,NH3}^{GDH}=6\text{ }mM \\ K_{i,Glu}^{GDH}=3.5\text{ }mM \\ K_{i,NADH}^{GDH}=0.004\text{ }mM \\ K_{i,NAD}^{GDH}=1\text{ }mM \\ K_{eq,GDH}=0.003 \\ \\ \end{matrix}$$

**ATP-Citrate Lyase (CLY)**

The kinetics of CLY was modeled as ordered bi bi mechanism. The kinetic constants were adopted from previous literature .

**Eq. S44:**

$$\begin{matrix} r_{CLY}=\frac{V_{m,CLY}C_{cit}^{c}C_{CoASH}^{c}}{K_{i,cit}^{CLY}K_{m,CoASH}^{CLY}+K_{m,cit}^{CLY}C_{CoASH}^{c}+K_{m,AcCoA}^{CLY}C_{cit}^{c}+C_{cit}^{c}C_{CoASH}^{c}+\frac{K_{m,cit}^{CLY}C_{CoASH}^{c}C_{AcCoA}^{c}}{K_{i,AcCoA}^{CLY}}+\frac{C_{cit}^{c}C_{CoASH}^{c}C_{OAA}^{c}}{K_{i,OAA}^{CLY}}} \\ \begin{matrix} & & + \end{matrix}\frac{K_{i,cit}^{CLY}K_{m,CoASH}^{CLY}}{K_{m,OAA}^{CLY}K_{i,AcCoA}^{CLY}}\left(K_{m,AcCoA}^{CLY}C_{OAA}^{c}+K_{m,OAA}^{CLY}C_{AcCoA}^{c}+\frac{K_{m,AcCoA}^{CLY}C_{cit}^{c}C_{OAA}^{c}}{K_{i,cit}^{CLY}}+C_{OAA}^{c}C_{AcCoA}^{c}+\frac{C_{CoASH}^{c}C_{OAA}^{c}C_{AcCoA}^{c}}{K_{i,CoASH}^{CLY}}\right) \end{matrix}$$

$$\begin{matrix} V_{m,CLY}=17.5\text{ }mM^{-1}h^{-1} \\ K_{m,cit}^{CLY}=0.0493\text{ }mM \\ K_{i,cit}^{CLY}=0.0475\text{ }mM \\ K_{m,CoASH}^{CLY}=4.4*10^{-3}\text{ }mM \\ K_{i,CoASH}^{CLY}=6.1*10^{-3}\text{ }mM \\ K_{m,OAA}^{CLY}=0.177\text{ }mM \\ K_{i,OAA}^{CLY}=0.177\text{ }mM \\ K_{m,AcCoA}^{CLY}=9.8*10^{-3}\text{ }mM \\ K_{i,AcCoA}^{CLY}=9.8*10^{-3}\text{ }mM \end{matrix}$$

**Mitochondrial Malic Enzyme (MMALIC)**

The rate equation and kinetic constants for CMALIC were adopted from . The kinetics of CMALIC was modeled as ordered bi ter mechanism.

**Eq. S45:**

$$r_{mmalic}=\frac{V_{mmalic}\left(C_{mal}^{m}C_{NAD^{+}}^{m}-\frac{C_{pyr}^{m}C_{NADH}^{m}C_{CO_{2}}^{m}}{K_{eq,mmalic}}\right)}{K_{mal}^{mmalic}C_{NAD^{+}}^{m}\left(1+\frac{C_{ATP}^{m}}{K_{i,ATP}^{mmalic}}\right)+K_{NAD^{+}}^{mmalic}C_{mal}^{m}+K_{mal}^{mmalic}K_{NAD^{+}}^{mmalic}}$$

$$\begin{matrix} V_{mmalic}=46.3\text{ }mM^{-1}h^{-1} \\ K_{eq,mmalic}=34.4 \\ K_{mal}^{mmalic}=1.7\text{ }mM \\ K_{NAD}^{mmalic}=0.16\text{ }mM \\ K_{i,ATP}^{mmalic}=0.5\text{ }mM \end{matrix}$$

**Cytosolic Malic Enzyme (CMALIC)**

The rate equation and kinetic constants for CMALIC were taken from . The kinetics of CMALIC was modeled as ordered bi ter mechanism.

**Eq. S46:**

$$\begin{matrix} r_{cmalic}=\frac{V_{f,cmalic}\left(C_{mal}^{c}C_{NADP}^{c}-\frac{C_{CO_{2}}^{c}C_{pyr}^{c}C_{NADPH}^{c}}{K_{eq,cmalic}}\right)}{K_{i,NADP}^{cmalic}K_{m,mal}^{cmalic}+K_{m,mal}^{cmalic}C_{NADP}^{c}+K_{m,NADP}^{cmalic}C_{mal}^{c}+C_{mal}^{c}C_{NADP}^{c}+\frac{K_{i,NADP}^{cmalic}K_{m,mal}^{cmalic}K_{m,pyr}^{cmalic}C_{CO_{2}}^{c}}{K_{m,CO_{2}}^{cmalic}K_{i,pyr}^{cmalic}}} \\ \begin{matrix} & & +\frac{K_{i,NADP}^{cmalic}K_{m,mal}^{cmalic}C_{NADPH}^{c}}{K_{i,NADPH}^{cmalic}}+ \end{matrix}\frac{K_{m,mal}^{cmalic}K_{m,NADPH}^{cmalic}C_{NADP}^{c}C_{pyr}^{c}}{K_{i,pyr}^{cmalic}K_{i,NADPH}^{cmalic}}\left(\frac{C_{CO_{2}}^{c}}{K_{m,CO_{2}}^{cmalic}}+\frac{K_{i,CO_{2}}^{cmalic}C_{mal}^{c}}{K_{i,mal}^{cmalic}K_{m,CO_{2}}^{cmalic}}+\frac{C_{mal}^{c}C_{CO_{2}}^{c}}{K_{i,mal}^{cmalic}K_{m,CO_{2}}^{cmalic}}\right) \\ \begin{matrix} & & + \end{matrix}\frac{K_{m,NADP}^{cmalic}C_{mal}^{c}C_{NADPH}^{c}}{K_{i,NADPH}^{cmalic}}\left(1+\frac{C_{pyr}^{c}}{K_{i,pyr}^{cmalic}}+\frac{C_{CO_{2}}^{c}C_{pyr}^{c}}{K_{i,CO_{2}}^{cmalic}K_{i,pyr}^{cmalic}}\right)+\frac{K_{m,mal}^{cmalic}K_{i,NADP}^{cmalic}K_{m,pyr}^{cmalic}C_{NADPH}^{c}}{K_{i,pyr}^{cmalic}K_{i,NADPH}^{cmalic}}\left(\frac{C_{pyr}^{c}}{K_{m,pyr}^{cmalic}}+\frac{C_{CO_{2}}^{c}}{K_{m,CO_{2}}^{cmalic}}\right) \\ \begin{matrix} & & + \end{matrix}\frac{K_{m,mal}^{cmalic}K_{i,NADP}^{cmalic}K_{m,NADPH}^{cmalic}C_{CO_{2}}^{c}C_{pyr}^{c}}{K_{m,CO_{2}}^{cmalic}K_{i,pyr}^{cmalic}K_{i,NADPH}^{cmalic}}\left(1+\frac{C_{NADPH}^{c}}{K_{m,NADPH}^{cmalic}}\right)+\frac{K_{m,mal}^{cmalic}K_{m,pyr}^{cmalic}C_{NADP}^{c}C_{CO_{2}}^{c}}{K_{m,CO_{2}}^{cmalic}K_{i,pyr}^{cmalic}}\left(1+\frac{C_{mal}^{c}}{K_{i,mal}^{cmalic}}\right) \end{matrix}$$

$$\begin{matrix} V_{f,cmalic}=174\text{ }mM^{-1}h^{-1} \\ K_{m,mal}^{cmalic}=120*10^{-3}\text{ }mM \\ K_{m,NADP}^{cmalic}=1.4*10^{-3}\text{ }mM \\ K_{m,CO_{2}}^{cmalic}=13*10^{-3}\text{ }mM \\ K_{m,pyr}^{cmalic}=6.4*10^{-3}\text{ }mM \\ K_{m,NADPH}^{cmalic}=2.1*10^{-3}mM \\ K_{i,NADP}^{cmalic}=9.6*10^{-4}\text{ }mM \\ K_{i,mal}^{cmalic}=0.22\text{ }mM \\ K_{i,CO_{2}}^{cmalic}=11.7*10^{-3}\text{ }mM \\ K_{i,pyr}^{cmalic}=7.8\text{ }mM \\ K_{i,NADPH}^{cmalic}=2*10^{-3}\text{ }mM \\ K_{eq,cmalic}=34.4 \end{matrix}$$

**Glutamate Alanine Transaminase (GPT)**

The rate equation and kinetic constants for GPT were taken from . The kinetics of GPT was modeled as ping pong bi bi mechanism.

**Eq. S47:**

$$\begin{matrix} r_{GPT}=\frac{V_{f,GPT}\left(C_{Ala}^{m}C_{AKG}^{m}-\frac{C_{Pyr}^{m}C_{Glu}^{m}}{K_{eq,GPT}}\right)}{K_{Ala}^{GPT}C_{AKG}^{m}+K_{AKG}^{GPT}C_{Ala}^{m}+C_{AKG}^{m}C_{Ala}^{m}+\frac{K_{Ala}^{GPT}C_{AKG}^{m}C_{Glu}^{m}}{K_{i,Glu}^{GPT}}+\frac{K_{AKG}^{GPT}C_{Ala}^{m}C_{Ala}^{m}}{K_{IA}^{GPT}}+\frac{K_{AKG}^{GPT}C_{Ala}^{m}C_{Glu}^{m}}{K_{RG}^{GPT}}} \\ \begin{matrix} & & + \end{matrix}\frac{V_{f,GPT}}{V_{r,GPT}K_{eq,GPT}}\left(K_{Pyr}^{GPT}C_{Glu}^{m}+K_{Glu}^{GPT}C_{Pyr}^{m}+C_{Pyr}^{m}C_{Glu}^{m}+\frac{K_{AKG}^{GPT}C_{Ala}^{m}C_{Pyr}^{m}}{K_{i,Pyr}^{GPT}}+\frac{K_{Pyr}^{GPT}C_{Glu}^{m}C_{Glu}^{m}}{K_{IG}^{GPT}}\right) \end{matrix}$$

$$\begin{matrix} V_{f,GPT}=66.4\text{ }mM^{-1}h^{-1} \\ V_{r,GPT}=3.97*10^{7}\text{ }mM^{-1}h^{-1} \\ K_{Ala}^{GPT}=3\text{ }mM \\ K_{AKG}^{GPT}=0.12\text{ }mM \\ K_{Pyr}^{GPT}=0.23\text{ }mM \\ K_{Glu}^{GPT}=8.1\text{ }mM \\ K_{i,Pyr}^{GPT}=0.23\text{ }mM \\ K_{i,Glu}^{GPT}=2.8\text{ }mM \\ K_{IA}^{GPT}=470\text{ }mM \\ K_{IG}^{GPT}=96\text{ }mM \\ K_{RG}^{GPT}=79.16\text{ }mM \\ K_{eq,GPT}=2.2 \end{matrix}$$

**Pyruvate Carboxylase (PC)**

The kinetics of PC was modeled as bi uni mechanism. The kinetic constants for PC were taken from .

**Eq. S48:**

$$r_{PC}=\frac{V_{PC}\left(C_{pyr}^{m}C_{CO_{2}}^{m}-\frac{C_{OAA}^{m}}{K_{eq,PC}}\right)}{K_{m,pyr}^{PC}K_{m,CO_{2}}^{PC}+K_{m,pyr}^{PC}C_{CO_{2}}^{m}+K_{m,CO_{2}}^{PC}C_{pyr}^{m}+C_{pyr}^{m}C_{CO_{2}}^{m}}$$

$$\begin{matrix} V_{PC}=718.2\text{ }mM^{-1}h^{-1} \\ K_{m,pyr}^{PC}=0.22\text{ }mM \\ K_{m,CO_{2}}^{PC}=3.2\text{ }mM \\ K_{eq,PC}=1 \end{matrix}$$

**Mono Carboxylate Transporter (MCT)**

The kinetics of MCT was modeled as ordered bi bi mechanism. The kinetic constants for MCT were adopted from .

**Eq. S49:**

$$\begin{matrix} r_{MCT}=\frac{V_{m,MCT}\left(C_{H^{+}}^{c}C_{lac}^{c}-C_{H^{+}}^{e}C_{lac}^{e}\right)}{K_{m,laci}^{MCT}K_{i,H^{+}}^{MCT}+K_{m,H^{+}}^{MCT}C_{lac}^{c}+K_{m,laci}^{MCT}C_{H^{+}}^{c}+K_{m,H^{+}}^{MCT}C_{lac}^{e}+K_{m,laci}^{MCT}C_{H^{+}}^{e}+C_{H^{+}}^{c}C_{lac}^{c}} \\ \begin{matrix} & & C_{H^{+}}^{e}C_{lac}^{e}+ \end{matrix}\frac{K_{m,H^{+}}^{MCT}C_{lac}^{c}C_{H^{+}}^{e}}{K_{i,H^{+}}^{MCT}}+\frac{K_{m,H^{+}}^{MCT}C_{lac}^{e}C_{H^{+}}^{c}}{K_{i,H^{+}}^{MCT}}+\frac{C_{lac}^{c}C_{H^{+}}^{c}C_{H^{+}}^{e}}{K_{ii,H^{+}}^{MCT}}+\frac{C_{lac}^{e}C_{H^{+}}^{c}C_{H^{+}}^{e}}{K_{ii,H^{+}}^{MCT}} \end{matrix}$$

$$\begin{matrix} V_{MCT}=2.73*10^{3}\text{ }mM^{-1}h^{-1} \\ K_{m,H^{+}}^{MCT}=10^{-4}\text{ }mM \\ K_{m,laci}^{MCT}=2.5\text{ }mM \\ K_{i,H^{+}}^{MCT}=2*10^{-4}\text{ }mM \\ K_{ii,H^{+}}^{MCT}=2*10^{-4}\text{ }mM \\ \end{matrix}$$

| $P_{ATP}=1+\frac{C_{H^{+}}^{m}}{K_{HATP}}+\frac{C_{Mg}^{m}}{K_{MgATP}}+\frac{C_{K}^{m}}{K_{KATP}}$ $P_{ADP}=1+\frac{C_{H^{+}}^{m}}{K_{HADP}}+\frac{C_{Mg}^{m}}{K_{MgADP}}+\frac{C_{K}^{m}}{K_{KADP}}$ $P_{AMP}=1+\frac{C_{H^{+}}^{m}}{K_{HAMP}}+\frac{C_{Mg}^{m}}{K_{MgAMP}}+\frac{C_{K}^{m}}{K_{KAMP}}$ $P_{GTP}=P_{ATP}$ $P_{GDP}=P_{ADP}$ $P_{CoASH}=1+\frac{C_{H^{+}}^{m}}{K_{HCoASH}}$ $P_{cit}=1+\frac{C_{H^{+}}^{m}}{K_{Hcit}}+\frac{C_{Mg}^{m}}{K_{Mgcit}}+\frac{C_{K}^{m}}{K_{Kcit}}$ $P_{scoa}=1+\frac{C_{H^{+}}^{m}}{K_{Hscoa}}$ $P_{fATP}=1+\frac{C_{H^{+}}^{m}}{K_{HATP}}+\frac{C_{K^{+}}^{m}}{K_{KATP}}$ $P_{fADP}=1+\frac{C_{H^{+}}^{m}}{K_{HADP}}+\frac{C_{K^{+}}^{m}}{K_{KADP}}$ $P_{fAMP}=1+\frac{C_{H^{+}}^{m}}{K_{HAMP}}+\frac{C_{K^{+}}^{m}}{K_{KAMP}}$ $P_{fGTP}=P_{fATP}$ $P_{fGDP}=P_{fGDP}$ | $\begin{matrix} K_{HATP}=2.57*10^{-7}M \\ K_{MgATP}=1.51*10^{-4}M \\ K_{KATP}=1.35*10^{-2}M \end{matrix}$ $\begin{matrix} K_{HADP}=3.8*10^{-7}M \\ K_{MgADP}=1.62*10^{-3}M \\ K_{KADP}=2.95*10^{-2}M \end{matrix}$ $\begin{matrix} K_{HAMP}=6.03*10^{-7}M \\ K_{MgAMP}=1.38*10^{-2}M \\ K_{KAMP}=8.91*10^{-2}M \end{matrix}$ $K_{HCoASH}=7.41*10^{-9}M$ $\begin{matrix} K_{Hcit}=2.34*10^{-6}M \\ K_{Mgcit}=4.27*10^{-4}M \\ K_{Kcit}=4.58*10^{-1}M \end{matrix}$ $K_{Hscoa}=1.1*10^{-4}M$ |
| --- | --- |

# Differential Equations

## Glycolysis

1. Glucose:$\frac{dC_{glc}^{c}}{dt}=r_{GlcTr}-r_{HK}$
2. Glucose 6-phosphate: $\frac{dC_{g6p}^{c}}{dt}=r_{HK}-r_{GPI}-r_{G6PD}$
3. Fructose 6-phosphate: $\frac{dC_{f6p}^{c}}{dt}=r_{PGI}+r_{TA}+r_{TK2}-r_{PFK2}+r_{F2,6BPase}-r_{PFK}$
4. Fructose 1,6-bisphosphate: $\frac{dC_{f16bp}^{c}}{dt}=r_{PFK}-r_{ALD}$
5. Fructose 2,6-bisphosphate: $\frac{dC_{f26bp}^{c}}{dt}=r_{PFK2}-r_{F2,6BPase}$
6. Dihydroxyacetone phosphate: $\frac{dC_{dhap}^{c}}{dt}=r_{ALD}-r_{TPI}$
7. Glyceraldehyde 3-phosphate: $\frac{dC_{gap}^{c}}{dt}=r_{ALD}+r_{TPI}+r_{TK1}+r_{TK2}-r_{TA}-r_{GAPDH}$
8. 1,3-bisphosphoglycerate: $\frac{dC_{1,3bpg}^{c}}{dt}=r_{GAPDH}-r_{PGK}$
9. 3-phosphoglycerate: $\frac{dC_{3pg}^{c}}{dt}=r_{PGK}-r_{PGM}$
10. 2-phosphoglycerate: $\frac{dC_{2pg}^{c}}{dt}=r_{PGM}-r_{EN}$
11. Phosphoenolpyruvate: $\frac{dC_{pep}^{c}}{dt}=r_{EN}-r_{PK}$
12. Pyruvate: $\frac{dC_{pyr}^{c}}{dt}=r_{PK}-r_{LDH}-r_{PYRH}+r_{CMALIC}$
13. Lactate: $\frac{dC_{lac}^{c}}{dt}=r_{LDH}-r_{MCT}$

## Pentose Phosphate Pathway

1. 6-phosphogluconate: $\frac{dC_{6pg}^{c}}{dt}=r_{G6PD}-r_{6PGD}$
2. Ribulose 5-phosphate: $\frac{dC_{ru5p}^{c}}{dt}=r_{6PGD}-r_{RPE}-r_{RPI}$
3. xylulose 5-phosphate: $\frac{dC_{xyl5p}^{c}}{dt}=r_{RPE}-r_{TK1}-r_{TK2}$
4. ribose 5-phosphate: $\frac{dC_{r5p}^{c}}{dt}=r_{RPI}-r_{PRPPS}-r_{TK1}$
5. Erythrose 4-phospahte**:** $\frac{dC_{e4p}^{c}}{dt}=r_{TA}-r_{TK2}$
6. Sedoheptulose 7-phosphate: $\frac{dC_{s7p}^{c}}{dt}=r_{TK1}-r_{TA}$
7. Glutathione: $\frac{dC_{glutathione}^{c}}{dt}=r_{GSSGR}-r_{GSHOX}$

## TCA Cycle

1. Mitochondrial pyruvate: $\frac{dC_{pyr}^{m}}{dt}=r_{PYRH}*\frac{V_{c}}{V_{m}}-r_{PDH}+r_{mmalic}-r_{PC}+r_{GPT1}$
2. Mitochondrial Acetyl-CoA: $\frac{dC_{AcCoA}^{m}}{dt}=r_{PDH}-r_{CS}$
3. Mitochondrial Citrate: $\frac{dC_{cit}^{m}}{dt}=r_{CS}-r_{ACON}+r_{CITMAL}$
4. Mitochondrial Isocitrate: $\frac{dC_{icit}^{m}}{dt}=r_{ACON}-r_{IDH}$
5. Mitochondrial Alpha-ketoglutarate: $\frac{dC_{akg}^{m}}{dt}=r_{IDH}-r_{AKGD}+r_{AKGMAL}-r_{GOT2}+r_{GDH}-r_{GPT1}$
6. Mitochondrial Succinyl-CoA: $\frac{dC_{SCoA}^{m}}{dt}=r_{AKGD}-r_{SCOAS}$
7. Mitochondrial Succinate: $\frac{dC_{suc}^{m}}{dt}=r_{SCOAS}-r_{SDH}$
8. Mitochondrial Fumarate: $\frac{dC_{fum}^{m}}{dt}=r_{SDH}-r_{FUM}$
9. Mitochondrial Malate: $\frac{dC_{mal}^{m}}{dt}=r_{FUM}-r_{mmalic}-r_{MDH2}-r_{AKGMAL}-r_{CITMAL}+r_{MALPi}$
10. Mitochondrial Oxaloacetate: $\frac{dC_{OAA}^{m}}{dt}=-r_{CS}+r_{MDH2}+r_{GOT2}+r_{PC}$

## NAD/NADH Shuttles

1. Mitochondrial Aspartate: $\frac{dC_{asp}^{m}}{dt}=r_{ASPGLU}-r_{GOT2}$
2. Mitochondrial Glutamate: $\frac{dC_{glu}^{m}}{dt}=r_{GOT2}-r_{ASPGLU}-r_{GDH}-r_{GLUH}+r_{GPT1}$
3. Aspartate: $\frac{dC_{asp}^{c}}{dt}=-r_{GOT1}-r_{ASPGLU}*\frac{V_{m}}{V_{c}}$
4. Glutamate: $\frac{dC_{glu}^{c}}{dt}=r_{GOT1}+r_{ASPGLU}*\frac{V_{m}}{V_{c}}-r_{GLUH}*\frac{V_{m}}{V_{c}}+r_{GLS}$
5. Oxaloacetate: $\frac{dC_{OAA}^{c}}{dt}=r_{GOT1}-r_{MDH1}+r_{CLY}$
6. Malate: $\frac{dC_{mal}^{c}}{dt}=r_{MDH1}+r_{AKGMAL}*\frac{V_{m}}{V_{c}}+r_{CITMAL}*\frac{V_{m}}{V_{c}}-r_{MALPi}*\frac{V_{m}}{V_{c}}-r_{cmalic}$
7. Alpha-ketoglutarate: $\frac{dC_{akg}^{c}}{dt}=-r_{AKGMAL}*\frac{V_{m}}{V_{c}}-r_{GOT1}$

## Other Equations

1. Citrate: $\frac{dC_{cit}^{c}}{dt}=-r_{CITMAL}*\frac{V_{m}}{V_{c}}-r_{CLY}$
2. NAD: $\frac{dC_{NAD}^{c}}{dt}=r_{LDH}-r_{GAPDH}+r_{MDH1}$
3. NADP: $\frac{dC_{NADP}^{c}}{dt}=r_{GSSGR}-r_{G6PD}-r_{6PGD}-r_{cmalic}$

# Stability Analysis

For a system of ordinary differential equation $\frac{dx}{dt}=f\left(x\right)$, where $x\in R^{n}$and $f:R^{n}\to R^{n}$, the Jacobian matrix, $J$ is the $n\times n$matrix defined by $J_{ij}=\partial f_{i}/\partial x_{j}$. The local stability of a steady state was investigated using the standard approach of calculating the eigenvalues of the Jacobian evaluated at the steady state. If all the eigenvalues have negative real part, the steady state is stable, if not it is unstable. The Jacobian matrix was calculated as part of the output of Matlab’s *fsolve* function. The eigenvalues of the Jacobian matrix were evaluated using Matlab’s *eig* function.

# References

1. Mulquiney PJ, Kuchel PW (1999) Model of 2,3-bisphosphoglycerate metabolism in the human erythrocyte based on detailed enzyme kinetic equations: equations and parameter refinement. Biochem J 342 Pt 3: 581-596.

2. Fornaini G, Magnani M, Fazi A, Accorsi A, Stocchi V, et al. (1985) Regulatory properties of human erythrocyte hexokinase during cell ageing. Arch Biochem Biophys 239: 352-358.

3. Gerber G, Preissler H, Heinrich R, Rapoport SM (1974) Hexokinase of human erythrocytes. Purification, kinetic model and its application to the conditions in the cell. Eur J Biochem 45: 39-52.

4. Magnani M, Stocchi V, Ninfali P, Dacha M, Fornaini G (1980) Action of oxidized and reduced glutathione on rabbit red blood cell hexokinase. Biochim Biophys Acta 615: 113-120.

5. Rijksen G, Jansen G, Kraaijenhagen RJ, Van der Vlist MJ, Vlug AM, et al. (1981) Separation and characterization of hexokinase I subtypes from human erythrocytes. Biochim Biophys Acta 659: 292-301.

6. Rijksen G, Staal GE (1977) Regulation of human erythrocyte hexokinase. The influence of glycolytic intermediates and inorganic phosphate. Biochim Biophys Acta 485: 75-86.

7. Gracy RW, Tilley BE (1975) Phosphoglucose isomerase of human erythrocytes and cardiac tissue. Methods Enzymol 41: 392-400.

8. Kahana SE, Lowry OH, Schulz DW, Passonneau JV, Crawford EJ (1960) The kinetics of phosphoglucoisomerase. J Biol Chem 235: 2178-2184.

9. Tilley BE, Gracy RW, Welch SG (1974) A point mutation increasing the stability of human phosphoglucose isomerase. J Biol Chem 249: 4751-4759.

10. Dunaway GA, Kasten TP, Sebo T, Trapp R (1988) Analysis of the phosphofructokinase subunits and isoenzymes in human tissues. Biochem J 251: 677-683.

11. Hanson RL, Rudolph FB, Lardy HA (1973) Rabbit muscle phosphofructokinase. The kinetic mechanism of action and the equilibrium constant. J Biol Chem 248: 7852-7859.

12. Merry S, Britton HG (1985) The mechanism of rabbit muscle phosphofructokinase at pH8. Biochem J 226: 13-28.

13. Otto M, Heinrich R, Jacobasch G, Rapoport S (1977) A mathematical model for the influence of anionic effectors on the phosphofructokinase from rat erythrocytes. Eur J Biochem 74: 413-420.

14. Otto M, Heinrich R, Kuhn B, Jacobasch G (1974) A mathematical model for the influence of fructose 6-phosphate, ATP, potassium, ammonium and magnesium on the phosphofructokinase from rat erythrocytes. Eur J Biochem 49: 169-178.

15. Kitajima S, Sakakibara R, Uyeda K (1984) Kinetic studies of fructose 6-phosphate,2-kinase and fructose 2,6-bisphosphatase. J Biol Chem 259: 6896-6903.

16. Kretschmer M, Schellenberger W, Hofmann E (1985) Quasi-stationary concentrations of fructose-2,6-bisphosphate in the phosphofructokinase-2/fructose-2,6-bisphosphatase cycle. Biochem Biophys Res Commun 131: 899-904.

17. Okar DA, Manzano A, Navarro-Sabate A, Riera L, Bartrons R, et al. (2001) PFK-2/FBPase-2: maker and breaker of the essential biofactor fructose-2,6-bisphosphate. Trends Biochem Sci 26: 30-35.

18. Beutler E (1971) 2,3-diphosphoglycerate affects enzymes of glucose metabolism in red blood cells. Nat New Biol 232: 20-21.

19. Beutler E (1984) Red Cell Metabolism: A Manual of Biochemical Methods. New York: Grune and Stratton.

20. Mehler AH (1963) Kinetic properties of native and carboxy-peptidase-altered rabbit muscle aldolase. J Biol Chem 238: 100-104.

21. Mehler AH, Bloom B (1963) Interaction between rabbit muscle aldolase and dihydroxyacetone phosphate. J Biol Chem 238: 105-107.

22. Penhoet EE, Kochman M, Rutter WJ (1969) Ioslation of fructose diphosphate aldolases A, B, and C. Biochemistry 8: 4391-4395.

23. Penhoet EE, Kochman M, Rutter WJ (1969) Molecular and catalytic properties of aldolase C. Biochemistry 8: 4396-4402.

24. Rose IA, O'Connell EL, Mehler AH (1965) Mechanism of the Aldolase Reaction. J Biol Chem 240: 1758-1765.

25. Srivastava SK, Beutler E (1972) The effect of normal red cell constituents on the activities of red cell enzymes. Arch Biochem Biophys 148: 249-255.

26. Strapazon E, Steck TL (1977) Interaction of the aldolase and the membrane of human erythrocytes. Biochemistry 16: 2966-2971.

27. Yeltman DR, Harris BG (1977) Purification and characterization of aldolase from human erythrocytes. Biochim Biophys Acta 484: 188-198.

28. Gracy RW (1975) Triosephosphate isomerase from human erythrocytes. Methods Enzymol 41: 442-447.

29. Sawyer TH, Tilley BE, Gracy RW (1972) Studies on human triosephosphate isomerase. II. Nature of the electrophoretic multiplicity in erythrocytes. J Biol Chem 247: 6499-6505.

30. Schneider AS, Valentine WN, Hattori M, Heins HL, Jr. (1965) Hereditary Hemolytic Anemia with Triosephosphate Isomerase Deficiency. N Engl J Med 272: 229-235.

31. Cori CF, Velick SF, Cori GT (1950) The combination of diphosphopyridine nucleotide with glyceraldehyde phosphate dehydrogenase. Biochim Biophys Acta 4: 160-169.

32. Furfine CS, Velick SF (1965) The Acyl-Enzyme Intermediate and the Kinetic Mechanism of the Glyceraldehyde 3-Phosphate Dehydrogenase Reaction. J Biol Chem 240: 844-855.

33. Heinz F, Freimuller B (1982) Glyceraldehyde-3-phosphate dehydrogenase from human tissues. Methods Enzymol 89 Pt D: 301-305.

34. Wang CS, Alaupovic P (1980) Glyceraldehyde-3-phosphate dehydrogenase from human erythrocyte membranes. Kinetic mechanism and competitive substrate inhibition by glyceraldehyde 3-phosphate. Arch Biochem Biophys 205: 136-145.

35. Ali M, Brownstone YS (1976) A study of phosphoglycerate kinase in human erythrocytes. II. Kinetic properties. Biochim Biophys Acta 445: 89-103.

36. Krietsch WK, Bucher T (1970) 3-phosphoglycerate kinase from rabbit sceletal muscle and yeast. Eur J Biochem 17: 568-580.

37. Lee CS, O'Sullivan WJ (1975) Properties and mechanism of human erythrocyte phosphoglycerate kinase. J Biol Chem 250: 1275-1281.

38. Yoshida A, Watanabe S (1972) Human phosphoglycerate kinase. I. Crystallization and characterization of normal enzyme. J Biol Chem 247: 440-445.

39. Mulquiney PJ, Bubb WA, Kuchel PW (1999) Model of 2,3-bisphosphoglycerate metabolism in the human erythrocyte based on detailed enzyme kinetic equations: in vivo kinetic characterization of 2,3-bisphosphoglycerate synthase/phosphatase using 13C and 31P NMR. Biochem J 342 Pt 3: 567-580.

40. Mulquiney PJ, Kuchel PW (1999) Model of 2,3-bisphosphoglycerate metabolism in the human erythrocyte based on detailed enzyme kinetic equations: computer simulation and metabolic control analysis. Biochem J 342 Pt 3: 597-604.

41. Garfinkel L, Garfinkel D (1985) Magnesium regulation of the glycolytic pathway and the enzymes involved. Magnesium 4: 60-72.

42. Rider CC, Taylor CB (1974) Enolase isoenzymes in rat tissues. Electrophoretic, chromatographic, immunological and kinetic properties. Biochim Biophys Acta 365: 285-300.

43. Wold F, Ballou CE (1957) Studies on the enzyme enolase. I. Equilibrium studies. J Biol Chem 227: 301-312.

44. Albe KR, Butler MH, Wright BE (1990) Cellular concentrations of enzymes and their substrates. J Theor Biol 143: 163-195.

45. Holzhutter HG, Jacobasch G, Bisdorff A (1985) Mathematical modelling of metabolic pathways affected by an enzyme deficiency. A mathematical model of glycolysis in normal and pyruvate-kinase-deficient red blood cells. Eur J Biochem 149: 101-111.

46. Kahn A, Marie J (1982) Pyruvate kinases from human erythrocytes and liver. Methods Enzymol 90 Pt E: 131-140.

47. Koster JF, Slee RG, Staal GE, van Berkel TJ (1972) The influence of glucose I,6-diphosphate on the enzymatic activity of pyruvate kinase. Biochim Biophys Acta 258: 763-768.

48. Mc QJ, Utter MF (1959) Equilibrium and kinetic studies of the pyruvic kinase reaction. J Biol Chem 234: 2151-2157.

49. Rozengurt E, Jimenez de Asua L, Carminatti H (1969) Some kinetic properties of liver pyruvate kinase (type L). II. Effect of pH on its allosteric behavior. J Biol Chem 244: 3142-3147.

50. Borgmann U, Moon TW, Laidler KJ (1974) Molecular kinetics of beef heart lactate dehydrogenase. Biochemistry 13: 5152-5158.

51. Wang CS (1977) Inhibition of human erythrocyte lactate dehydrogenase by high concentrations of pyruvate. Evidence for the competitive substrate inhibition. Eur J Biochem 78: 569-574.

52. Zewe V, Fromm HJ (1965) Kinetic Studies of Rabbit Muscle Lactate Dehydrogenase. Ii. Mechanism of the Reaction. Biochemistry 4: 782-792.

53. Kanji MI, Toews ML, Carper WR (1976) A kinetic study of glucose-6-phosphate dehydrogenase. J Biol Chem 251: 2258-2262.

54. Thorburn DR, Kuchel PW (1985) Regulation of the human-erythrocyte hexose-monophosphate shunt under conditions of oxidative stress. A study using NMR spectroscopy, a kinetic isotope effect, a reconstituted system and computer simulation. Eur J Biochem 150: 371-386.

55. Kirkman HN, Wilson WG, Clemons EH (1980) Regulation of glucose-6-phosphate dehydrogenase. I. Intact red cells. J Lab Clin Med 95: 877-887.

56. Glaser L, Brown DH (1955) Purification and properties of d-glucose-6-phosphate dehydrogenase. J Biol Chem 216: 67-79.

57. Villet RH, Dalziel K (1972) Studies of 6-phosphogluconate dehydrogenase from sheep liver. 2. Kinetics of the oxidative-decarboxylation reaction, coenzyme binding and analyses for metals. Eur J Biochem 27: 251-258.

58. Pearse BM, Rosemeyer MA (1974) Human 6-phosphogluconate dehydrogenase. Purification of the erythrocyte enzyme and the influence of ions and NADPH on its activity. Eur J Biochem 42: 213-223.

59. Villet RH, Dalziel K (1969) The nature of the carbon dioxide substrate and equilibrium constant of the 6-phosphogluconate dehydrogenase reaction. Biochem J 115: 633-638.

60. McIntyre LM, Thorburn DR, Bubb WA, Kuchel PW (1989) Comparison of computer simulations of the F-type and L-type non-oxidative hexose monophosphate shunts with 31P-NMR experimental data from human erythrocytes. Eur J Biochem 180: 399-420.

61. Casazza JP, Veech RL (1986) The interdependence of glycolytic and pentose cycle intermediates in ad libitum fed rats. J Biol Chem 261: 690-698.

62. Wood T (1979) Purification and properties of D-ribulose-5-phosphate 3-epimerase from calf liver. Biochim Biophys Acta 570: 352-362.

63. Horecker BL, Hurwitz J (1956) The purification of phosphoketopentoepimerase from Lactobacillus pentosus and the preparation of xylulose 5-phosphate. J Biol Chem 223: 993-1008.

64. Urivetzky M, Tsuboi KK (1963) Enzymes of the Human Erythrocyte. V. Pentose Phosphate Isomerase, Purification and Properties. Arch Biochem Biophys 103: 1-8.

65. Kiely ME, Stuart AL, Wood T (1973) Partial purification and kinetic properties of ribose-5-phosphate ketol-isomerase and ribulose-5-phosphate 3-epimerase from various sources. Biochim Biophys Acta 293: 534-541.

66. Joshi A, Palsson BO (1990) Metabolic dynamics in the human red cell. Part III--Metabolic reaction rates. J Theor Biol 142: 41-68.

67. Warnock LG, Prudhomme CR (1982) The isolation and preliminary characterization of apotransketolase from human erythrocytes. Biochem Biophys Res Commun 106: 719-723.

68. Venkataraman R, Racker E (1961) Mechanism of action of transaldolase. II. The substrate-enzyme intermediate. J Biol Chem 236: 1883-1886.

69. Horecker BL, Smyrniotis PZ (1955) Purification and properties of yeast transaldolase. J Biol Chem 212: 811-825.

70. Kuhn E, Brand K (1972) Purification and properties of transaldolase from bovine mammary gland. Biochemistry 11: 1767-1772.

71. Datta AG, Racker E (1961) Mechanism of action of transketolase. I. Properties of the crystalline yeast enzyme. J Biol Chem 236: 617-623.

72. Mannervik B (1973) A branching reaction mechanism of glutathione reductase. Biochem Biophys Res Commun 53: 1151-1158.

73. Worthington DJ, Rosemeyer MA (1976) Glutathione reductase from human erythrocytes. Catalytic properties and aggregation. Eur J Biochem 67: 231-238.

74. Scott EM, Duncan IW, Ekstrand V (1963) Purification and Properties of Glutathione Reductase of Human Erythrocytes. J Biol Chem 238: 3928-3933.

75. Wu F, Yang F, Vinnakota KC, Beard DA (2007) Computer modeling of mitochondrial tricarboxylic acid cycle, oxidative phosphorylation, metabolite transport, and electrophysiology. J Biol Chem 282: 24525-24537.

76. Kohn MC, Achs MJ, Garfinkel D (1979) Computer simulation of metabolism in pyruvate-perfused rat heart. III. Pyruvate dehydrogenase. Am J Physiol 237: R167-173.

77. Tsai CS, Burgett MW, Reed LJ (1973) Alpha-keto acid dehydrogenase complexes. XX. A kinetic study of the pyruvate dehydrogenase complex from bovine kidney. J Biol Chem 248: 8348-8352.

78. Kohn MC, Achs MJ, Garfinkel D (1979) Computer simulation of metabolism in pyruvate-perfused rat heart. II. Krebs cycle. Am J Physiol 237: R159-166.

79. Kohn MC, Garfinkel D (1983) Computer simulation of metabolism in palmitate-perfused rat heart. II. Behavior of complete model. Ann Biomed Eng 11: 511-531.

80. Shepherd D, Garland PB (1969) The kinetic properties of citrate synthase from rat liver mitochondria. Biochem J 114: 597-610.

81. Smith CM, Williamson JR (1971) Inhibition of citrate synthase by succinyl-CoA and other metabolites. FEBS Lett 18: 35-38.

82. Thomson JF, Nance SL, Bush KJ, Szczepanik PA (1966) Isotope and solvent effects of deuterium on aconitase. Arch Biochem Biophys 117: 65-74.

83. Denton RM, Richards DA, Chin JG (1978) Calcium ions and the regulation of NAD+-linked isocitrate dehydrogenase from the mitochondria of rat heart and other tissues. Biochem J 176: 899-906.

84. Plaut GW, Cheung CP, Suhadolnik RJ, Aogaichi T (1979) Cosubstrate and allosteric modifier activities of structural analogues of NAD and ADP for NAD-specific isocitrate dehydrogenase from bovine heart. Biochemistry 18: 3430-3438.

85. Williamson JR, Safer B, LaNoue KF, Smith CM, Walajtys E (1973) Mitochondrial-cytosolic interactions in cardiac tissue: role of the malate-aspartate cycle in the removal of glycolytic NADH from the cytosol. Symp Soc Exp Biol 27: 241-281.

86. Smith CM, Bryla J, Williamson JR (1974) Regulation of mitochondrial alpha-ketoglutarate metabolism by product inhibition at alpha-ketoglutarate dehydrogenase. J Biol Chem 249: 1497-1505.

87. Cha S, Parks RE, Jr. (1964) Succinic Thiokinase. Ii. Kinetic Studies: Initial Velocity, Product Inhibition, and Effect of Arsenate. J Biol Chem 239: 1968-1977.

88. Barman TE (1969) Enzyme Handbook. New York: Springer-Verlag.

89. Gutman M (1977) Regulation of mitochondrial succinate dehydrogenase by substrate type activators. Biochemistry 16: 3067-3072.

90. Hatefi Y, Stiggall DL (1976) Metal-containing flavoprotein dehydrogenases. In: Boyer PD, editor. The Enzymes. New York: Academic Press. pp. 175-297.

91. Brant DA, Barnett LB, Alberty RA (1963) The temperature dependence of the steady state kinetic parameters of the fumarase reaction. J Am Chem Soc 85: 2204-2209.

92. Penner PE, Cohen LH (1969) Effects of adenosine triphosphate and magnesium ions on the fumarase reaction. J Biol Chem 244: 1070-1075.

93. Kimball DF, Peterson L, McLoughlin DJ, Wolfe RG (1979) Malate dehydrogenase. Kinetic studies with meso-tartrate and 2-keto-3-hydroxysuccinate, comparison of the mitochondrial and supernatant pig heart enzymes. Arch Biochem Biophys 195: 66-73.

94. Oza NB, Shore JD (1973) The effects of adenine nucleotides on NADH binding to mitochondrial malate dehydrogenase. Arch Biochem Biophys 154: 360-365.

95. Henson CP, Cleland WW (1964) Kinetic Studies of Glutamic Oxaloacetic Transaminase Isozymes. Biochemistry 3: 338-345.

96. Crow KE, Braggins TJ, Batt RD, Hardman MJ (1982) Rat liver cytosolic malate dehydrogenase: purification, kinetic properties, role in control of free cytosolic NADH concentration. Analysis of control of ethanol metabolism using computer simulation. J Biol Chem 257: 14217-14225.

97. Indiveri C, Dierks T, Kramer R, Palmieri F (1991) Reaction mechanism of the reconstituted oxoglutarate carrier from bovine heart mitochondria. Eur J Biochem 198: 339-347.

98. Dierks T, Riemer E, Kramer R (1988) Reaction mechanism of the reconstituted aspartate/glutamate carrier from bovine heart mitochondria. Biochim Biophys Acta 943: 231-244.

99. Uldry M, Thorens B (2004) The SLC2 family of facilitated hexose and polyol transporters. Pflugers Arch 447: 480-489.

100. Haser WG, Shapiro RA, Curthoys NP (1985) Comparison of the phosphate-dependent glutaminase obtained from rat brain and kidney. Biochem J 229: 399-408.

101. Rife JE, Cleland WW (1980) Kinetic mechanism of glutamate dehydrogenase. Biochemistry 19: 2321-2328.

102. Frieden C (1959) Glutamic dehydrogenase. III. The order of substrate addition in the enzymatic reaction. J Biol Chem 234: 2891-2896.

103. Houston B, Nimmo HG (1985) Effects of phosphorylation on the kinetic properties of rat liver ATP-citrate lyase. Biochim Biophys Acta 844: 233-239.

104. Plowman DM, Cleland WW (1967) Purification and kinetic studies of the citrate cleavage enzyme. J Biol Chem 242: 4239-4247.

105. Ranganathan NS, Srere PA, Linn TC (1980) Comparison of phospho- and dephospho-ATP citrate lyase. Arch Biochem Biophys 204: 52-58.

106. Houston B, Nimmo HG (1984) Purification and some kinetic properties of rat liver ATP citrate lyase. Biochem J 224: 437-443.

107. Teller JK, Fahien LA, Davis JW (1992) Kinetics and regulation of hepatoma mitochondrial NAD(P) malic enzyme. J Biol Chem 267: 10423-10432.

108. Hsu RY, Lardy HA, Cleland WW (1967) Pigeon liver malic enzyme. V. Kinetic studies. J Biol Chem 242: 5315-5322.

109. Bulos B, Handler P (1965) Kinetics of Beef Heart Glutamic-Alanine Transaminase. J Biol Chem 240: 3283-3294.

110. Jitrapakdee S, Walker ME, Wallace JC (1999) Functional expression, purification, and characterization of recombinant human pyruvate carboxylase. Biochem Biophys Res Commun 266: 512-517.

111. Halestrap AP, Meredith D (2004) The SLC16 gene family-from monocarboxylate transporters (MCTs) to aromatic amino acid transporters and beyond. Pflugers Arch 447: 619-628.

112. Juel C, Halestrap AP (1999) Lactate transport in skeletal muscle - role and regulation of the monocarboxylate transporter. J Physiol 517 ( Pt 3): 633-642.
